# Plant-derived modulators of the hemostasis-wound healing axis: mechanisms, evidence, and translational potential

**DOI:** 10.3389/fphar.2026.1902225

**Published:** 2026-09-29

**Authors:** Zoila Mora-Guzmán, Ibzan Jahzeel Salvador Ibarra, Lilian Dolores Chel‐Guerrero, José Pedro Elizalde Díaz, María Del Socorro Pina-Canseco, Daniel Gómez Hernández, Eduardo Pérez-Campos, Hector Alejandro Cabrera-Fuentes, María Teresa Hernández-Huerta

**Affiliations:** 1 SECIHTI, Faculty of Medicine and Surgery, Autonomous University “Benito Juárez” of Oaxaca, Oaxaca, Mexico; 2 Centro de Investigación Facultad de Medicina UNAM-UABJO, Facultad de Medicina y Cirugía, Universidad Autónoma “Benito Juárez” de Oaxaca, Oaxaca, Mexico; 3 Instituto Mexicano del Seguro Social, Hospital General de Zona con Medicina Familiar No. 18, Coahuila de Zaragoza, Mexico; 4 Tecnológico Nacional de México, Instituto Tecnológico de Mérida, Yucatán, Mexico; 5 Centro Médico Nacional “20 de Noviembre”, ISSSTE, Ciudad de México, Mexico; 6 División de Estudios de Posgrado e Investigación, Tecnológico Nacional de México, Instituto Tecnológico de Oaxaca, Oaxaca, Mexico; 7 Hospital General de Zona No. 1 Dr. Demetrio Mayoral Pardo, Instituto Mexicano del Seguro Social (IMSS), Oaxaca, Mexico; 8 R&D Group, Vice Presidency for Scientific Research and Innovation, Imam Abdulrahman Bin Faisal University, Dammam, Saudi Arabia; 9 División de Estudios de Posgrado e Investigación, Tecnológico Nacional de México, Instituto Tecnológico de Tijuana, Tijuana, Baja California, Mexico

**Keywords:** botanical preparations, hemostasis, herbal medicine, medicinal plants, procoagulant, translational medicine, wound healing

## Abstract

Medicinal plants are widely used to control bleeding and support wound care, but the hemostatic and reparative evidence attributed to a species often derives from different plant parts, extracts, or formulations. This critical narrative review examines the ethnomedicinal, experimental, clinical, safety, and translational evidence for plant-derived preparations reported to modulate one or more stages of the hemostasis-wound-healing axis. Structured searches of PubMed/MEDLINE, Scopus, and Web of Science were updated through 19 July 2026 using terms related to medicinal plants, coagulation, platelet function, wound healing, formulation, standardization, toxicity, and safety. The literature retained for detailed synthesis encompassed crude extracts, enriched fractions, latex-derived proteases, isolated plant metabolites, hydrogels, nanoparticles, and other formulated materials. Mechanistically, direct evidence was concentrated primarily in fibrinogen-cleaving plant proteases and notoginsenoside Ft1–mediated platelet signaling. In contrast, anti-inflammatory, pro-angiogenic, antimicrobial, and extracellular-matrix-related effects were supported mainly at the extract, fraction, or formulation level. The strength and mechanistic depth of the evidence varied substantially among species, preparations, experimental models, and outcomes, and hemostatic and wound-repair effects were frequently demonstrated using different materials from the same species. Thus, the available evidence supports the hemostasis-wound-healing axis as a useful framework for prioritizing candidates, but current evidence does not support medicinal plants as a uniformly effective therapeutic class. Translation should initially prioritize chemically and biologically standardized topical or locally retained products, component-specific controls, clinically relevant preclinical models, exposure–response and safety characterization, and adequately powered indication-specific trials. Taxonomic validity, material traceability, sustainable sourcing, and ethical governance of traditional knowledge should be integrated from the outset.

## Graphical Abstract

**Figure:**
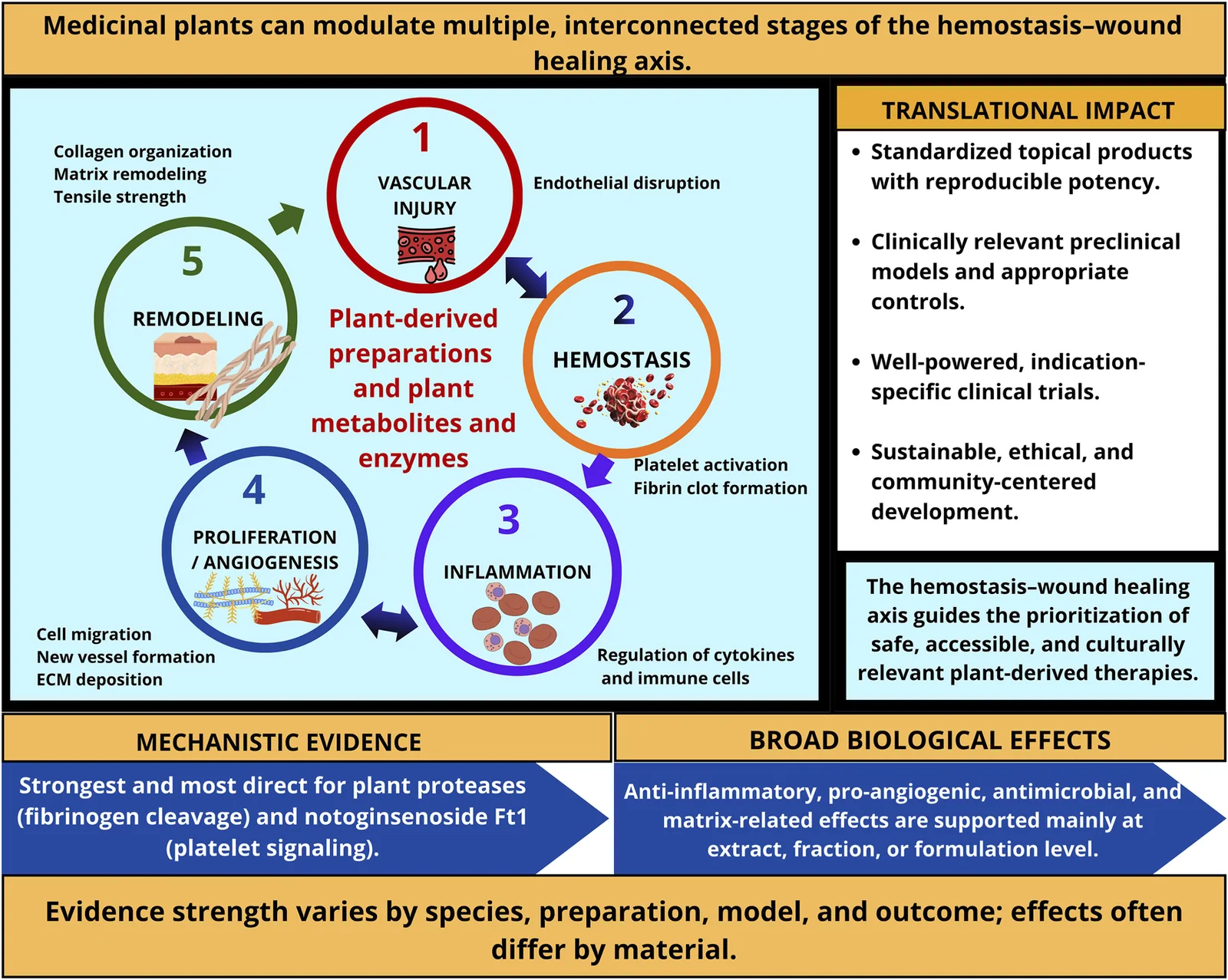
Plant-derived preparations and bioactive compounds may modulate interconnected stages of the hemostasis–wound-healing axis, from clot formation to inflammation, angiogenesis, and remodeling. Translation requires preparation-specific mechanistic validation, standardized topical formulations, rigorous preclinical and clinical assessment, and sustainable, ethical development.

## 1 Introduction

Acute bleeding and chronic wounds remain persistent and globally significant clinical challenges, both in hospital and community settings (Mamun et al., 2024). In trauma and surgery, uncontrolled bleeding continues to be a leading preventable cause of mortality in the first few hours after injury, underscoring the need for effective and accessible hemostatic control strategies (Jones et al., 2023; Miller et al., 2024; Bernhardt et al., 2025). In parallel, chronic wounds, including those associated with diabetes and vascular insufficiency, impose a considerable clinical, economic, and psychosocial burden, with a direct impact on quality of life and healthcare resource utilization (Sen, 2021; Aditya et al., 2025; Díaz-Herrera et al., 2025). Despite advances in topical hemostatic agents, dressings, and regenerative therapies, access to these interventions remains uneven, particularly in rural areas and low- and middle-income settings, where traditional and complementary medicine continues to play an important role in primary healthcare because of its availability, affordability, and cultural embeddedness (Ahn et al., 2025; Mamun et al., 2024; Díaz-Herrera et al., 2025).

The use of medicinal plants for bleeding control and wound-healing promotion is among the oldest and most consistently documented therapeutic practices across multiple traditional medical systems (Sen, 2021). The relevance of these practices is plausible because hemostasis not only limits blood loss but also initiates a bioactive microenvironment: the fibrin clot and platelets form a provisional matrix that concentrates and releases mediators (e.g., growth factors and cytokines) capable of modulating early inflammation, cell migration, and the onset of the proliferative phase (Kearney et al., 2021; Wilkinson and Hardman, 2020). This interdependence suggests that agents capable of enhancing local hemostatic control may support the early stages of tissue repair, provided that clot formation, tissue perfusion, inflammation, and fibrinolysis remain appropriately regulated. Ethnomedicinal descriptions of plants with “astringent,” “wound-closing,” or styptic properties acquire renewed mechanistic relevance. Such traditional uses may reflect local protein precipitation, reduction of exudation, tissue contraction, strengthening of the provisional wound matrix, or direct effects on platelet activation and fibrin formation (Swieringa et al., 2018; Quave, 2018). Ethnopharmacological and experimental studies have begun to validate these applications, showing in preclinical models effects on platelet aggregation, activation of procoagulant pathways, and acceleration of wound closure, including evidence for plant proteases with thrombin-like activity and extracts with hemostatic and proregenerative properties (Quave, 2018; Patwardhan and Aswar, 2025).

Although plant-based therapies have been widely studied for wound healing or hemostatic control, these processes are still commonly treated as separate therapeutic domains (Pazyar et al., 2014; Molefe et al., 2025; Abdallah et al., 2022). However, current understanding of fibrin biology, platelet signaling, and immunothrombosis supports a more integrated view in which coagulation is not merely a terminal event in bleeding control, but an early and biologically instructive phase of tissue repair (Bayer, 2022; De Nardi et al., 2024; Marcos-Jubilar et al., 2023). From this perspective, medicinal plants with dual procoagulant and wound-healing activity represent a particularly valuable field of study, both for validating traditional knowledge and for identifying multifunctional candidates for translational development (Li et al., 2024; Li et al., 2025; Urs et al., 2019; Lee et al., 2025).

To address this gap, we conducted a critical narrative review informed by a structured and iterative literature search. Accordingly, this review asks: at what material and mechanistic levels do plant-derived preparations show direct hemostatic or wound-repair effects, and which methodological, safety, and standardization limitations constrain their translation? The review integrates physiological, ethnomedicinal, experimental, clinical, safety, and translational evidence concerning plant-derived preparations with reported effects on hemostasis or wound healing. To address this question, we examine the physiological links between hemostasis and tissue repair, stratify the evidence according to the tested material and degree of mechanistic validation, and critically evaluate the limitations affecting translational development. The detailed methodological approach, including information sources, literature-identification principles, source prioritization, and evidence classification, is described in Section 2. By framing hemostasis and wound healing as interconnected components of an integrated biological axis, this review seeks to move beyond descriptive cataloging toward a mechanistically grounded and translationally oriented interpretation of medicinal plants used in bleeding control and wound care.

## 2 Methods

### 2.1 Review design

This article was developed as a critical narrative review informed by a structured literature search. The review was not intended to constitute an exhaustive systematic review or to quantitatively aggregate all available studies. Its purpose was to critically integrate physiological, ethnomedicinal, experimental, clinical, safety, and translational evidence relevant to plant-derived modulation of the hemostasis–wound-healing axis. A narrative approach was considered appropriate because the available literature encompasses highly heterogeneous plant components and materials, including crude extracts, enriched fractions, latex-derived enzymes, isolated metabolites, topical formulations, hydrogels, and other biomaterials, as well as diverse experimental models, comparators, doses, and outcome measures. This diversity precluded quantitative pooling and favored a concept-oriented synthesis focused on biological plausibility, mechanistic depth, consistency of findings, and translational relevance. Methodological reporting was informed by the principles of the Scale for the Assessment of Narrative Review Articles (SANRA) (Baethge et al., 2019).

### 2.2 Literature identification

A literature search was conducted in PubMed/MEDLINE and cross-referenced with Scopus and Web of Science from the databases’ inception until 19 July 2026, without applying publication year limits or language restrictions during the search phase. The search terms covered four domains: a) medicinal plants and botanical preparations; b) hemostasis and coagulation; c) wound healing and tissue repair; and d) safety and translational development. Controlled vocabulary and free text terms were combined using Boolean operators, adapted to the indexing and syntax of each database. Additional targeted searches were conducted for species, preparations, components, or mechanisms identified during manuscript development. These searches were used to identify potentially relevant primary publications and were subjected to the same eligibility and evaluation criteria described below. The literature identification and selection process is summarized in Figure 1.

**FIGURE 1:**
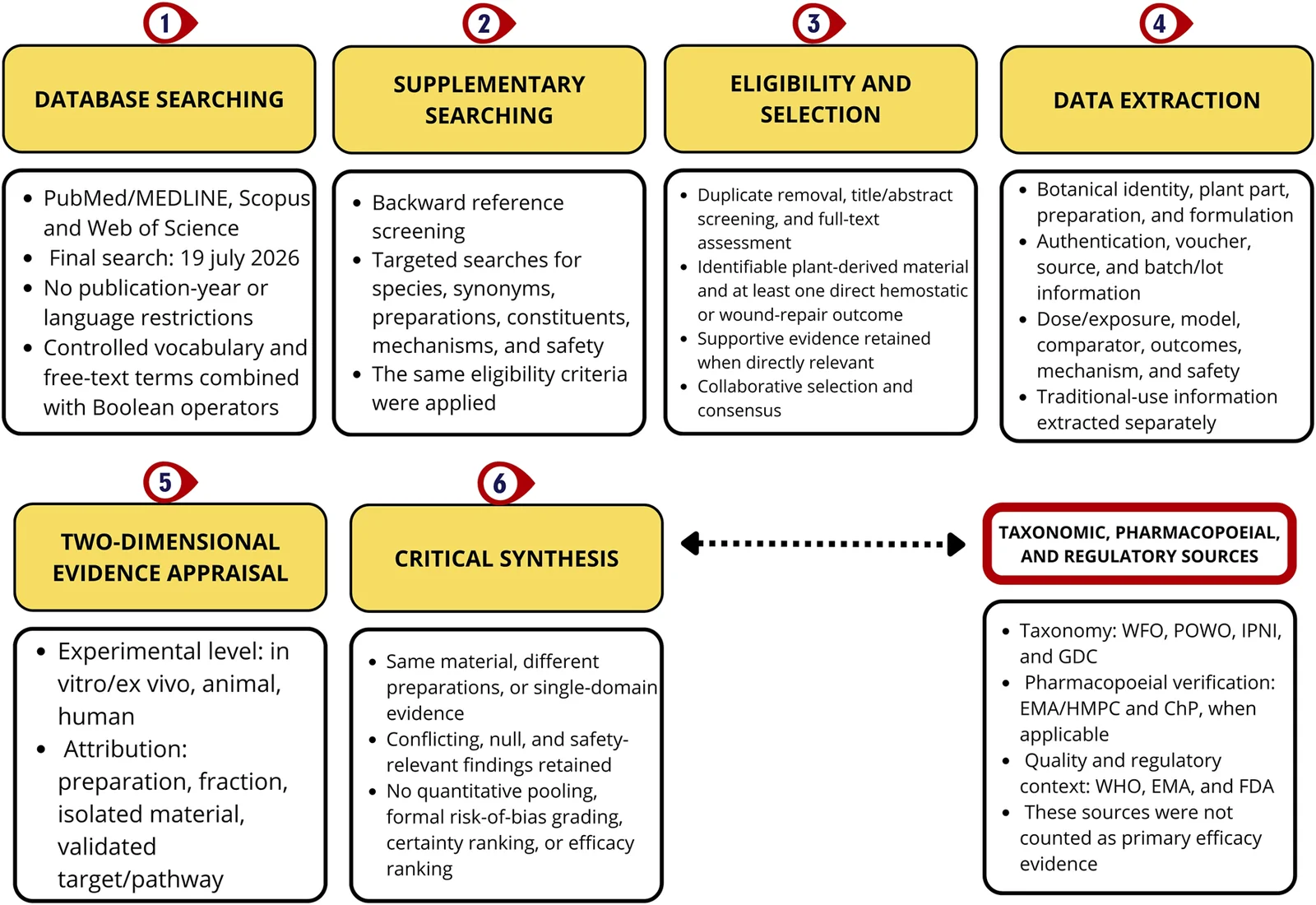
Methodological workflow for the structured critical narrative review.

### 2.3 Eligibility criteria and source selection

Primary experimental or clinical studies that evaluated an identifiable plant material, including a crude extract, fraction, isolated metabolite, purified enzyme, or formulation, and reported at least one direct hemostatic or wound-healing outcome were included. Supporting studies addressing antimicrobial activity, inflammation, formulation performance, safety, pharmacokinetics, or quality control were retained when directly relevant to an included botanical candidate. Reviews, official monographs, pharmacopoeias, and regulatory documents were used for contextual purposes only. Studies limited to phytochemical composition, antioxidant assays, or computational predictions, without a corresponding functional outcome, were not considered evidence of efficacy. Multicomponent formulations were included when their botanical components and overall composition could be identified; effects were attributed to the complete formulation unless component-specific controls supported a more restricted attribution. Study selection was conducted collaboratively by ZM-G, IJSI, and MTH-H, and inclusion decisions were reached by consensus.

### 2.4 Data extraction, evidence classification, and critical appraisal

For each selected study, we extracted the botanical identity, plant part, preparation or formulation, authentication and traceability information, dose or concentration, administration conditions, experimental model, comparator, relevant results, mechanistic evidence, and safety findings. Studies were organized around the hemostasis-wound healing axis and classified according to whether hemostatic and reparative effects were demonstrated using the same material, different preparations of the same species, or a material evaluated in only one domain. Experimental evidence was categorized as *in vitro*, *ex vivo*, animal *in vivo*, or human evidence. Material and mechanistic attribution was classified as complete extract or formulation, active or enriched fraction, isolated metabolite, purified enzyme, molecular target, or functionally validated signaling pathway. Critical appraisal considered botanical and material traceability, preparation definition, comparator and model suitability, outcome directionality, dose-response relationship assessment, component-specific controls, mechanistic validation, safety, and appropriateness of attribution. Effects were attributed to the entire analyzed material unless direct experiments supported attribution to a specific fraction, compound, enzyme, target, or metabolic pathway. Missing information was recorded as NR, and non-applicable items as N/A. Study-level findings are summarized in Supplementary Table S2, taxonomic verification in Supplementary Table S3, material traceability in Supplementary Table S4, and mechanistic attribution in Figure 2. No quantitative pooling, formal risk-of-bias assessment, or certainty-of-evidence ranking was performed.

**FIGURE 2:**
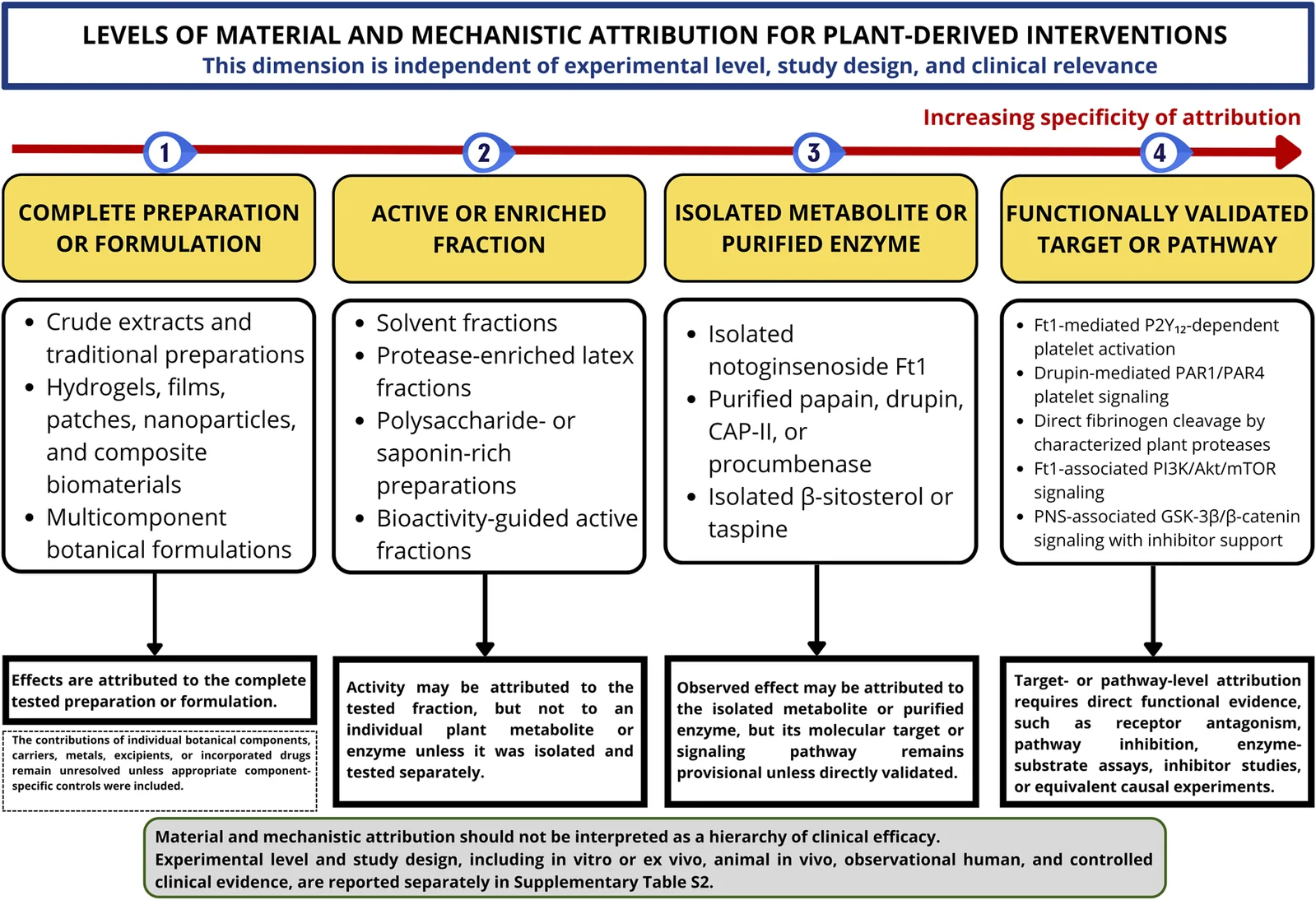
Levels of material and mechanistic attribution for plant-derived interventions across the hemostasis–wound-healing axis. Abbreviations: CAP-II, protease II isolated from *Curcuma aromatica* Salisb. [Zingiberaceae]; Ft1, notoginsenoside Ft1; PAR1/PAR4, protease-activated receptors 1 and 4; P2Y_12_, platelet P2Y_12_ purinergic receptor; PI3K/Akt/mTOR, phosphoinositide 3-kinase/protein kinase B/mechanistic target of rapamycin; PNS, total saponins from *Panax notoginseng* (Burkill) F.H.Chen [Araliaceae].

### 2.5 Taxonomic verification

Plant names were initially recorded as they appeared in the original publications to preserve traceability at the article level. Accepted botanical names, full author citations, family assignments, and World Flora Online (WFO; https://www.worldfloraonline.org/) identifiers were verified against the World Flora Online Plant List, version 2026-06 (accessed 25 July 2026). Plants of the World Online (POWO; https://powo.science.kew.org/) was used to check acceptance status and synonymy, and the Global Compositae Database/Checklist (GDC; https://www.compositae.org/gcd) (accessed 25 July 2026) was additionally consulted for species belonging to Asteraceae. WFO was used as the primary nomenclatural basis for the Supplementary Tables S2–S5. Standardized nomenclature and author abbreviations were checked against the International Plant Names Index (IPNI), and author citations followed the International Code of Nomenclature for algae, fungi, and plants. Official botanical or pharmacopoeial drug names were included only when a recognized designation could be linked to the species and relevant plant part, using current European Medicines Agency/Committee on Herbal Medicinal Products (EMA/HMPC; https://www.ema.europa.eu/) monographs. A pharmacopoeial designation was not interpreted as evidence that the extract, fraction, essential oil, or experimental formulation complied with the corresponding monograph. Each botanical component of a multicomponent formulation was evaluated separately. Accordingly, *Bletilla striata* (Thunb.) Rchb.f. [Orchidaceae] and *Dittrichia viscosa* (L.) Greuter [Asteraceae], reported in the original publication as *Inula viscosa* (L.) Aiton, were included in Supplementary Tables S3 and S4. The findings of these studies were attributed to the complete formulation unless component-specific controls supported a more precise attribution. Study-specific information on botanical authentication, reference specimens, material origin, supplier, and lot number was extracted from the original publications and recorded in Supplementary Table S4. Missing information was recorded as NR. Additionally, information on traditional use was extracted separately and is summarized in Supplementary Table S5.

### 2.6 Limitations of the methodological approach

The design of this study allowed for the integration of heterogeneous evidence (mechanistic, ethnopharmacological, experimental, and clinical) but also required our judgment in identifying and prioritizing publications. Therefore, this review should not be interpreted as an exhaustive inventory of all plant species or all studies related to hemostasis and wound healing. For greater transparency, the databases, search terms, selection criteria, evidence classification framework, and date of the final search are specified.

## 3 Physiological basis of the hemostasis–wound-healing axis

Hemostasis, inflammation, proliferation, and remodeling are temporally overlapping phases coordinated by reciprocal cellular and molecular signaling. Hemostasis begins immediately after vascular injury and limits blood loss through transient vasoconstriction, primary hemostasis, secondary hemostasis, and regulated fibrinolysis. Its function extends beyond hemorrhage control: the platelet- and fibrin-rich hemostatic clot establishes a provisional extracellular matrix (ECM) and a localized signaling environment that promotes inflammatory cell recruitment, cell migration, angiogenesis, re-epithelialization, and subsequent matrix remodeling (Bayer, 2022; Wilkinson and Hardman, 2020; Kearney et al., 2021). Following vascular rupture, transient vasoconstriction reduces local blood flow, while endothelial injury exposes subendothelial collagen and von Willebrand factor (vWF), facilitating the interaction of circulating coagulation factors with tissue factor in the vessel wall (Figure 3). Platelets are initially anchored by interactions between vWF and the platelet glycoprotein (Gp) complex Ib-IX-V and subsequently adhere tightly and are activated via collagen-sensitive receptors, including Gp VI receptor. Platelet activation induces cytoskeletal reorganization, increased intracellular Ca^2+^, granule secretion, and thromboxane A_2_ (TXA_2_) synthesis. The released adenosine diphosphate (ADP), which acts in part through the P2Y_12_ receptor, along with TXA_2_ and locally generated thrombin, amplifies platelet recruitment and converts αIIbβ3 to its high-affinity state. The binding of fibrinogen to activated αIIbβ3 forms interplatelet bridges and consolidates the primary platelet plug. However, this initial plug remains relatively labile until it is reinforced with fibrin (Chatterjee et al., 2020; Kearney et al., 2021).

**FIGURE 3:**
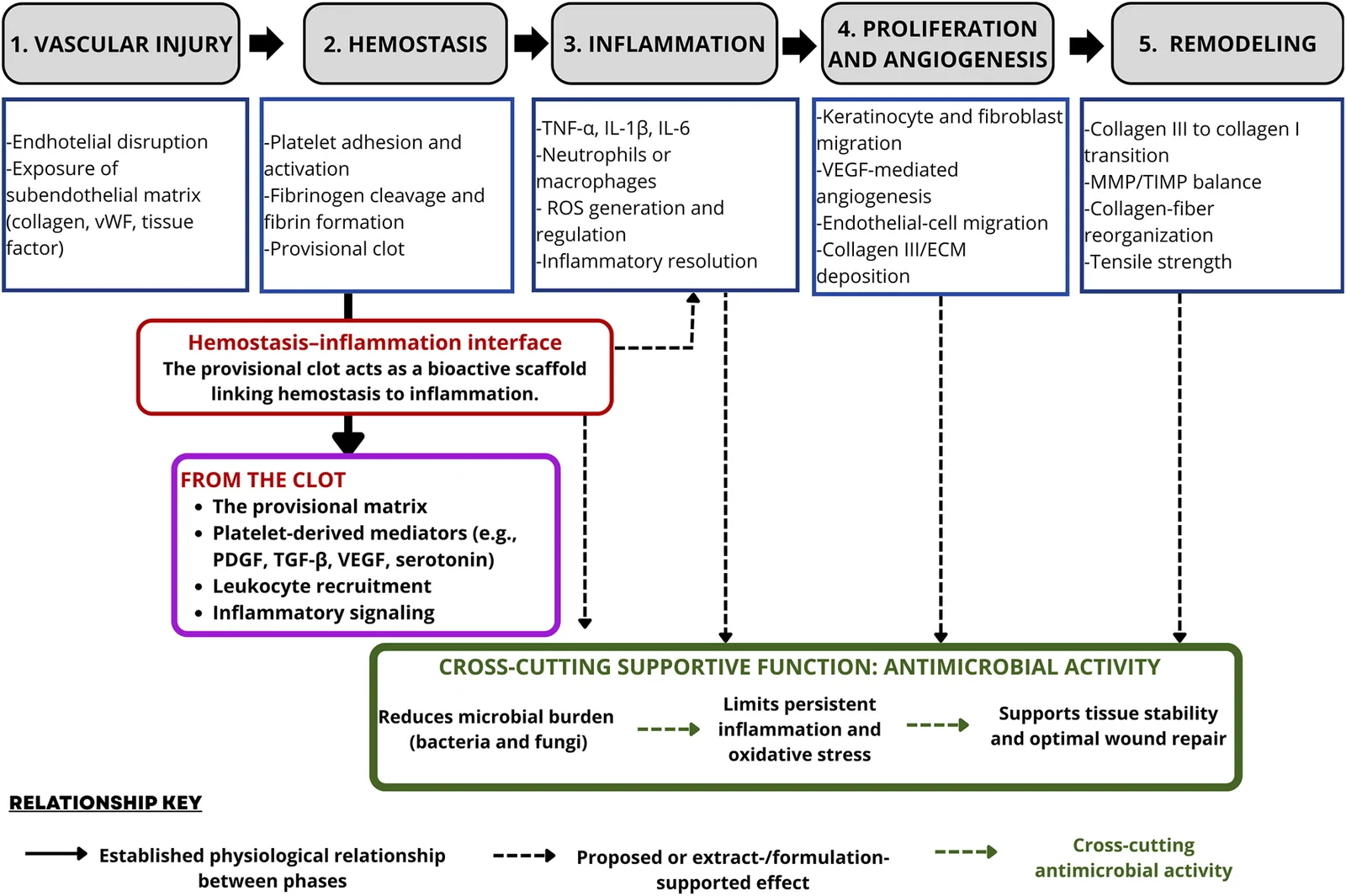
Conceptual framework of the hemostasis–wound-healing axis and the cross-cutting supportive role of antimicrobial activity. Abbreviations: ECM, extracellular matrix; IL-1β, interleukin-1 beta; IL-6, interleukin-6; MMPs, matrix metalloproteinases; PDGF, platelet-derived growth factor; ROS, reactive oxygen species; TGF-β, transforming growth factor beta; TIMPs, tissue inhibitors of metalloproteinases; TNF-α, tumor necrosis factor alpha; VEGF, vascular endothelial growth factor; vWF, von Willebrand factor.

The coagulation initiated by tissue factor and its subsequent amplification converge to generate thrombin. Thrombin converts soluble fibrinogen into fibrin monomers, further activates platelets, and activates factor XIII; factor XIIIa then cross-links the fibrin network, increasing the mechanical stability and strength of the clot. Thrombin acts as a signaling protease via protease-activated receptors, thus linking coagulation to endothelial activation, inflammation, and tissue repair. Activated platelets also release mediators such as platelet-derived growth factor, transforming growth factor β, and vascular endothelial growth factor, which contribute to fibroblast recruitment, angiogenesis, ECM production, and the initiation of the proliferative response (Chatterjee et al., 2020; Wilkinson and Hardman, 2020; Marcos-Jubilar et al., 2023). The hemostatic clot acts as a bioactive interface between coagulation and inflammation. Fibrin, fibrinogen, platelets, and clot-associated mediators provide adhesive and chemotactic signals that promote neutrophil and monocyte/macrophage recruitment to the injured tissue. Neutrophils contribute to the clearance of microorganisms and damaged tissue, while macrophages coordinate debris clearance, regulate the inflammatory response, and facilitate the transition to a reparative microenvironment. These interactions are encompassed by the concept of immunothrombosis, in which platelets, coagulation proteins, endothelial cells, and immune cells cooperate locally to contain tissue damage and infection. When appropriately localized, this response contributes to host defense and repair. However, excessive or persistent thromboinflammatory activity can impair microvascular perfusion, increase tissue hypoxia, and prolong inflammatory injury (Kearney et al., 2021; Marcos-Jubilar et al., 2023; De Nardi et al., 2024). Therefore, effective healing requires not only the onset of inflammation but also its timely resolution and transition to the proliferative phase (Landén et al., 2016; Wilkinson and Hardman, 2020).

As inflammation resolves, the fibrin-rich provisional matrix facilitates integrin-dependent migration of fibroblasts, keratinocytes, and endothelial cells to the wound bed. Fibroblasts produce granulation tissue and extracellular matrix components, keratinocytes restore the epithelial barrier, and the migration and proliferation of endothelial cells promote neovascularization. Simultaneously, growth factors retained or released from the clot help coordinate fibroblast proliferation, collagen synthesis, angiogenesis, and re-epithelialization. Consequently, the reparative value of the clot depends not only on its presence or quantity but also on its composition, architecture, cross-linking, mechanical stability, porosity, and susceptibility to controlled degradation. These properties influence cell infiltration, oxygen and nutrient supply, microbial containment, and the progressive replacement of fibrin by new tissue (Bayer, 2022; Kearney et al., 2021; Wilkinson and Hardman, 2020).

Fibrinolysis begins while proliferative repair is underway, rather than being limited to a terminal event. Tissue-type and urokinase-type plasminogen activators convert plasminogen to plasmin, which degrades fibrin and facilitates matrix turnover, cell migration, and the restoration of vascular patency. This process is critical: early and excessive fibrinolysis can destabilize the clot and promote rebleeding, while insufficient or delayed fibrinolysis can perpetuate inflammation, hinder tissue organization, and contribute to fibrosis. During subsequent remodeling, the provisional matrix is ​​progressively replaced by a collagen-rich extracellular matrix, type III collagen is reorganized and increasingly replaced by type I collagen, and the balance between matrix metalloproteinases and their tissue inhibitors regulates matrix degradation, scar maturation, and recovery of tensile strength (Gill and Parks, 2008; Wilkinson and Hardman, 2020; Risman et al., 2023). In general, the hemostasis-wound healing axis depends on a balanced, spatially restricted, and temporally limited response, rather than on maximum procoagulant activity. Its reparative potential depends on clot architecture and stability, tissue and endothelial compatibility, resolution of inflammation, preservation of local perfusion, controlled fibrinolysis, and progression toward re-epithelialization and matrix remodeling (Kearney et al., 2021; De Nardi et al., 2024; Risman et al., 2023).

## 4 Medicinal-plant evidence across the hemostasis–wound-healing axis

From an ethnopharmacological perspective, the literature retained in this review encompasses plant species with heterogeneous evidence profiles across the hemostasis–wound-healing axis. Within the literature identified through the stated search approach, *Chromolaena odorata* (L.) R.M.King & H.Rob. [Asteraceae] (Pandith et al., 2012; Li et al., 2024), *Tridax procumbens* L. [Asteraceae] (Alshargabi et al., 2023; Gubbiveeranna et al., 2024), *Ageratum conyzoides* L. [Asteraceae] (Li et al., 2025; Kantesaria et al., 2026), and *Panax notoginseng* (Burkill) F.H.Chen [Araliaceae] (Gao et al., 2014) are discussed as representative examples rather than as an exhaustive or ranked set. The extent and mechanistic depth of evidence differ among species, preparations, experimental models, and outcomes, as summarized in Supplementary Tables S1 and S2; accepted botanical names, authorities, and taxonomic identifiers are provided in Supplementary Table S3. Most studies evaluated crude extracts, enriched fractions, latexes, or formulated materials rather than isolated metabolites or purified enzymes. Accordingly, the observed effects are attributed here to the tested preparations. Individual constituents are described as candidate mediators unless their activity has been demonstrated through bioactivity-guided fractionation, isolated-compound experiments, or target-level assays. Comparative statements in this review apply only to the retained literature and should not be interpreted as a ranking among all medicinal plants with reported hemostatic activity.

### 4.1 Experimental and clinical evidence on hemostasis and coagulation

The procoagulant activity of medicinal plants has been evaluated mainly through bleeding time, clotting time, prothrombin time (PT), activated partial thromboplastin time (aPTT), and platelet aggregation assays (Ediriweera et al., 2011; Abdallah et al., 2022; Pandith et al., 2012; Gao et al., 2014). Bleeding time and platelet function assays primarily provide information on primary hemostasis, whereas PT and aPTT offer pathway-specific information on the extrinsic/common and intrinsic/common pathways, respectively. A reduction in PT or aPTT indicates faster clot formation under the experimental conditions but does not identify a specific molecular target (Santoro et al., 2023). Therefore, these assays should be interpreted as functional screening tools and complemented with specific factor assays, thrombin generation tests, fibrinogen cleavage assays, platelet function studies, or inhibitor experiments. Subsequent thrombin generation converts fibrinogen to fibrin, which is then stabilized by factor XIIIa and forms a temporary matrix that limits blood loss and promotes early tissue repair (Wilkinson and Hardman, 2020).

At the preparation level, *C. odorata* (L.) R.M.King & H.Rob. [Asteraceae] preparations shortened bleeding time *in vivo*, whereas platelet and plasma-coagulation findings were weak or discordant *in vitro*; therefore, the underlying hemostatic mechanism remains unresolved (Pandith et al., 2012; Li et al., 2024). *Tridax procumbens* L. [Asteraceae] crude extracts decreased clotting time *in vitro* (Alshargabi et al., 2023), while a silver nanoparticle formulation mediated by *A. conyzoides* L. [Asteraceae] has exhibited hemostatic, antibacterial, and wound-closure effects (Li et al., 2025). In these studies, the observed effects are attributable to the tested preparation rather than to individual phytochemicals, since the evaluations were performed on crude extracts, active fractions, or composite formulations.

Other extract-level studies showed bidirectional coagulation findings. A 70% ethanolic extract of *Agastache rugosa* (Fisch. & C.A.Mey.) Kuntze [Lamiaceae] shortened PT and increased fibrinogen, while some solvent fractions shortened PT or aPTT. In contrast, isolated acacetin and tilianin exhibited anticoagulant activity (Cao et al., 2017). An aqueous decoction of *Scoparia dulcis* L. [Plantaginaceae] administered orally shortened the clotting time of whole blood in rats, but low and high concentrations produced opposite effects *in vitro*, with no changes in PT, aPTT, TT or platelet count (Ediriweera et al., 2011). A lyophilized aqueous extract of *Verbascum sinuatum* L. [Scrophulariaceae] leaves shortened the aPTT without altering the PT, providing preliminary evidence of an effect involving the intrinsic and/or common pathway (Abdallah et al., 2022). *Annona senegalensis* Pers. [Annonaceae] leaf extract counteracted *Bitis rhinoceros* [Viperidae] venom-induced coagulopathy *in vitro*; this context-specific protection should not be interpreted as direct procoagulant activity in normal plasma (Julien et al., 2023).

Limited clinical evidence has been reported regarding the control of bleeding with *Capsella bursa-pastoris* (L.) Medik. [Brassicaceae]. Adjunctive oral capsules reduced heavy menstrual bleeding, and sublingual drops administered with oxytocin as a base reduced early postpartum blood loss. However, these formulation- and regimen-specific findings do not establish independent efficacy or a defined clotting mechanism (Naafe et al., 2018; Ghalandari et al., 2017). In a rat model of hepatic hemorrhage, a topical mixture of *C. bursa-pastoris* and *D. viscosa* reduced blood loss, but the effect was attributed solely to the complete preparation of both plants (Öztürk et al., 2022). A chitosan with gelatin cryogel loaded with *Verbascum thapsus* L. [Scrophulariaceae] leaf extract also improved whole blood coagulation *in vitro*, although the specific contribution of the plant could not be separated from that of the composite biomaterial (Uzuner et al., 2025).

More direct biochemical evidence is available for plant thrombin-like proteases (PTLPs), primarily those obtained from latex-producing species. These enzymes can cleave fibrinogen and promote fibrin formation, partially mimicking thrombin’s action in the final phase of the coagulation cascade. Cysteine protease preparations from *Calotropis gigantea* (L.) W.T.Aiton [Apocynaceae] promoted fibrinogen polymerization and shortened clotting time, while also exhibiting fibrinolytic activity (Rajesh et al., 2005; Bindhu and Singh, 2014). Latex protease preparations from *Tabernaemontana divaricata* (L.) R.Br. ex Roem. & Schult. [Apocynaceae] also promoted fibrin formation and shortened plasma clotting time, although clot-degrading activity was also observed (Singh et al., 2015; Singh et al., 2021). Solanain from *Vallaris solanacea* (Roth ex Roem. & Schult.) Kuntze [Apocynaceae] was biochemically characterized as a cysteine protease, but the corresponding study did not assess a clotting or bleeding endpoint (Somavarapu et al., 2018). Thus, the hemostatic activity reported for *V. solanacea* should be attributed to the protease fraction of the latex evaluated in functional models of coagulation and bleeding, rather than to purified solanain alone (Urs et al., 2019).

Protease-enriched latex fractions from *Carica papaya* L. [Caricaceae], *Jatropha curcas* L. [Euphorbiaceae], *C. gigantea*, *Oxystelma esculentum* (L.f.) Sm. [Apocynaceae], *T. divaricata*, and *V. solanacea* shortened aPTT without altering PT and accelerated clotting in factor VIII–deficient plasma (Urs et al., 2019). These results are consistent with either the direct conversion of fibrinogen or action on the terminal components of coagulation, but they do not establish a single coagulation factor as the target. Also, PTLPs may exhibit broader catalytic profiles than human thrombin, including simultaneous clot formation and degradation activities, which can influence clot stability and fibrinolysis (Selvaraja and Zulfigar, 2025; Sreedhar and Tiku, 2018). Therefore, their potential clinical application requires rigorous phytochemical standardization, precise kinetic characterization, and safety assessment, including immunogenicity and therapeutic margins, to ensure reproducibility and translational feasibility.

Additionally, the contemporary understanding of hemostasis recognizes the dynamic interaction between coagulation and inflammation, a concept known as immunothrombosis. The fibrin network not only acts as a physical barrier against blood loss but also modulates the local immune response through interactions with platelets and inflammatory cells (Chatterjee et al., 2020; Marcos-Jubilar et al., 2023). In this context, plant-derived preparations that modify clot formation could influence subsequent inflammatory events; however, these downstream effects should only be claimed after direct measurement of inflammation or wound-healing outcomes.

### 4.2 Experimental and clinical evidence across wound-healing phases

The reviewed evidence encompasses crude extracts, active fractions, latex-derived enzyme preparations, isolated metabolites, and composite formulations. Unless an isolated constituent or enzyme was functionally tested, the observed effects were attributed to the complete preparation, while phytochemicals identified through compositional analyses were considered candidate mediators rather than established causal agents. Mechanistic interpretation was therefore stratified by the tested material and by the availability of functional validation at the target- or pathway-level. Figure 3 depicts the physiological transition from hemostasis to inflammation, proliferation/angiogenesis, and remodeling, including the provisional clot and platelet-derived mediators as biological links between phases. During the inflammatory phase, an ethanol-derived active fraction of *C. odorata*, an essential-oil preparation of *Achillea millefolium* L. [Asteraceae], and *Calendula officinalis* L. [Asteraceae] extracts or fractions reduced one or more inflammatory or histological endpoints in preclinical wound models (Li et al., 2024; Ghasemi et al., 2023; Parente et al., 2012). Clinical evidence for *Camellia sinensis* (L.) Kuntze [Theaceae] in perineal wounds remains preliminary: although pain and healing scores improved over time, similar improvement was also observed in the placebo group, and clear treatment superiority was not established (Sayahi et al., 2024). Topical green-tea formulations have also reduced bleeding time *in vivo*, although these findings remain formulation-specific (Kalalinia et al., 2020). The inflammatory phase is characterized by the release of proinflammatory cytokines such as tumor necrosis factor alpha (TNF-α), interleukin-1 beta (IL-1β), interleukin-6 (IL-6), as well as by the generation of reactive oxygen species; attenuating these mediators can limit secondary tissue damage and promote a more efficient transition to the proliferative phase (Bayer, 2022). The cited effects support inflammatory modulation at the preparation level, although they do not establish that the phenols, flavonoids, or other classes of phytochemicals identified are the causal agents, unless these components have been analyzed separately.

In the proliferative phase, the fibrin-rich provisional matrix promotes cell migration, primarily of fibroblasts, keratinocytes, and endothelial cells via integrin-dependent interactions, and provides a scaffold for re-epithelialization and neovascularization (Wilkinson and Hardman, 2020). At the phytoconstituent level, notoginsenoside Ft1 from *P. notoginseng* promoted fibroblast proliferation, collagen production, wound closure, re-epithelialization, granulation tissue formation, and angiogenesis through PI3K/Akt/mTOR-associated signaling (Zhang et al., 2016). Total saponins from *P. notoginseng* also promoted endothelial cell migration and angiogenic responses through GSK-3β/β-catenin-associated signaling (Lei et al., 2022). These latter effects should be attributed to the total saponin preparation. At the preparation level, *Potentilla erecta* (L.) Raeusch. [Rosaceae] root extract and *A. millefolium* essential oil preparations increased wound contraction or closure, collagen-related outcomes, and histological repair in animal models (Kaltalioglu et al., 2020; Ghasemi et al., 2023). Fresh *Aloe vera* (L.) Burm.f. [Asphodelaceae] gel increased wound contraction and shortened epithelialization time in a rabbit excision-wound model (Al Dhamary et al., 2024), whereas the active fraction of *C. odorata* promoted wound repair in a rat skin-injury model (Li et al., 2024). These outcomes should remain attributed to the corresponding extracts, gel, or active fraction.

At the epithelial and proliferative levels, polymeric fractions of proanthocyanidins and polysaccharides derived from *Hamamelis virginiana* L. [Hamamelidaceae] bark increased keratinocyte proliferation and differentiation, while an observational pediatric study reported improvement in minor superficial skin lesions and localized inflammation with a Hamamelis ointment. These findings pertain to irritated skin and minor superficial conditions and do not constitute controlled evidence in open wounds (Deters et al., 2001; Wolff and Kieser, 2007). An aqueous ointment of *S. dulcis* leaf extract promoted gap closure in L929 fibroblasts and showed antibacterial activity *in vitro*, but no wound-healing activity was demonstrated in animals or patients (Thirumurthy et al., 2024).

In the remodeling phase, the progressive replacement of type III collagen (Col III) by type I determines the mechanical strength and final functionality of the scar tissue. Studies in animal models have shown that *Symphytum officinale* L. [Boraginaceae] extract-containing creams increased re-epithelialization and collagen deposition in rats, with the 20% formulation showing the greatest histological improvement under the shared experimental protocol (Mârza et al., 2024); while *Alchemilla vulgaris* L. [Rosaceae] extracts and gels improved fibroblast migration and recovery of experimentally irritated human skin relative to matched controls; however, collagen-fiber architecture in an open-wound model was not quantitatively assessed (Tasić-Kostov et al., 2019). The healing activity of *Urtica dioica* L. [Urticaceae] has been associated mainly with re-epithelialization, collagen deposition, and histological repair *in vivo* (Zouari Bouassida et al., 2017). *Calendula officinalis* preparations have increased collagen synthesis and accelerated wound closure in animal models, accompanied by improved mechanical strength of the forming tissue (Parente et al., 2012; Buzzi et al., 2016). Preliminary clinical evidence with *Plantago major* aqueous and ethanolic extracts suggests benefits in the repair of chronic wounds, while in hydroalcoholic-extract topical gel, *P. major* L. [Plantaginaceae] accelerated closure of diabetic foot and pressure ulcers (Ghanadian et al., 2024; Zubair et al., 2016). From a molecular perspective, remodeling involves a dynamic equilibrium between matrix metalloproteinases (MMPs) and their tissue inhibitors of metalloproteinases (TIMPs), which govern the controlled degradation and reorganization of collagen. Because direct modulation of MMPs or their TIMPs has not been directly demonstrated for the cited *S. officinale*, *A. vulgaris*, *U. dioica*, or *P. major* preparations, their potential remodeling effects should be inferred only from the reported collagen-related, histological, or wound-closure outcomes rather than as evidence of a validated MMP/TIMP mechanism (Bayer, 2022; Gill and Parks, 2008).

Antimicrobial activity is best regarded as a cross-cutting supportive function rather than a discrete wound-healing phase. Among plant-only or plant-dominant preparations, essential oil of *A. millefolium* L. [Asteraceae], extracts or fractions of *C. odorata* (L.) R.M.King & H.Rob. [Asteraceae], methanolic root extract of *P. erecta* (L.) Raeusch. [Rosaceae], and extracts of *U. dioica* L. [Urticaceae] showed antimicrobial activity of variable magnitude. Fresh inner-leaf gel of *Aloe vera* (L.) Burm.f. [Asphodelaceae] showed modest activity against *Escherichia coli* and no detectable activity against *Staphylococcus aureus*, whereas the *P. erecta* extract was less potent than the corresponding reference antimicrobials. Organism-, assay-, and concentration-specific findings are summarized in Table 1 (Li et al., 2024; Sangnim et al., 2022; Li et al., 2025; Ghasemi et al., 2023; Kalalinia et al., 2020; Asadi et al., 2013).

**TABLE 1:** Antimicrobial activity of selected plant-derived preparations and associated hemostatic or wound-healing findings.

Accepted botanical name, authority, and family	Tested preparation/material	Microorganisms, assay, and antimicrobial outcome	Associated hemostatic or wound-healing finding	References
*Achillea millefolium* L. [Asteraceae]	AMEO, purchased from Shafa Kurdistan Inc. (Kurdistan, Iran), was incorporated into Eucerin to prepare 1%, 2%, and 3% (w/w) ointments.	MIC of AMEO against *Staphylococcus aureus* and *Pseudomonas aeruginosa* were determined using a broth-dilution assay. AMEO exhibited bacteriostatic activity against both pathogens, with an MIC of 1 mg/mL for each microorganism.	AMEO 1% and 2% significantly increased wound closure, re-epithelialization, collagen deposition, angiogenesis, and overall histological repair in rats, while reducing edema and inflammatory response.	Ghasemi et al. (2023)
*Ageratum conyzoides* L. [Asteraceae]	Aerial flower and leaf parts were used to prepare AC-AgNPs and an AC-AgNP wound dressing.	*Escherichia coli*, *Klebsiella pneumoniae*, *Pseudomonas aeruginosa*, and *Staphylococcus aureus*; *in vitro* antibacterial assays. AC-AgNPs exhibited size-dependent antibacterial activity, with increasing particle size associated with higher MIC and MBC values and reduced potency, particularly against *S. aureus* (MIC: 1.25–5 μg/mL for 30–90 nm particles).	AC-AgNPs have a strong hemostatic effect, which is related to their size, concentration, and negative charge. AC-AgNPs reduce APTT and PT, increase fibrinogen, activate factor XII and platelets (increased expression of the activation marker CD62P and enhanced GPIIb/IIIa receptor-mediated platelet aggregation), and reduce fibrinolysis. *In vivo*, they decreased bleeding and accelerated wound healing more effectively than gauze and gelatin.	Li et al. (2025)
*Aloe vera* (L.) Burm.f. [Asphodelaceae]	Fresh gel obtained from the parenchymatous cells in the fresh leaves of *Aloe vera*. *Aloe vera* (AV) gel with Carbomer. AV gel with cuttlefish bone and lotus root.	Disk diffusion assay with *Escherichia coli* and *Staphylococcus aureus* in Kirby–Bauer disks containing 10 μg amoxicillin/meropenem, and including AV gel; AV gel alone showed modest activity against *E. coli* and none against *S. aureus*, whereas antibiotic-loaded AV gel produced inhibition zones of 20 ± 1.4 and 24 ± 0.7 mm, respectively. Animal study of mice skin wound models infected with bioluminescent *S. aureus*; wounds treated with the *Aloe vera* hemostatic gel containing antibiotics exhibited a more rapid reduction in bacterial quantity.	AV fresh gel, when topically administered to an open wound, induces significant wound contraction and accelerates wound healing. AV gel with Carbomer in Bleed Control Kit (MedEduQuest Co., Guangzhou, China). AV gel containing cuttlefish bone and lotus root powders effectively stopped continuous bleeding in a porcine thigh subcutaneous tissue model without additional compression; it showed suitable tissue-matching viscosity and supported coagulation and vasoconstriction.	Al Dhamary et al. (2024), Shin et al. (2024)
*Artemisia absinthium* L. [Asteraceae]	Hot methanolic leaf (HMEL) extract of *Artemisia absinthium* and an extract-derived aqueous reaction mixture (RM).	10% w/v of the RM was prepared by dissolving HMEL of *Artemisia absinthium*; susceptibility assays with RM showed more activity against *Bacillus subtilis, Streptococcus pyogenes, Escherichia coli, Klebsiella pneumoniae, Pseudomonas aeruginosa,* and *Staphylococcus aureus*. However, the spectrum of activity was significantly less than the standard ciprofloxacin disc (5 µg/disc).	RM possesses wound healing properties; improved rat wound repair and reduced IL-1β, IL-6, TNF-α, caspase-3, and caspase-9 levels.	Sultan et al. (2020)
*Camellia sinensis* (L.) Kuntze [Theaceae]	Hydroalcoholic extract of green tea isolated from *Camellia sinensis* and formulated in 2%–4% PVA formulations. Vaseline with 0.6% green tea extract.	Antimicrobial effect of green tea extract (4% v/v), PVA, and green tea/PVA 4% v/v on *Staphylococcus aureus* and *Pseudomonas aeruginosa* was evaluated by MIC assay. PVA alone showed no antibacterial activity, while, 716 μg/mL of green tea and 2860 μg/mL of green tea/PVA 4% inhibited the growth *S. aureus* and *P. aeruginosa*, respectively. Both formulations were less potent than amoxicillin against *S. aureus* (8 μg/mL) and streptomycin against *P. aeruginosa* (120 μg/mL).	In mice, both 2% and 4% formulations were able to stop hemorrhage approximately at an equal time compared with tranexamic acid (TXA) 50 mg/ml as a control, and the lowest bleeding time was 6.4 ± 0.51 s for green tea/PVA 4%. Vaseline + 0.6% green tea extract improved wound contraction and microscopic repair in rats. No inflammation, eczema and/or infection were observed.	Kalalinia et al. (2020), Asadi et al. (2013)
*Chromolaena odorata* (L.) R.M.King & H.Rob. [Asteraceae]	30% EEF obtained from the ethanol extract of leaves and stems, ethanolic extract, and extract-loaded liquid plaster.	Antimicrobial activities against Gram-positive bacteria (*Staphylococcus aureus* and *Staphylococcus epidermidis*) and Gram-negative bacteria (*Escherichia coli*, *Klebsiella pneumoniae* and *Pseudomonas aeruginosa*) were determined by dilution and agar-diffusion assays with 30% EEF. Antibacterial activity was observed against certain clinically isolated bacteria, particularly the Gram‐negative bacteria. MIC of *Chromolaena odorata* ethanolic leaf extracts inhibiting *S. epidermidis* and *S. aureus* were 0.81 and 6.25 mg/mL, respectively; and the MBC against *S. epidermidis* and *S. aureus* was 1.62 and 12.5 mg/mL, respectively. Liquid plasters containing 20–50 mg (4X–10X MBC) *Chromolaena odorata* extract inhibit growth of *S. epidermidis* and *S. aureus*.	A concentration of 1.0 g/mL of the 30% EEF accelerated cutaneous wound healing in rats on the 3rd, 6th, and 9th day post-operation, with the healing effect increasing over time. Also, 30% EEF reduced bleeding time and showed anti-inflammatory-associated findings. *Chromolaena odorata* ethanol leaf extract at the concentration range of 0.0625–1 mg/mL provided platelet coagulant activities with an average platelet coagulation size of 0.5–1.0 mm.	Li et al. (2024), Sangnim et al. (2022)
*Cichorium intybus* L. [Asteraceae]	Leaf-callus-extract-mediated AgNPs; separate seed-extract-mediated AgNPs and AuNPs.	*Staphylococcus aureus* and *Escherichia coli*; disk-diffusion assay. Callus-derived AgNPs inhibited both bacteria.	Seed-derived nanoparticle preparations accelerated wound closure in mice, with AgNPs producing the strongest response. Antimicrobial and repair results were generated with different plant-derived nanoparticle preparations.	Khalid et al. (2025), Gharari et al. (2022)
*Jatropha curcas* L. [Euphorbiaceae]	Latex solvent extract formulated as a topical spray.	J. *curcas* latex extract (butanol extract) was dissolved in ethanol and acetone as a co-solvent (80:20 v/v). Antibacterial activity was tested using diffusion and MIC/MBC assays with *Staphylococcus aureus*, *Staphylococcus epidermidis*, *Escherichia coli*, and *Pseudomonas aeruginosa*. Broad bactericidal activity was reported, with MIC and MBC of 5 mg/mL against both gram-negative and gram-positive bacteria, whereas vancomycin and gentamicin exhibited MIC at 1 and 4 μg/mL against gram-positive and gram-negative bacteria, respectively.	J. *curcas *latex extract slightly promoted collagen production (47–198 pg/mL), while J. *curcas *latex formulation prominently promoted it (179–335 pg/mL). J. *curcas* latex formulation (31 μg/mL) promoted wound healing in human keratinocyte (HaCaT) and Human fibroblast (HB) cells.	Tinpun et al. (2020)
*Potentilla erecta* (L.) Raeusch. [Rosaceae]	Methanolic root extract.	Tested microorganisms were *Staphylococcus aureus*, *Enterococcus faecalis*, *Escherichia coli*, *Pseudomonas aeruginosa*, *Klebsiella pneumoniae*, *Acinetobacter baumannii*, *Bacillus cereus*, *Bacillus subtilis subsp. spizizenii*, *Enterobacter cloacae*, *Yersinia pseudotuberculosis*, and *Candida albicans*; microdilution (20–0.039 μg/mL). MEPE showed its strongest activity against *Y. pseudotuberculosis* (MIC 156.25 μg/mL) and weaker activity against the remaining microorganisms, particularly *P. aeruginosa*, *A. baumannii*, and *C. albicans* (MIC 5000 μg/mL); it was less potent than the reference antimicrobials (ampicillin, amikacin, or fluconazole).	MEPE increased contraction and collagen content and reduced wound area and lipid peroxidation in diabetic rat wounds.	Kaltalioglu et al. (2020)
*Urtica dioica* L. [Urticaceae]	Hydroethanolic or processed aqueous leaf/aerial-part extracts and corresponding topical preparations.	*Staphylococcus aureus*, *Enterococcus faecalis*, *Pseudomonas aeruginosa*, and *Escherichia coli*; agar-diffusion and MIC assays. The strongest activity was reported against *P. aeruginosa* and *E. faecalis*.	The preparations shortened bleeding time and increased wound closure, epithelialization, collagen deposition, and hydroxyproline while reducing inflammation; effects remain attributable to the respective complete extracts/formulations.	Zouari Bouassida et al. (2017), Kasouni et al. (2021)

Antimicrobial results are assay- and preparation-specific and should not be interpreted as clinical efficacy against infected wounds. For plant-mediated nanoparticles, PVA systems, hydrogels, and other composite materials, activity is attributed to the complete formulation unless component-specific controls permit a narrower conclusion. Where antimicrobial and hemostatic/wound-healing outcomes were generated with different preparations from the same species, this is stated and does not establish a single multifunctional product. Botanical nomenclature and author abbreviations were verified against POWO, Plants of the World Online (http://www.plantsoftheworldonline.org). Abbreviations: AC-AgNPs, *Ageratum conyzoides*-mediated silver nanoparticles; AMEO, *Achillea millefolium* essential oil; AgNPs, silver nanoparticles; APTT, activated partial thromboplastin time; AuNPs, gold nanoparticles; EEF, ethanol eluate fraction; IL-1β, interleukin-1 beta; IL-6, interleukin-6; MBC, minimum bactericidal concentration; MEPE, methanolic root extract of *Potentilla erecta*; MIC, minimum inhibitory concentration; PT, prothrombin time; PVA, poly(vinyl alcohol); TNF-α, tumor necrosis factor alpha.

Engineered or multicomponent systems included *A. conyzoides* L. [Asteraceae]-mediated silver nanoparticles (Li et al., 2025), *Cichorium intybus* L. [Asteraceae]-derived silver nanoparticles (Khalid et al., 2025; Gharari et al., 2022), *C. sinensis* (L.) Kuntze [Theaceae]/PVA formulations (Kalalinia et al., 2020), and an *Aloe vera*-based composite hydrogel containing additional antibacterial agents (Shin et al., 2024). For *C. intybus*, antimicrobial and wound-closure findings were obtained with different nanoparticle preparations and should not be conflated. Because silver, carriers, and added antibacterial agents may contribute substantially, activity should be attributed to the complete formulation unless component-specific controls support a narrower assignment (Li et al., 2025; Khalid et al., 2025; Gharari et al., 2022; Kalalinia et al., 2020; Shin et al., 2024). Antimicrobial screening alone does not demonstrate reduced wound inflammation or improved tissue repair; these downstream effects require direct assessment of microbial burden and repair outcomes in an infected-wound model.

In humans, a *C. sinensis* ointment was evaluated for perineal pain and episiotomy wound healing; pain and healing scores improved over time (Sayahi et al., 2024). This study provides evidence at the human formulation level but does not establish definitive clinical efficacy or a specific anti-inflammatory or antimicrobial mechanism. Extracts of Plantago major leaves promoted repair in an *ex vivo* model, and a hydroalcoholic extract gel was evaluated as an adjunct to routine care in patients with diabetic foot or pressure ulcers (Ghanadian et al., 2024). These advances provide preliminary clinical evidence, although the open design, heterogeneity of ulcer types, and absence of a vehicle-identical control limit causal attribution to the extract. Similarly, a *V. thapsus*-based cream improved episiotomy REEDA (Redness, Edema, Ecchymosis, Discharge, Approximation) scores on day 10, with no early differences between groups. Therefore, the clinical signal should be described as modest, time-dependent, and formulation-dependent, rather than conclusive evidence of accelerated wound healing (Taleb and Saeedi, 2021).

Direct evidence at the constituent or target level is available in only a limited number of cases, whereas most anti-inflammatory, proliferative, antimicrobial, and matrix-related findings remain at the extract, fraction, enzyme, or formulation level. Therefore, the current literature supports further research into defined and standardized plant preparations but does not support a general assertion that plant metabolites, as a class, promote all phases of wound healing.

### 4.3 Plant metabolites, proteolytic enzymes, and proposed mechanisms of action: translational implications

The reported activities may arise from plant proteases and diverse phytochemical classes; however, causal attribution remains limited for most preparations. From a translational perspective, interest lies not only in their traditional use but also in identifying specific biochemical mechanisms that enable their rational integration into modern therapeutic strategies.

Direct target-level hemostatic evidence was limited to a small number of isolated metabolites or functionally characterized enzyme preparations. Among these, proteases with thrombin-like activity are among the best-characterized mechanisms at the molecular level. These enzymes directly cleave fibrinogen in plasma, generating fibrin monomers that are subsequently polymerized and stabilized by factor XIIIa. Protease-enriched latex fractions promoted fibrin formation and accelerated clotting in factor VIII–deficient plasma. These findings support activity at the level of fibrinogen conversion or terminal clot formation but do not establish an alternative physiological coagulation pathway or a specific coagulation-factor target (Urs et al., 2019). From a translational perspective, this mechanism offers potential advantages for developing topical hemostatic agents that can act relatively independently of certain plasma deficiencies, as suggested by models of partial factor VIII deficiency (Singh et al., 2021).

Further enzymatic evidence has been obtained from proteases derived from rhizomes and latex. A crude aqueous fraction of protease and CAP-II, purified from *Curcuma aromatica*, shortened the clotting time of human plasma, while CAP-II hydrolyzed fibrinogen chains and crosslinked fibrin (Shivalingu et al., 2015; Shivalingu et al., 2016). A dialyzed aqueous fraction of protease from *Curcuma longa* similarly shortened clotting time but completely hydrolyzed fibrinogen chains Aα, Bβ, and γ (Shivalingu et al., 2015). These findings support serine protease-associated activity but also reveal competitive fibrinolytic (or fibrinogen) effects that preclude interpreting these materials as uniformly prohemostatic. Purified drupin from *Ficus drupacea* promoted coagulation and platelet aggregation, reduced tail bleeding time in mice, and activated MAPK, NF-κB, PI3K/Akt, cPLA_2_, and PAR1/PAR4-associated thromboxane A_2_ signaling (Manjuprasanna et al., 2021). While drupin provides relatively strong evidence at the pathway level, its systemic safety and thrombogenic potential remain to be determined.

In addition to enzymatic mechanisms, other classes of plant metabolites contribute to hemostasis through physicochemical interactions. Tannins have a high affinity for structural and plasma proteins, forming stable complexes that contribute to their astringent effect (Adamczyk et al., 2017). In the context of topical hemostasis, this property can promote local tissue contraction and the formation of a temporary barrier that limits superficial bleeding. Furthermore, biomaterials incorporating tannic acid have demonstrated hemostatic and antioxidant properties in experimental models, supporting their potential application in advanced formulations to control external bleeding (Zhou et al., 2022). Notoginsenoside Ft1 has been shown to activate P2Y_12_-dependent platelet signaling, promoting platelet aggregation in experimental models (Gao et al., 2014). This effect suggests a possible contribution to primary hemostasis by amplifying platelet activation. However, given the complexity of platelet signaling and the potential risk of systemic thrombotic events, its eventual clinical application would require careful standardization, dose control, and thorough safety evaluation.

Additionally, clot formation not only arrests bleeding but also establishes a provisional fibrin matrix that actively regulates early inflammatory signaling by coordinating leukocyte recruitment and cytokine release, including TNF-α, IL-1β, and IL-6, which are central mediators of the inflammatory phase of wound healing (Wilkinson and Hardman, 2020; Kearney et al., 2021). NF-κB-related effects have been reported primarily for phenolic- or flavonoid-rich extracts; unless isolated metabolites were tested, these results should be attributed to the preparation rather than to the entire chemical class. In addition, phytochemical studies have reported inhibition of cyclooxygenase-2 (COX-2) and inducible nitric oxide synthase (iNOS), suggesting attenuation of prostaglandin synthesis and nitric oxide-mediated inflammatory amplification (Molefe et al., 2025). By regulating these pathways, these extract-level effects may help prevent excessive or prolonged inflammation that could impair the orderly progression of tissue repair. Polyphenols have also been shown to influence platelet-mediated inflammatory signaling, including modulation of P2Y_12_-dependent activation pathways, thereby linking hemostatic regulation with inflammatory control (Ed Nignpense et al., 2019). This interaction is consistent with the broader concept of immunothrombosis, in which platelets and fibrin participate in immune modulation and inflammatory signaling beyond their classical hemostatic role (Chatterjee et al., 2020; Marcos-Jubilar et al., 2023; De Nardi et al., 2024).

Meanwhile, the fibrin-rich clot functions as a provisional ECM that supports the migration of fibroblasts and endothelial cells through integrin-mediated interactions (Wilkinson and Hardman, 2020). Plant-derived polysaccharides and flavonoids, along with increased expression of angiogenic mediators such as VEGF reported in preclinical studies with certain plant extracts, suggest a potential pro-angiogenic effect that may improve oxygen and nutrient delivery to regenerating tissue (Lee et al., 2025). From a translational standpoint, this mechanism is particularly relevant in chronic wounds, where persistent hypoxia and impaired angiogenesis represent key barriers to effective healing.

During remodeling, the balance between MMPs and TIMPs regulates collagen reorganization and the tissue’s final mechanical strength (Wilkinson and Hardman, 2020). Relative to untreated or vehicle-treated controls, *P. erecta* root extract and *S. officinale* extract-containing creams increased collagen content or improved histological repair indices. However, collagen-fiber orientation and organization were not consistently quantified; therefore, the available evidence does not support a claim of a more organized fibrillar architecture (Kaltalioglu et al., 2020; Mârza et al., 2024). Although the direct modulation of MMPs or TIMPs by these phenolic-rich extracts has not been demonstrated, their antioxidant and anti-inflammatory effects may indirectly influence the remodeling microenvironment (Wilkinson and Hardman, 2020; Molefe et al., 2025). The plant species, preparation types, and reported constituent classes are summarized in Supplementary Table S1; potential remodeling effects therefore remain indirect and are inferred from antioxidant, anti-inflammatory, and histological findings. Target-level evidence was identified for fibrinogen-cleaving plant cysteine proteases and notoginsenoside Ft1–mediated P2Y_12_ activation, rather than for most matrix-remodeling effects (Gao et al., 2014; Rajesh et al., 2005; Urs et al., 2019). The reviewed evidence supports further investigation of defined enzymes and isolated metabolites, as well as standardized fractions and composite formulations, for hemostatic and wound-management applications. Effects reported for the *C. odorata* active fraction and the *A. conyzoides*–derived silver-nanoparticle formulation should be attributed to the tested preparations (Li et al., 2024; Li et al., 2025).

## 5 Critical integration of ethnomedicinal knowledge and scientific evidence

Ethnomedicinal use is interpreted as a source of prioritized and context-dependent research hypotheses, rather than as evidence of therapeutic efficacy in itself. The frequent use of plant components for hemorrhage control and wound care has been documented for generations in different medical traditions and around the world (Pazyar et al., 2014; Salazar-Gómez and Alonso-Castro, 2022; Molefe et al., 2025). However, scientific validation requires more than species-level agreement. It is necessary to consider the plant part used, the preparation method, the route of administration, the dosage, the experimental model, and the results obtained. Traditional poultices, decoctions, or latex applications retain relevant information about local use and preparation methods, but they cannot be assumed to reflect optimal extraction, dosage, or efficacy. The main issue is not whether a traditionally used species has demonstrated some biological activity, but whether this activity is supported by the same or comparable material and by increasingly robust functional, mechanistic, and clinical evidence.

In the reviewed literature, this agreement is strongest when the same constituent, whether isolated, in the active fraction, or in a preparation, has been evaluated for hemostatic and wound-repair effects. The notoginsenoside Ft1 from *P. notoginseng* provides a clear example at the constituent level: it enhanced platelet aggregation and reduced bleeding through P2Y12-dependent signaling, while separate studies demonstrated fibroblast proliferation, collagen production, angiogenesis, and wound repair associated with PI3K/Akt/mTOR signaling (Gao et al., 2014; Zhang et al., 2016). An active fraction derived from the ethanolic extract of *C. odorata* showed hemostatic, anti-inflammatory, antimicrobial, and wound-repairing effects in the same experimental investigation (Li et al., 2024). The hydroethanolic extract of *U. dioica* reduced bleeding time, improved wound closure, and enhanced collagen deposition, although the effect was attributed to the crude extract (Zouari Bouassida et al., 2017). The preparation of silver nanoparticles using *A. conyzoides* extract provides direct, dual evidence at the formulation level on hemostatic and anti-inflammatory properties, but the effects of the plant extract, the silver component, and the complete nanoparticle structure remain unexplained (Li et al., 2025). These examples support varying degrees of agreement and should not be considered mechanistically equivalent.

In contrast, there is apparent confirmation of traditional use that is supported only at the species level, as hemostatic and wound-healing effects have been demonstrated with different materials. Crude extracts of *T. procumbens* decreased clotting time *in vitro*, while wound-repairing effects were attributed to the isolated serine protease, procumbenase, in a separate *in vivo* study (Alshargabi et al., 2023; Gubbiveeranna et al., 2024). These results do not establish that the same preparation produces both effects. Other species have shown complex or contradictory effects. A topical formulation of *Myrtus communis* L. [Myrtaceae] leaves reduced bleeding and induced platelet aggregation, but also prolonged PT and aPTT, indicating that its overall action cannot be described solely as procoagulant (Ebrahimi et al., 2020). Evidence of wound repair was also obtained with a different berry aqueous extract-containing gel (Khodaie et al., 2021). *Aloe vera* in fresh gel form improved wound contraction in rabbits, while a lyophilized aqueous preparation prolonged the clotting time of human whole blood, and the hemostatic contribution of *Aloe* in composite hydrogels was not isolated from that of the other components of the formulation (Al Dhamary et al., 2024; Melenshia et al., 2024; Shin et al., 2024). Similarly, a combination of absorbable gelatin sponge and *C. officinalis* oil shortened clotting time compared with untreated blood but did not surpass the effect of gelatin alone, leaving the specific hemostatic contribution of the plant uncertain (Ayyanahalli Matta et al., 2022). These differences demonstrate why the association at the species level should not be interpreted as direct validation of a traditional preparation.

*Agastache rugosa* exhibits bidirectional, material-dependent activity, as the total extract and some fractions produced procoagulant detection signals, while isolated acacetin and tilianin were anticoagulant (Cao et al., 2017). *Scoparia dulcis* and *V. thapsus* showed species-level agreement with different preparations: their hemostatic and repair-related effects were not demonstrated using the same material. Therefore, these results should not be interpreted as validating a single dual-action botanical product (Ediriweera et al., 2011; Thirumurthy et al., 2024; Taleb and Saeedi, 2021; Uzuner et al., 2025).

Furthermore, it is necessary to distinguish mechanistic plausibility from demonstrated causality. Tannins can interact with and precipitate proteins, which explains the astringent or wound-shrinking effects attributed to tannin-rich preparations (Adamczyk et al., 2017; Soares et al., 2020). However, the identification of tannins, flavonoids, or other classes of phytoconstituents in an extract does not imply that they are responsible for the observed biological effect. Stronger mechanistic evidence has been reported for cysteine-protease-rich latex fractions that cleave fibrinogen and promote fibrin formation (Rajesh et al., 2005; Bindhu and Singh, 2014; Urs et al., 2019; Singh et al., 2015; Singh et al., 2021). Some of these preparations also degrade fibrinogen, fibrin, or already formed clots. *Calotropis gigantea* preparations have produced local hemorrhagic effects or tissue damage under certain administration conditions. These findings provide mechanistic support for selected latex preparations but also reveal competing clot-forming and clot-degrading activities that may compromise clot stability and therapeutic safety. Clinical benefit depends on dose, route of administration, substrate specificity, local retention, and the balance between coagulation and fibrinolysis.

In humans, the available evidence is limited and methodologically heterogeneous. At the formulation level, a randomized, double-blind, placebo-controlled trial of a *Croton lechleri* Müll.Arg. [Euphorbiaceae] resin cream reported faster healing of small cauterized wounds, providing relatively strong clinical evidence; however, these results should not be generalized to deep, infected, or chronic wounds (Namjoyan et al., 2016). A *P. major* hydroalcoholic extract gel improved healing in patients with diabetic foot or pressure ulcers, but the open-label design, heterogeneity of ulcer types, and lack of a vehicle-identical control limit the specific causal attribution of the extract (Ghanadian et al., 2024). Clinical studies of *C. officinalis* suggest benefits in venous leg ulcers, although open-label comparative designs, concomitant standard care, and formulation-specific interventions limit the certainty and generalizability of the results (Duran et al., 2005; Buzzi et al., 2016). In the trial of *C. sinensis* ointment for episiotomy wounds, pain and healing scores improved in both groups, without clear treatment superiority over time; this study should not be interpreted as definitive proof of clinical efficacy (Sayahi et al., 2024). These studies support continued clinical research but do not warrant sweeping claims that medicinal plants, as a therapeutic class, are clinically effective for wound healing.

The integration of ethnomedicinal knowledge and scientific evidence should be understood as bidirectional, but conditional. Ethnomedicine can guide the selection of candidate species, plant parts, preparation methods, routes of administration, and locally relevant outcomes. Experimental and clinical research must determine whether these observations are reproducible, mechanistically plausible, safe, and superior to appropriate controls. The traditional conception of wound care, as a process involving bleeding control, modulation of inflammation, infection prevention, and tissue repair, shows partial conceptual overlap with the hemostasis-wound healing axis associated with immunothrombosis (Kearney et al., 2021; De Nardi et al., 2024). However, the term “validation” should be reserved for cases where the traditional preparation and the experimentally tested material are comparable, the biological effects are reproducible, the attribution of components is adequately controlled, and a clinical benefit superior to that of placebo, vehicle, or routine use is demonstrated. This constitutes a critical, stratified, evidence-based approach consistent with current calls for structured research agendas in traditional, complementary, and integrative medicine (Ahn et al., 2025).

## 6 Safety profile, potential risks, and translational considerations

Medicinal plants with procoagulant and wound-healing activities represent a therapeutic resource of growing interest due to their ability to integrate bleeding control with modulation of inflammation and tissue repair. Although preclinical evidence supports their biological activity in experimental models, the systematic characterization of their safety profile remains limited. Studies focused on coagulation or wound-healing parameters predominate in the literature, whereas evaluation of therapeutic margins, subchronic toxicity, and medium- and long-term systemic effects remains underdeveloped (Molefe et al., 2025; Salazar-Gómez and Alonso-Castro, 2022; Abdallah et al., 2022; European Medicines Agency Committee on Herbal Medicinal Products, 2018). In addition, key pharmacokinetic and dose–response parameters that determine therapeutic efficacy and safety remain poorly defined.

### 6.1 Pharmacokinetic, bioavailability and exposure–response considerations

Despite the growing body of evidence supporting the procoagulant and wound-healing activity of medicinal plants, pharmacokinetic and dose–response relationships remain insufficiently characterized (Leite et al., 2021). Most available studies rely on *in vitro* assays or *in vivo* experimental models and frequently administer chemically heterogeneous extracts. Consequently, information regarding the absorption, distribution, metabolism, and elimination of their bioactive constituents or characteristic chemical markers remains scarce (Salazar-Gómez and Alonso-Castro, 2022; Dominguez More et al., 2017; Chaves de Jesus et al., 2025). These gaps limit clinical translation, since the nominal dose of a crude extract does not necessarily correspond to a reproducible systemic or local exposure to the components responsible for its hemostatic or tissue repair effects.

For orally administered preparations, many polyphenols, including flavonoids, exhibit low and variable oral bioavailability due to differences in chemical structure, absorption, food-matrix interactions, and metabolism, thereby limiting their systemic biological activity (Ciupei et al., 2024). These factors can limit the concentrations achieved in target tissues and hinder the extrapolation of *in vitro* concentrations to biologically achievable *in vivo* exposures. Furthermore, the relative contributions of parent purified enzyme/protein, fractions, metabolites, and interactions among the extract’s components are rarely established. Pharmacokinetic studies should evaluate not only the selected parent compounds but also relevant metabolites and chemically characterized and validated markers that can be used to associate the product’s composition with its biological activity.

In topical preparations, which represent the most readily applicable route for hemostatic and wound-healing interventions, local exposure is likely more relevant than conventional systemic bioavailability. Therapeutic efficacy may depend on the release of the formulation’s active components, their absorption and distribution within the injured tissue, their retention at the wound site, interactions with ECM components, local degradation, and elimination from the wound environment (Jacob et al., 2026). Since the integrity and permeability of injured tissue differ from those of intact skin, unwanted systemic absorption should also be quantified whenever biologically plausible. The compound’s solubility, molecular size, chemical stability, affinity for ECM components, and the formulation’s physicochemical properties can influence these processes.

Delivery systems, such as hydrogels, fibrin matrices, nanofibers, and other advanced biomaterials, should not be considered merely inert vehicles. These systems can modify the rate and extent of component release, local residence time, tissue absorption, onset of effect, and duration of the pharmacodynamic response (Jacob et al., 2026; Mishra et al., 2025). Therefore, pharmacokinetic and safety findings obtained with an unformulated extract should not be extrapolated to a formulated product. Each delivery system should be evaluated as a defined combination of extraction and formulation, including characterization of release kinetics, retention at the wound site, formulation stability, and potential systemic exposure. The interpretation of study results is hampered by the limited standardization of doses and exposures (Sarfraz et al., 2026). Doses are frequently expressed as concentrations or total extract mass, with insufficient information on botanical identity, plant part, extraction solvent, extraction yield, chemical marker content, application area, dosing frequency, or treatment duration. As a result, similar doses may represent distinct chemical exposures. Future studies should report the total administered extract dose and the amounts of one or more quantified chemical markers. Systemic doses should be expressed relative to body weight, while topical doses should also be reported relative to the treated wound area, along with the volume or mass of the applied formulation, the dosing interval, and cumulative exposure.

The absence of clearly defined therapeutic windows creates uncertainty regarding subtherapeutic exposure and dose-related adverse effects. In preparations with marked procoagulant activity, an excessive increase in clot formation or disruption of fibrinolytic balance represents a biologically plausible safety concern. Conversely, insufficient local exposure may fail to control bleeding or promote tissue repair. Specific evaluation of the preparation is also required for local cytotoxicity, irritation, tissue compatibility, and systemic absorption, especially when products are applied repeatedly, over large wound areas, or into highly vascularized tissue (CDER, 2016; Kolodziejczyk-Czepas and Czepas, 2023). Therefore, pharmacokinetic and pharmacodynamic assessments should be integrated to identify the minimum effective exposure, the exposure associated with maximum or plateau benefit, the duration of activity, and the local or systemic toxicity thresholds.

### 6.2 Product quality and risk-based regulatory assessment

The WHO Global Strategy for Traditional Medicine 2025–2034 provides a contemporary framework for the responsible assessment and integration of traditional, complementary, and integrative medicine (TCIM). This strategy links translation to four interdependent areas: building a robust evidence base, risk-appropriate regulation, integration into health systems, and intersectoral management. Applied to procoagulant and wound-healing preparations derived from plants, this framework implies that traditional use can provide relevant historical and contextual information, but does not replace specific evidence on the quality, safety, and efficacy of each preparation (World Health Organization, 2025).

Thus, regulatory assessment must be proportionate to the product’s risk and consider the route of administration, anticipated systemic exposure, duration of use, pharmacological potency, intended therapeutic indication, and target population. The development dossier must define the botanical identity and provenance, raw material specifications, extraction and manufacturing procedures, chemical fingerprints and quantitative markers, microbial limits, stability, batch-to-batch consistency, labeling, and a pharmacovigilance plan. Compliance with Good Manufacturing Practices remains essential to ensure reproducibility and minimize quality-related adverse events (World Health Organization, 2004; World Health Organization, 2007; World Health Organization, 2025; Center for Drug Evaluation and Research, 2016). These controls are essential for heterogeneous extracts, where component variability can alter both hemostatic efficacy and risks, including excessive clot formation, disruption of fibrinolytic balance, local cytotoxicity, irritation, or sensitization. The WHO strategy explicitly supports risk-based assessment, appropriate standards, quality controls, justified labeling, international regulatory cooperation, and post-market surveillance of TCIM products (World Health Organization, 2025). These principles complement the specific regulatory pathways of each jurisdiction. In the European Union, Directive 2004/24/EC establishes a registration procedure for traditional herbal medicinal products that meet the relevant product quality and safety requirements. Long-term medicinal use should not be interpreted as evidence of safety for a new formulation, dosage, route of administration, delivery platform, or therapeutic indication (European Parliament and Council, 2004; Committee on Herbal Medicinal Products, 2024). In the United States, botanical products developed with therapeutic claims fall under the Investigational New Drug (IND) and New Drug Application (NDA). Therefore, the FDA requires sufficient information to support human safety testing and pays particular attention to chemistry, manufacturing and controls, characterization of complex mixtures, and batch-to-batch consistency (Center for Drug Evaluation and Research, 2016).

### 6.3 Potential clinical risks, vulnerable populations, and interactions

Among the main potential risks, extracts or fractions with marked hemostatic activity, primarily those that directly convert fibrinogen to fibrin or increase platelet activation and aggregation, could increase the thrombotic risk in susceptible individuals. This concern is especially relevant in patients with cardiovascular disease or other prothrombotic conditions, as well as in those receiving concomitant anticoagulant or antiplatelet therapy. Experimental results showing thrombin-like activity or increased platelet function support the biological plausibility of this risk; however, direct clinical evidence remains limited. Therefore, the potential for hypercoagulability should be presented as a risk with mechanistic support, but not yet clinically established (Chatterjee et al., 2020; Singh et al., 2021; Urs et al., 2019; Selvaraja and Zulfigar, 2025; Leite et al., 2021).

Translation to clinical trials requires specific pharmacodynamic and interaction studies, risk-based eligibility criteria, and predefined rules for treatment monitoring and discontinuation. Safety assessments must distinguish between topical and systemic preparations. For topical products, key parameters include local cytotoxicity, irritation, sensitization, effects on re-epithelialization, wound infection, systemic absorption, and changes in coagulation parameters when exposure is plausible. For oral or systemically absorbed preparations, the assessment should consider thrombotic events, platelet and coagulation responses, fibrinolytic balance, organ toxicity, and interactions with anticoagulants, antiplatelet agents, and other therapies that affect hemostasis. These outcomes should be evaluated in relation to quantified exposure and product composition, rather than solely the nominal mass of the administered extract.

### 6.4 Pharmacovigilance, real-world evidence, and health-system integration

The WHO supports the transition from isolated efficacy studies to a lifecycle evidence model. Preclinical programs should establish exposure-response relationships, therapeutic windows, local tolerance, systemic effects on coagulation, and reversibility, while clinical studies should pre-specify and record efficacy and safety outcomes and include populations representative of the intended use. Agreements on the measures of the main outcomes would improve comparability between studies, while registration in WHO-recognized clinical trial registries would reduce selective reporting and facilitate the assessment of accumulated evidence (World Health Organization, 2025).

Pharmacovigilance systems should record the exact botanical preparation, including the plant part, extraction method, formulation, batch, dose, route of administration, duration, concomitant medications, and clinical outcome, as adverse event signals cannot be reliably interpreted when the administered product is not sufficiently characterized. These systems should also differentiate between adverse effects caused by intrinsic pharmacological activity and those resulting from contamination, adulteration, misidentification, inadequate labeling, or batch-to-batch variability. WHO specifically recommends monitoring and surveillance systems for risk management of herbal and traditional medicines, along with standardized indicators to assess access, utilization, safety, and efficacy (World Health Organization, 2004; World Health Organization, 2025). Responsible clinical implementation requires quality assurance mechanisms, continuous safety monitoring, and evaluation of patient outcomes. Healthcare professionals should be trained in the appropriate use, safety, contraindications, and interaction profile of botanical products. Patient-directed materials should clearly communicate expected benefits, uncertainties, risks, and correct use, while promotional claims should be limited to evidence-supported indications. For hemostatic products, health literacy materials should explain when urgent biomedical care is required so that self-treatment or community use does not delay life-threatening management of severe bleeding, vascular injury, infection, or complicated wounds. These elements are consistent with the WHO strategy’s emphasis on educating professionals, communicating benefits and risks, potential interactions, and preventing delays in critical biomedical care (World Health Organization, 2025).

### 6.5 Sustainability, equity, and ethical considerations

Finally, translational viability extends beyond conventional clinical and regulatory criteria. Sustainable sourcing and biodiversity protection are essential to ensure the long-term availability and reproducibility of medicinal raw materials. Variability caused by geographic origin, climate change, harvesting practices, environmental conditions, species replacement, or depletion of wild populations can affect chemical composition, biological activity, and therapeutic consistency. These traceable and sustainable supply chains should be considered part of product quality, rather than an external environmental issue (World Health Organization, 2025). Research and commercialization involving traditional medical knowledge should employ participatory, culturally appropriate, and rights-respecting approaches for Indigenous Peoples and other custodians of such knowledge. Relevant measures include prior and meaningful participation, protection against misappropriation, transparent agreements regarding intellectual contributions and associated genetic resources, and fair and equitable benefit-sharing. Equity considerations should also address affordability, availability, cultural acceptability, and the inclusion of populations that may be underrepresented in clinical research. These dimensions influence public trust, long-term access, ethical legitimacy, and the feasibility of incorporating safe and effective botanical interventions into health systems (World Health Organization, 2025).

## 7 Future directions

Future research should prioritize medicinal-plant candidates with the strongest translational potential, particularly isolated metabolites, enzymes, and chemically and biologically defined fractions that have demonstrated both hemostatic and wound-healing activities using the same material, reproducible effects against clinically relevant comparators or controls, and functional validation of a molecular target or signaling pathway. Plant-derived thrombin-like cysteine proteases and notoginsenoside Ft1 exemplify the value of this approach (Gao et al., 2014; Zhang et al., 2016). Protease-rich latex fractions can cleave fibrinogen, promote fibrin formation, and partially restore coagulation in factor VIII-deficient experimental systems, while Ft1 has been linked to P2Y12-dependent platelet activation and PI3K/Akt/mTOR-associated fibroblast and wound repair responses (Gao et al., 2014; Zhang et al., 2016; Urs et al., 2019). However, the coexistence of procoagulant, fibrinogenolytic, and fibrinolytic activities in some latex preparations demonstrates that biochemical activity cannot be automatically equated with therapeutic benefit (Rajesh et al., 2005; Singh et al., 2015; Singh et al., 2021; Selvaraja and Zulfigar, 2025). Therefore, the evaluation of candidates should be governed by predefined approval or rejection criteria, including verification of identity, reproducible potency, dose-response relationships, therapeutic margin, manufacturability, formulation stability, and sustainable access to the biological source.

Identifying causal mechanisms must go beyond conventional assessments based on clotting times, PT, or aPTT, and characterize how each candidate affects clot formation, stabilization, and resolution. Future studies on plant proteases should include enzyme kinetics, cleavage site mapping, substrate specificity against fibrinogen chains and other plasma proteins, inhibitor sensitivity, and comparison with human thrombin and plasmin. Because fibrin architecture affects clot stability, cell recruitment, infection containment, and subsequent tissue repair, studies should quantify fibrin fiber structure, clot permeability, viscoelastic properties, mechanical strength, and susceptibility to fibrinolysis (Kearney et al., 2021; Risman et al., 2023). These assays are important for preparations that simultaneously promote fibrin formation and degrade fibrinogen or existing clots. The interaction between coagulation and immune signaling should also be assessed by measuring platelet activation, leukocyte recruitment, endothelial responses, cytokine production, oxidative stress, and the inflammatory response. Such tests would clarify whether a candidate promotes physiological immunothrombosis at the wound site or, conversely, promotes persistent thromboinflammatory signaling (De Nardi et al., 2024). These evaluations should be reproduced across laboratories, independent biological batches, and experimental platforms before clinical development.

Preclinical models must more accurately reproduce the wound environments for which these interventions are designed. Studies in healthy animals may overestimate efficacy and underestimate toxicity; future research should include diabetic, infected, ischemic, elderly, anticoagulated, antiplatelet-treated, and vascularly compromised models, as well as clinically relevant models of burns, deep traumatic wounds, surgical hemorrhage, and chronic ulcers. *Ex vivo* human skin, perfused tissue systems, three-dimensional wound models, and models containing clinically relevant biofilms can improve the assessment of local retention, tissue absorption, infection control, cytocompatibility, and repair. Preclinical programs should also evaluate outcomes ranging from initial hemostasis to resolution of inflammation, re-epithelialization, angiogenesis, matrix deposition, scar organization, and recurrence, rather than relying on hemorrhage control and wound closure as isolated endpoints.

Standardization must extend beyond chemical identification or the quantification of a single marker compound. Botanical products should be defined by taxonomic identification, reference specimens, plant part, developmental stage, geographic origin, chemotype, growing and harvesting conditions, extraction procedure, yield, and storage conditions. For quality assessment, chromatographic or spectrometric fingerprints, quantitative markers, microbial limits, pesticides, heavy metals, mycotoxins, residual solvents, adulterants, and stability over the expected shelf life should be considered. Chemical similarity between batches should be supplemented with validated biological potency assays linked to biological function. For hemostatic products, these assays may include fibrinogen cleavage, thrombin generation parameters, platelet activation, clot strength, or controlled bleeding models; for wound healing products, they may include cell migration, re-epithelialization, angiogenesis, antimicrobial activity, or matrix-related outcomes. This combined chemical and functional assessment will reduce the risk of products with similar phytoconstituent profiles showing substantially different pharmacodynamic effects and will provide a more solid basis for batch release and clinical dosing (Center for Drug Evaluation and Research, 2016; World Health Organization, 2007; European Medicines Agency Committee on Herbal Medicinal Products, 2018).

Currently, the evidence is primarily preclinical and topical, so topical or locally retained formulations should be prioritized over systemic administration. Future studies should quantify clot architecture, mechanical strength, permeability, fibrinolytic susceptibility, local retention, tissue biocompatibility, infection control, and subsequent remodeling in clinically relevant models, including diabetic, infected, ischemic, aged, anticoagulated, and vascularly compromised wounds. Systemic development should proceed only after pharmacokinetic, biodistribution, immunogenicity, thrombogenicity, off-target proteolysis, and plant-drug interaction studies have established an appropriate therapeutic margin.

The regulatory strategy must be tailored to the nature and intended use of the product. Crude or standardized extracts, purified enzymes, topical medicinal products, wound dressings, hydrogels, and combination drug-device products may require different quality, preclinical, manufacturing, and clinical parameters. In the European Union, Directive 2004/24/EC, the EMA preclinical guidance, and the corresponding framework for traditional herbal medicinal products provide relevant quality and safety requirements, although the traditional route of use does not replace the need for adequate evidence when new indications, purified active substances, or innovative delivery systems are proposed (European Parliament and Council, 2004; European Medicines Agency Committee on Herbal Medicinal Products, 2018; Committee on Herbal Medicinal Products, 2024). Early engagement with regulatory authorities will be important to determine product classification, acceptable potency assays, sterilization and stability requirements, risk management plans, and post-authorization pharmacovigilance. In the United States, the FDA’s guidance on the development of botanical medicinal products places botanical products intended as drugs within the IND/NDA pathway and emphasizes product consistency, documentation of chemistry, manufacturing and controls, and evidence supporting safe clinical trials (Center for Drug Evaluation and Research, 2016). The WHO also emphasizes Good Manufacturing Practices (GMP) and structured safety surveillance for herbal medicinal products (World Health Organization, 2004; World Health Organization, 2007).

Clinical development must be specific to each indication. Trials of acute hemostatic interventions should assess time to hemostasis, total blood loss, rebleeding, rescue therapy, transfusion requirements, and thrombotic complications, while trials of chronic wounds should assess standardized wound area reduction, complete closure, time to closure, recurrence, infection, pain, quality of life, and scar quality. Vehicle-identical, excipient-only, device-only, and standard-treatment controls will be essential, particularly for composite hydrogels, nanoparticle systems, and multifunctional dressings. Prospective registration, adequate statistical power, long-term safety follow-up, health-economic evaluation, and inclusion of clinically diverse populations should be considered minimum requirements for confirmatory development. In line with the WHO Global Strategy for Traditional Medicine 2025–2034, translation processes must also incorporate real-world evidence, standardized monitoring indicators, participatory research, sustainable cultivation and supply, biodiversity protection, and ethical governance of traditional medical knowledge (World Health Organization, 2025). Community participation, protection against misappropriation, and the fair and equitable sharing of benefits must be integrated from the earliest stages of candidate selection, rather than addressed only after commercial development. These measures will be necessary to ensure that plant-derived hemostatic and wound-healing interventions are not only effective and reproducible, but also accessible, culturally appropriate, environmentally sustainable, and responsibly integrated into health systems.

## 8 Conclusion

Evidence indicates that several medicinal-plant preparations can influence multiple components of the hemostasis–wound-healing axis, including clot formation, inflammatory regulation, angiogenesis, re-epithelialization, and extracellular-matrix deposition and remodeling. However, the evidence remains heterogeneous and predominantly preclinical, with most effects attributable to extracts, fractions, enzyme preparations, or composite formulations rather than to validated molecular targets. Direct mechanistic validation is concentrated in fibrinogen-cleaving plant proteases and notoginsenoside Ft1; however, the concurrent procoagulant and fibrin(ogen)olytic activities of some protease preparations preclude assuming a net therapeutic benefit. Translation should prioritize standardized topical or locally retained products supported by reproducible potency assays, exposure–response and safety characterization, indication-specific clinical trials, and lifecycle oversight. Regulatory planning, sustainable sourcing, affordability, community participation, and equitable benefit-sharing should be integrated from the outset. Traditional knowledge can guide candidate selection and hypothesis generation, but neither traditional use nor species-level concordance validates a specific preparation.
