# Supplementary material for: Plant-derived modulators of the hemostasis-wound healing axis: mechanisms, evidence, and translational potential
Source: Front Pharmacol. 2026 Sep 29;17:1902225. doi: 10.3389/fphar.2026.1902225 (PMC13659225; doi:10.3389/fphar.2026.1902225)
Supplement: Supplementary file 2 [file DataSheet2.docx]

**Supplementary Table S2.** Study-level characteristics, exposure, models, mechanistic evidence, and critical appraisal of primary publications retained for the narrative synthesis.

| **Reference** | **Accepted botanical name and authority** (Validated via POWO) | **Tested preparation/material** | **Dose/ concentration; frequency; duration** | **Experimental model and evidence level** | **Comparator/ reference condition** | **Relevant outcomes** | **Direct mechanistic evidence** | **Permitted attribution** | **Critical interpretation** |
| --- | --- | --- | --- | --- | --- | --- | --- | --- | --- |
| (Hadavi-Siahboomi et al., 2022) | *Achillea millefolium L.* [Asteraceae] | Aerial-part 80% hydroalcoholic extract and hydrodistilled essential oil. | Dose/concentration: NR. Frequency: NR; treatment/follow-up duration: NR. | Animal in vivo: An acetic acid-induced rat colitis model. | Normal and colitis controls, dexamethasone, and mesalazine. | Higher doses reduced ulceration, histological injury, colon weight/length ratio, and lipid peroxidation; myeloperoxidase decreased mainly with the hydroalcoholic extract. | Macroscopic, histological, oxidative-stress, and inflammatory-marker evidence; no causal constituent or molecular target was tested. | Effects attributable separately to the orally administered hydroalcoholic extract or essential oil. | Supports repair of injured colonic mucosa, not cutaneous wound healing; short-term, dose-dependent preclinical evidence. |
| (Ghasemi et al., 2023) | *Achillea millefolium L.* [Asteraceae] | Essential oil and oil-based topical preparation. | Dose/concentration: NR. Frequency: NR; treatment/follow-up duration: NR. | Animal in vivo + In vitro: Antibacterial assays and rat wounds. | Untreated/vehicle wounds and assay-specific antibacterial controls. | Antibacterial activity, enhanced contraction and re-epithelialization, increased collagen, and reduced inflammation. | Essential-oil composition was characterized; no constituent was causally validated. | Effects attributable to the essential oil or its topical preparation. | Supports formulation-level wound repair with antibacterial activity, but not a specific molecular mechanism. |
| (Cao et al., 2017) | *Agastache rugosa* (Fisch. & C.A.Mey.) Kuntze [Lamiaceae] | Aerial-part 70% ethanolic extract, petroleum ether, ethyl acetate and n-butanol fractions, and isolated acacetin and tilianin. | Dose/concentration: NR. Frequency: NR; treatment/follow-up duration: NR. | In vitro: Rabbit-plasma coagulation assays. | Blank plasma, vitamin K1, and breviscapine reference conditions. | The total extract shortened PT and increased fibrinogen; fractions variably shortened PT/aPTT, whereas acacetin and tilianin showed anticoagulant effects. | Coagulation-assay and chemical-isolation evidence; the active procoagulant constituent was not identified. | Procoagulant effects attributable to the total extract or active fractions, not to acacetin or tilianin. | Bidirectional in vitro evidence; no bleeding model or net hemostatic efficacy. |
| (Li et al., 2025) | *Ageratum conyzoides* L. [Asteraceae] | Aqueous-herb-extract-mediated AgNPs. | Dose/concentration: NR. Frequency: NR; treatment/follow-up duration: NR. | Animal in vivo + In vitro: Blood/plasma coagulation, platelet activation, hemocompatibility, antibacterial assays, mouse tail- and liver-bleeding models, and full-thickness skin wounds. | Plant extract, chemically synthesized AgNPs, PBS, kaolin, collagen, tirofiban, protamine sulfate, gauze, and gelatin, according to the assay. | Shortened clotting and bleeding times, reduced blood loss, increased fibrinogen and platelet activation, showed antibacterial activity and low toxicity, and accelerated wound closure. | Size-, concentration-, and charge-dependent effects; FXII/prekallikrein and platelet activation were experimentally supported. Computational analyses were noncausal. | Effects attributable to the complete AC-AgNP formulation; plant, silver, and individual-constituent contributions cannot be fully separated. | Direct preclinical hemostatic and wound-closure evidence. Anti-inflammatory activity was not directly re-established, and clinical efficacy remains untested. |
| (Kantesaria et al., 2026) | *Ageratum conyzoides* L. [Asteraceae] | Ageratum conyzoides extract loaded into a citric-acid-crosslinked poly(vinyl alcohol) hydrogel (ACE-CA/PVA). | Extract loading, antimicrobial concentrations, exposure duration, and release-test schedule: NR in the information available for unambiguous extraction. | In vitro biomaterial characterization, pH-responsive swelling/release, antibacterial assays, and L929 fibroblast cytocompatibility testing. | Blank CA/PVA hydrogel and assay-specific controls. | The extract-loaded hydrogel showed improved antibacterial activity against Staphylococcus aureus, Escherichia coli, and Aspergillus niger, together with acceptable fibroblast compatibility. | Release and material-performance data support formulation function; no causal antimicrobial constituent or wound-repair pathway was validated. | Effects attributable to the complete ACE-CA/PVA hydrogel. | The material is a wound-dressing candidate, not direct evidence of wound closure or hemostatic efficacy; no animal wound or bleeding model was reported. |
| (Shrivastava et al., 2007) | *Alchemilla vulgaris* L. [Rosaceae] | Aerial-part preparation at 0.1-1% in epithelial and myofibroblast cultures and a 3% glycerine-based preparation. | 0.1–1% in cell cultures; 3% glycerine-based preparation in rat wounds. Frequency and duration: NR. | Animal in vivo + In vitro: Rat excision wounds. | Untreated cultures/wounds and glycerine vehicle. | Increased epithelial and myofibroblast proliferation and accelerated reduction of wound diameter, with closure approximately one day earlier than vehicle. | Cell-proliferation and wound-size evidence; no molecular target was evaluated. | Effects attributable to the plant preparation, while acknowledging activity of the glycerine vehicle. | Supports preclinical repair, but the small experiment and active vehicle limit the estimated plant-specific effect. |
| (Tasić-Kostov et al., 2019) | *Alchemilla vulgaris* L. [Rosaceae] | Aqueous, ethanolic, and propylene-glycol aerial-part extracts. | Dose/concentration: NR. Frequency: N/A (single experimental exposure unless otherwise stated); assay duration: NR. | Human skin-barrier/irritation model + In vitro: Experimentally irritated human skin. | Untreated cultures, hydrogel vehicle, and matched irritated skin sites. | Increased fibroblast migration and gap closure; hydrogels supported recovery of transepidermal water loss, hydration, erythema, and skin pH. | Extract profiling and antioxidant activity; no causal compound or molecular target demonstrated. | Effects attributable to the tested extracts and corresponding hydrogels. | Supports in vitro repair and human skin-barrier recovery. The human model involved irritated skin rather than an open wound and should not be interpreted as clinical wound-healing evidence. |
| (Jettanacheawchankit et al., 2009) | *Aloe vera* (L.) Burm.f. [Asphodelaceae] | Acemannan from inner-leaf gel. | Acemannan in 0.5–2% Carbopol gels. Frequency and treatment duration: NR. | Animal in vivo + In vitro: Gingival fibroblasts and 0.5–2% Carbopol gels in rats. | Medium without acemannan; saline, triamcinolone, Carbopol, and acemannan–Carbopol formulations. | Increased fibroblast proliferation and KGF-1, VEGF, collagen I; 0.5% improved wound closure and tissue organization. | Cellular and histological evidence; growth factors increased but no causal pathway validation. | Effects attributable to acemannan; in vivo benefit incremental over Carbopol vehicle. | Supports acemannan in oral wound repair; limited by dose response, active vehicle, late histology, and oral model. |
| (Teplicki et al., 2018) | *Aloe vera* (L.) Burm.f. [Asphodelaceae] | Commercial 90% gel preparation. | Commercial gel tested at 1–3%. Single in vitro exposure; duration: NR. | In vitro: Human fibroblasts and keratinocytes. | Media with matched preservatives but no Aloe vera. | Increased fibroblast proliferation/migration and keratinocyte proliferation, migration, and viability. | Functional assays only; no constituent, ECM, or signaling pathway analysis. | Effects attributable to full commercial preparation vs preservative controls. | In vitro evidence for proliferative/migratory effects; limited by preservative toxicity and lack of physiological context. |
| (Al Dhamary et al., 2024) | *Aloe vera* (L.) Burm.f. [Asphodelaceae] | Fresh inner-leaf gel. | Dose/concentration: NR. Frequency: NR; treatment/follow-up duration: NR. | Animal in vivo: Rabbit cutaneous-wound models. | Untreated wounds, petroleum-jelly placebo, and standard topical treatment. | Increased wound contraction and shortened epithelialization time. | No histological, cellular, or molecular analysis. | Effect attributable to fresh Aloe vera gel. | Limited pilot evidence; the model was an excision rather than an incision wound. |
| (Melenshia et al., 2024) | *Aloe vera* (L.) Burm.f. [Asphodelaceae] | Reconstituted, lyophilized cold aqueous inner-leaf-gel extract; the primary report described the material as Aloe vera chinensis. | Reconstituted extract added in vitro to fresh human whole blood; single modified Lee–White assay; exact final concentration: NR; duration: until clot formation. | Ex vivo/in vitro pilot: whole-blood clotting samples from six healthy adults (three women and three men). | Paired vehicle/control whole-blood samples. | Mean clotting time increased from 10.67 ± 0.98 min in controls to 18.67 ± 1.47 min with the extract. | No platelet, coagulation-factor, fibrinogen, or molecular-target analysis was performed. | The anticoagulant effect is attributable only to the tested reconstituted lyophilized extract. | Small pilot study showing prolonged, not shortened, clotting. The finding contradicts a simple prohemostatic interpretation and requires confirmation with standardized material and coagulation assays. |
| (Shin et al., 2024) | *Aloe vera* (L.) Burm.f. [Asphodelaceae] | Aloe-gel hydrogels optimized with xanthan gum, carrageenan, Carbomer, or alginate and subsequently combined with lotus-root powder, cuttlefish-bone powder, and antimicrobial agents. | Aloe:thickener ratios from 99:1 to 70:30; selected composite ratio Aloe:thickener:lotus-root:cuttlefish-bone = 88:2:8:2. One-time simulated hemostasis/coagulation tests; antibiotic-loaded gel applied daily for 3 days in the infection model. | Simulated bleeding-control model; fresh-animal-blood clotting assay; ex vivo pig artery/skin vasoconstriction testing; mouse superficial skin-infection model. | Aloe/thickener variants, additive-specific controls, DMSO vehicle, untreated/infected conditions, and antimicrobial-agent controls as applicable. | The composite gel physically controlled simulated bleeding; cuttlefish-bone powder accelerated blood coagulation; lotus-root/tannin preparations reduced vessel diameter; antimicrobial-loaded variants reduced experimental infection. | Component pretests supported physical occlusion, calcium-rich coagulation support, and tannin-associated vasoconstriction, but the contribution of Aloe itself was not isolated. | Hemostatic and antimicrobial effects must be attributed to the complete composite formulation or to separately tested additives, not to Aloe vera alone. | Exploratory biomaterial study with heterogeneous components and nonstandardized models. It does not establish Aloe-specific hemostatic efficacy or direct wound-healing benefit. |
| (Julien et al., 2023) | *Annona senegalensis* Pers. [Annonaceae] | Hydroethanolic leaf extract tested at 1 mg/mL in human plasma exposed to Bitis rhinoceros venom. | 1 mg/mL extract; single in vitro plasma assay; assay duration: NR. | In vitro: Human plasma exposed to Bitis rhinoceros venom. | Normal plasma, venom-only plasma, and extract-plus-venom conditions. | At 0.01 mg/mL, the venom reduced the prothrombin rate by approximately half and prolonged coagulation time without significantly altering fibrinogen. The 1 mg/mL extract inhibited these venom-induced changes. | Coagulation-marker and phytochemical-screening evidence; no venom enzyme, coagulation factor, or molecular target was identified. | Protective effect attributable to the complete hydroethanolic leaf extract. | Context-dependent antivenom evidence in vitro; it does not demonstrate direct procoagulant activity in normal plasma. |
| (Boudjelal et al., 2020) | *Artemisia absinthium* L. [Asteraceae] | Methanolic extract of flowering aerial parts formulated as 5% and 10% ointments. | 5% and 10% ointments. Application frequency and treatment duration: NR. | Animal in vivo + In vitro: Rat excision wounds, with antibacterial and dermal-safety assays. | Untreated/vehicle wounds, Cicatryl-Bio reference treatment, and assay-specific antibacterial controls. | The ointments increased wound contraction, collagen deposition, granulation, and re-epithelialization; the 10% formulation performed best. Antibacterial activity and no acute dermal irritation were reported. | Histological, phytochemical, antioxidant, and antibacterial support; no constituent was causally validated. | Effects attributable to the complete methanolic extract ointments. | Formulation-specific preclinical evidence; identified phenolics and terpenoids remain candidate, not proven, mediators. |
| (Sultan et al., 2020) | *Artemisia absinthium* L. [Asteraceae] | A 10% aqueous reaction mixture prepared from hot methanolic leaf extract. | 10% leaf-extract reaction mixture. Frequency and treatment duration: NR. | Animal in vivo + In vitro: Rat excision-wound model. | Untreated wounds, povidone-iodine cream, methanol control, and ciprofloxacin for antibacterial assays. | Improved macroscopic wound repair, antibacterial activity, and reduced IL-1β, IL-6, TNF-α, caspase-3, and caspase-9 levels. | Inflammatory and apoptosis-related biomarkers were measured, but their causal involvement was not tested. | Effects attributable to the complete leaf-extract reaction mixture. | Supports antibacterial and biomarker-associated wound repair in vivo. GC-MS identification does not establish individual compounds as active mediators. |
| (Duran et al., 2005) | *Calendula officinalis* L. [Asteraceae] | Marigold-extract ointment. | Dose/concentration: NR. Frequency: NR; treatment/follow-up duration: NR. | Human clinical/observational: Patients with venous leg ulcers. | Conventional local ulcer care. | Greater reduction in ulcer surface area and enhanced epithelialization. | No direct mechanistic evidence. | Clinical effect attributable to the ointment within the complete ulcer-care regimen. | Provides human wound-healing evidence, but the open comparative design and concomitant care limit causal certainty. |
| (Parente et al., 2012) | *Calendula officinalis* L. [Asteraceae] | Flower extract and solvent fractions in antimicrobial, angiogenesis, and rat wound-healing models. | Dose/concentration: NR. Frequency: NR; treatment/follow-up duration: NR. | Animal in vivo: Rat wound-healing models. | Vehicle/untreated wounds and assay-specific reference controls. | Antibacterial, anti-inflammatory, angiogenic, fibroplastic, and wound-closure effects. | Fraction-level and histological evidence; no individual causal constituent or molecular target. | Effects attributable to the tested extract or corresponding fraction. | Supports preclinical wound repair and fraction activity, but mechanistic attribution should not exceed fraction level. |
| (Buzzi et al., 2016) | *Calendula officinalis* L. [Asteraceae] | Standardized Calendula extract product in venous leg ulcers. | Dose/concentration: NR. Frequency: NR; treatment/follow-up duration: NR. | Human clinical/observational: Venous leg ulcers. | Conventional wound care/comparator treatment. | Faster ulcer-area reduction and increased complete epithelialization. | No direct mechanistic evidence. | Effect attributable to the standardized topical product plus background care. | Clinically relevant but formulation-specific evidence. |
| (Ayyanahalli Matta et al., 2022) | *Calendula officinalis* L. [Asteraceae] | Absorbable gelatin sponge loaded with 7% Calendula officinalis oil. | 7% oil-loaded sponge; single in vitro Lee–White clotting assay; duration: until clot formation. Drug content: 56.28 ± 13.84%. | In vitro biomaterial study: MTT cytocompatibility, Franz-cell release/solid-state characterization, and human-blood clotting assay. | Absorbable gelatin sponge alone and assay control conditions. | The loaded sponge was cytocompatible and produced a small decrease in clotting time | Material compatibility and clotting time were measured; no coagulation factor, platelet pathway, or plant constituent was tested. | Any effect is attributable to the complete Calendula-oil/gelatin-sponge combination. | The primary publication reports internally inconsistent group labels for the clotting-time values between the abstract and Table 2. Regardless of this discrepancy, the difference between the absorbable gelatin sponge alone and the Calendula-oil-loaded sponge was negligible, and a plant-specific hemostatic advantage was not demonstrated. Moreover, no in vivo bleeding or wound-healing model was evaluated. |
| (Rajesh et al., 2005) | *Calotropis gigantea* (L.) W.T.Aiton [Apocynaceae] | Crude latex protein extract. | Dose/concentration: NR. Frequency: N/A (single experimental exposure unless otherwise stated); assay duration: NR. | In vitro: Plasma-clotting, fibrinogenolysis, fibrinolysis, and local hemorrhage assays. | Thrombin or reference proteases, vehicle controls, and protease inhibitors. | Promoted coagulation while also degrading fibrinogen and fibrin clots; local hemorrhagic activity was detected. | Proteolytic activity was abolished by iodoacetic acid, supporting cysteine-protease involvement. | Dual procoagulant and fibrin(ogen)olytic effects attributable to the crude latex cysteine-protease pool. | Demonstrates competing clot-forming and clot-degrading activities. Local hemorrhagic effects represent a major safety limitation. |
| (Bindhu and Singh, 2014) | *Calotropis gigantea* (L.) W.T.Aiton [Apocynaceae] | Ammonium-sulfate-precipitated and dialyzed latex enzyme fraction in fibrinogen-polymerization, fibrinogen-agarose, and blood-clot-lysis assays. | Dose/concentration: NR. Frequency: N/A (single experimental exposure unless otherwise stated); assay duration: NR. | In vitro: Fibrinogen-polymerization, fibrinogen-agarose, and blood-clot-lysis assays. | Buffer controls, standard proteases, and class-specific protease inhibitors. | Induced fibrinogen polymerization and also hydrolyzed fibrinogen and blood clots. | Inhibitor studies and zymography supported cysteine proteases as the active enzyme class. | Effects attributable to the partially purified latex protease fraction. | Confirms dual hemostatic and clot-lytic properties in vitro; it does not establish net hemostatic efficacy or safety in a wound model. |
| (Urs et al., 2019) | *Calotropis gigantea* (L.) W.T.Aiton [Apocynaceae] | Protease-enriched latex fraction. | Dose/concentration: NR. Frequency: NR; treatment/follow-up duration: NR. | Animal in vivo + In vitro: Fibrinogen/plasma assays, factor VIII-deficient plasma, and mouse tail-bleeding models. | Saline, aspirin-treated animals, and cysteine-protease inhibition conditions. | Formed fibrin, accelerated factor VIII-deficient plasma clotting, and reduced bleeding in normal and aspirin-treated mice. | Direct fibrinogen conversion and cysteine-protease predominance were demonstrated. | Effect attributable to the latex protease fraction, not to a single purified enzyme. | Provides direct in vivo hemostatic evidence, but intradermal or intramuscular administration produced hemorrhage and tissue damage, restricting translational potential. |
| (Asadi et al., 2013) | *Camellia sinensis* (L.) Kuntze [Theaceae] | Green-tea leaf 70% ethanolic extract formulated as a 0.6% Vaseline ointment. | 0.6% w/w topical ointment, applied daily for 21 days; wounds measured every other day; histology on days 3, 12, and 21. | Animal in vivo: surgical skin wounds in 36 male Wistar rats; preclinical wound-healing evidence. | Vaseline vehicle and normal saline. | Shortened mean healing duration and improved microscopic wound-repair indices relative to controls. | Phenolic/flavonoid content and antioxidant activity were characterized; no constituent or repair pathway was causally validated. | Effects attributable to the complete 0.6% green-tea extract ointment. | Animal evidence only. The study does not establish an antimicrobial mechanism or permit attribution to catechins or another individual metabolite. |
| (Hashemipour et al., 2017) | *Camellia sinensis* (L.) Kuntze [Theaceae] | Green-tea extract incorporated into a mucosal patch. | Dose/concentration: NR. Frequency: NR; treatment/follow-up duration: NR. | Animal in vivo: Rat palatal ulcers. | Blank patch and parallel Myrtus communis and Zataria multiflora preparations. | The green-tea preparation increased epithelial thickness on days 6 and 8, but did not show clear superiority in overall ulcer-size reduction. | Histological evidence only; no active constituent or molecular target was tested. | Effect attributable to the complete green-tea mucosal preparation. | Supports preclinical oral-mucosal epithelial repair, but clinical wound-size improvement was limited and inferior to the strongest comparator. |
| (Kalalinia et al., 2020) | *Camellia sinensis* (L.) Kuntze [Theaceae] | Hydroalcoholic green-tea leaf extract and 2-4% extract-loaded PVA formulations. | 2–4% extract-loaded PVA formulations. Antibacterial and bleeding assays: single-assay frequency; exposure/treatment duration: NR. | Animal in vivo + In vitro: Antibacterial assays and mouse tail-bleeding models. | Untreated/vehicle conditions, PVA alone, and assay-specific reference controls. | The extract and PVA formulations shortened bleeding time; the 4% formulation showed the strongest effect and antibacterial activity was also observed. | Functional bleeding evidence without platelet-, coagulation-factor-, or pathway-level validation. | Effect attributable to the complete extract/PVA formulation; PVA contributed to hemostasis. | Supports topical hemostasis in mice, but the plant-specific contribution and active catechin or tannin were not isolated. |
| (Sayahi et al., 2024) | *Camellia sinensis* (L.) Kuntze [Theaceae] | Green-tea extract ointment. | 10% green-tea ointment; application frequency: NR; assessments on days 7, 10, and 14. | Human clinical/observational: Episiotomy trial. | Identical placebo ointment. | REEDA scores were lower with the ointment on day 14, but not on days 7 or 10. Repeated-measures analysis showed a time effect without significant group or group-by-time effects. | No direct mechanistic evidence. | Any effect is attributable only to the tested ointment, not to individual catechins. | Human clinical evidence with a small, time-point-specific benefit; the absence of longitudinal treatment superiority precludes definitive efficacy. |
| (Ghalandari et al., 2017) | *Capsella bursa-pastoris* (L.) Medik. [Brassicaceae] | Hydroalcoholic extract administered as sublingual drops immediately after placental delivery. | Single sublingual administration immediately after placental delivery; extract concentration/dose and follow-up duration: NR. | Human clinical/observational: Women receiving oxytocin. | Placebo drops plus the same oxytocin regimen in a single-blind randomized trial. | Adjunctive drops reduced early postpartum blood loss more than oxytocin plus placebo. | Clinical blood-loss and hematologic outcomes only; no direct mechanistic measurements. | Effect attributable to the sublingual preparation as an adjunct to oxytocin. | Promising clinical evidence, but it does not establish stand-alone efficacy, optimal dosing, or a coagulation mechanism. |
| (Naafe et al., 2018) | *Capsella bursa-pastoris* (L.) Medik. [Brassicaceae] | Oral *C. bursa-pastoris* capsules added to mefenamic acid. | Oral capsules as adjunct to mefenamic acid over two menstrual cycles; capsule dose/frequency: NR. | Human clinical/observational: Women with heavy menstrual bleeding. | Placebo capsules plus mefenamic acid in a triple-blind randomized trial. | Adjunctive treatment produced a greater reduction in pictorial blood-loss scores over two menstrual cycles. | Clinical bleeding outcomes only; no coagulation, platelet, or molecular mechanism was assessed. | Effect attributable to the adjunctive capsule regimen with background mefenamic acid. | Short-term clinical evidence; efficacy and safety of the plant as a stand-alone intervention remain uncertain. |
| (Öztürk et al., 2022) | *Capsella bursa-pastoris* (L.) Medik. [Brassicaceae] | Topical extract mixture of Inula viscosa and C. bursa-pastoris in a rat liver-resection bleeding model. | Dose/concentration: NR. Frequency: NR; treatment/follow-up duration: NR. | Animal in vivo: Rat liver-resection bleeding model. | Absorbable sponge and Ankaferd Blood Stopper groups. | The mixture produced less postoperative hemoglobin loss and lower inflammation and necrosis than comparator treatments. | Functional blood-loss and histological evidence; no component-specific or molecular analysis. | Effect attributable only to the complete two-plant mixture. | Supports topical hemostasis in vivo, but the independent contribution of C. bursa-pastoris cannot be determined. |
| (Urs et al., 2019) | *Carica papaya* L. [Caricaceae] | Latex protease fraction and purified papain. | Dose/concentration: NR. Frequency: NR; treatment/follow-up duration: NR. | Animal in vivo + In vitro: Coagulation assays, factor VIII-deficient plasma, and mouse tail bleeding. | Saline, aspirin-treated mice, and iodoacetic-acid-treated papain or fraction. | Strong fibrin formation and bleeding reduction; papain restored factor VIII-deficient plasma clotting toward normal and remained effective in aspirin-treated mice. | Papain activity was abolished by cysteine-protease inhibition, directly confirming enzyme-dependent activity. | Effects attributable separately to papain and to the broader C. papaya latex fraction. | The strongest compound-level evidence among the latex preparations, although efficacy remains limited to biochemical and topical murine models. |
| (Soib et al., 2020) | *Carica papaya* L. [Caricaceae] | Methanolic leaf extracts prepared by reflux, ultrasound, or maceration. | Dose/concentration: NR. Frequency: NR; treatment/follow-up duration: NR. | In vitro: Human skin fibroblasts. | Untreated cells, concentration series, and extraction-method comparisons. | Selected extracts increased fibroblast proliferation and migration; the reflux extract also increased collagen synthesis. | Cell proliferation, scratch migration, and collagen assays with chemical profiling; no causal compound or pathway was established. | Effects attributable to the respective methanolic leaf extracts. | Provides in vitro repair support only; no animal wound closure, histology, or clinical validation. |
| (Jayasinghe et al., 2022) | *Carica papaya* L. [Caricaceae] | Mature leaf juice. | Dose/concentration: NR. Frequency: NR; treatment/follow-up duration: NR. | Animal in vivo + In vitro: Hydroxyurea-induced thrombocytopenic rats, with platelet, coagulation, and clot-lysis assays. | Normal and thrombocytopenic controls and dose-matched treatment groups. | Increased platelet counts, modulated thrombopoietin/IL-6/PAF-related responses, protected platelet membranes, shortened clotting time, and produced dose-dependent clot lysis. | Platelet, cytokine, membrane-integrity, coagulation, and fibrinolysis measurements; no causal constituent was identified. | Effects attributable to mature leaf juice. | Shows a bidirectional procoagulant and fibrinolytic profile in animal/ex vivo models, not direct bleeding-control efficacy. |
| (Vijayakumar et al., 2024) | *Carica papaya* L. [Caricaceae] | Commercial topical leaf-extract solution. | Dose/concentration: NR. Frequency: NR; treatment/follow-up duration: NR. | Human clinical/observational: Split-mouth study of post-extraction sockets in 48 patients. | Contralateral sockets treated with hemocoagulase. | The leaf-extract-treated sockets showed improved clinical healing scores during follow-up. | Clinical wound-score evidence only; no histological or molecular mechanism was evaluated. | Effect attributable to the tested commercial topical preparation. | Preliminary human evidence; fixed side allocation, limited blinding, and an active comparator restrict causal certainty. |
| (Pandith et al., 2012) | *Chromolaena odorata* (L.) R.M.King & H.Rob. [Asteraceae] | Lyophilized aqueous fresh-leaf extract and 50–95% ethanolic fresh- or dried-leaf extracts. | Dose/concentration: NR. Frequency: NR; treatment/follow-up duration: NR. | Animal in vivo + In vitro: Rat footpad bleeding and sheep-plasma platelet, PT, and aPTT assays. | Normal saline and 70% ethanol in vivo; normal saline and 1% Tween 80 in vitro; ADP in platelet-aggregation assays. | All extracts shortened rat bleeding time; the 70% ethanolic dried-leaf extract was most active. None induced platelet aggregation; PT was prolonged and no aPTT clot formed within four minutes. | Direct functional testing excluded enhanced platelet aggregation and plasma coagulation under the in vitro conditions; no molecular target was identified. | In vivo effects attributable to the respective complete leaf extracts, not to an isolated constituent. | Demonstrates hemostatic activity in vivo but discordant in vitro findings. The mechanism may involve local vascular or tissue effects, but this was not directly tested. |
| (Sangnim et al., 2022) | *Chromolaena odorata* (L.) R.M.King & H.Rob. [Asteraceae] | 95% ethanolic fresh-leaf extract and an optimized extract-loaded liquid-plaster formulation. | Leaves extracted for 24 h; extract tested at 0.0625–1 mg/mL in clotting assays and 15.625–1000 μg/mL for 24 h in MRC-5 cells; optimized liquid plaster contained 20 mg/mL extract. MIC/MBC: 0.25/0.5 mg/mL. | In vitro clotting, antibacterial, antioxidant, film-property, release, and MRC-5 cytocompatibility assays; limited skin-tolerability evaluation. | Unloaded liquid plaster/vehicle conditions, untreated cells, and assay-specific antibacterial controls including clindamycin. | The extract and liquid plaster showed procoagulant activity, antibacterial activity against Staphylococcus aureus and S. epidermidis, antioxidant activity, and acceptable film/cytocompatibility properties. | HPLC identified gallic acid, quercetin, and apigenin as markers; no marker or pathway was causally linked to the biological effects. | Effects attributable separately to the complete ethanolic extract or the complete extract-loaded liquid plaster. | No open-wound animal or clinical healing model was included; the formulation supports wound-management potential but does not directly demonstrate tissue repair. |
| (Abdul Latif et al., 2024) | *Chromolaena odorata* (L.) R.M.King & H.Rob. [Asteraceae] | C. odorata extract incorporated into a layered nitrile-rubber transdermal patch. | Dose/concentration: NR. Frequency: NR; treatment/follow-up duration: NR. | Animal in vivo: Rat excision wounds. | Untreated wounds, unloaded patch, and Solcoseryl-containing patch. | The plant-loaded patch increased wound contraction, protein, hexosamine and uronic acid content, collagen deposition, and granulation. | Biochemical and histological repair evidence; no molecular target was tested. | Effect attributable to the complete extract-loaded rubber patch. | Formulation-level wound-repair evidence; the extract effect cannot be separated fully from patch-mediated delivery. |
| (Li et al., 2024) | *Chromolaena odorata* (L.) R.M.King & H.Rob. [Asteraceae] | Leaves and stems. | 30%, 60%, and 90% ethanol-eluted fractions; selected 30% fraction tested at up to 1.0 g/mL in the wound study. Frequency and duration: NR. | Animal in vivo + In vitro: Rat skin wounds, dermal safety tests, and antibacterial assays. | Fraction-to-fraction screening, untreated/vehicle controls, and module-specific reference groups. | The 30% fraction most strongly reduced rabbit bleeding time and, at 1.0 g/mL, accelerated rat wound repair. Mild anti-inflammatory and antibacterial activity and no dermal irritation or sensitization were observed. | Fraction-level chemical and functional evidence; no causal constituent, coagulation target, or repair pathway was demonstrated. | Effects attributable to the complete 30% ethanol-eluted fraction. | Direct preclinical hemostatic and wound-repair evidence. In vitro procoagulant activity was weak or not clearly established; safety findings are limited to local animal models. |
| (Süntar et al., 2012) | *Cichorium intybus* L. [Asteraceae] | Methanolic extracts of aerial parts, leaves, and roots. | Dose/concentration: NR. Frequency: N/A (single experimental exposure unless otherwise stated); assay duration: NR. | Animal in vivo + In vitro: Rodent wound models. | Vehicle/untreated wounds, reference ointment, and extract/fraction/compound comparisons. | Root extract, active fractions, and β-sitosterol improved contraction, tensile strength, hydroxyproline, and histological repair, with anti-inflammatory and antioxidant activity. | Bioassay-guided isolation and direct testing support β-sitosterol as an active constituent; no molecular target was validated. | Compound-level attribution is permitted for isolated β-sitosterol and preparation-level attribution for the extracts/fractions. | Strong preclinical constituent-level evidence, but β-sitosterol may not account for all effects of the crude extracts. |
| (Gharari et al., 2022) | *Cichorium intybus* L. [Asteraceae] | Silver nanoparticles synthesized using a leaf-derived Cichorium intybus callus extract (Ci-AgNPs). | Antibacterial test concentration and exposure duration: NR; MDA-MB-231 IC50 at 48 h: 187.6 μg/mL (oncology assay, not a wound endpoint). | In vitro material characterization; disk-diffusion antibacterial assays against Staphylococcus aureus and Escherichia coli; antioxidant, erythrocyte-compatibility, and breast-cancer-cell assays. | Assay-specific controls; exact antibacterial reference conditions: NR. | Ci-AgNPs produced antibacterial inhibition zones, antioxidant activity, and negligible hemolytic/cytotoxic effects toward human erythrocytes. | Physicochemical characterization and phytochemical-content assays were performed; no wound-repair or hemostatic target was examined. | Effects attributable to the complete callus-extract-mediated silver-nanoparticle construct. | This is supportive antimicrobial biomaterial evidence only. No hemostatic assay or wound-healing model was included, so tissue-repair efficacy cannot be inferred. |
| (Khalid et al., 2025) | *Cichorium intybus* L. [Asteraceae] | Aqueous seed extract and plant-mediated silver and gold nanoparticles. | Dose/concentration: NR. Frequency: NR; treatment/follow-up duration: NR. | Animal in vivo: Wound model in albino mice. | Untreated wounds, seed extract, commercial Polyfax ointment, and comparison between AgNPs and AuNPs. | Nanoparticle preparations accelerated wound closure; AgNPs produced the strongest response. | Nanoparticle physicochemical characterization; no specific cellular or molecular target demonstrated. | Effect attributable to the complete seed-extract-metal nanoparticle constructs. | Demonstrates material-level wound healing, but the effects of the plant-derived coating cannot be separated from those of silver or gold. |
| (Vaisberg et al., 1989) | *Croton lechleri* Müll.Arg. [Euphorbiaceae] | Crude trunk latex/resin and isolated taspine hydrochloride. | Dose/concentration: NR. Frequency: NR; treatment/follow-up duration: NR. | In vitro: Fibroblast-migration and experimental wound-repair models. | Vehicle or untreated controls and comparison of crude latex with isolated taspine. | Enhanced fibroblast migration and increased wound-repair activity. | Taspine was isolated and linked directly to fibroblast chemotaxis. | Activity may be attributed to taspine for the isolated-compound experiments and to the resin for crude-preparation experiments. | Establishes taspine as a cicatrizant principle, but does not demonstrate that it accounts for all effects of the chemically complex latex. |
| (Namjoyan et al., 2016) | *Croton lechleri* Müll.Arg. [Euphorbiaceae] | Dragon’s-blood resin extract formulated as a topical cream and evaluated on small cautery wounds after skin-tag removal. | Dose/concentration: NR. Frequency: NR; treatment/follow-up duration: NR. | Human clinical/observational: Small cautery wounds after skin-tag removal. | Composition-matched placebo cream. | Faster wound-surface reduction and shorter healing duration. | No compound, receptor, or pathway level mechanism tested in participants. | Effect attributable to the complete Dragon’s-blood cream. | Randomized, double-blind human evidence, but formulation-specific findings from small acute wounds cannot be generalized directly to deep, infected, or chronic wounds. |
| (Rosa et al., 2026) | *Croton lechleri* Müll.Arg. [Euphorbiaceae] | Sap-based cream, alone or combined with nitroglycerin. | Dose/concentration: NR. Frequency: NR; treatment/follow-up duration: NR. | Animal in vivo: Equine metatarsal skin wounds. | Saline, two nitroglycerin doses, two *C. lechleri* doses, and a combined-treatment group. | *C. lechleri* increased collagen deposition and modulated inflammation and angiogenesis; nitroglycerin provided no clear additional benefit for wound contraction. | Wound contraction, histology, and bacterial-culture evidence; no molecular target was tested. | Effects attributable to the respective sap-based cream arms or to the complete combination. | Small equine study with multiple wounds per animal; findings are formulation- and model-specific and not directly generalizable to humans. |
| (Shivalingu et al., 2015) | *Curcuma aromatica* Salisb. [Zingiberaceae] | Dialyzed crude aqueous rhizome enzyme fraction. | Dose/concentration: NR. Frequency: N/A (single experimental exposure unless otherwise stated); assay duration: NR. | In vitro: Human-plasma clotting and fibrinogenolysis assays. | Untreated plasma, reference proteases, protease inhibitors, and parallel Curcuma fractions. | The fraction markedly shortened plasma clotting time and hydrolyzed the alpha, beta, and gamma fibrinogen chains. | Zymography and PMSF inhibition support a serine-protease-rich fraction; no individual enzyme was isolated in this study. | Effects attributable to the crude enzyme fraction. | In vitro procoagulant and fibrinogenolytic evidence only; no bleeding or wound-healing model was used. |
| (Shivalingu et al., 2016) | *Curcuma aromatica* Salisb. [Zingiberaceae] | Purified CAP-II serine protease from rhizome. | Dose/concentration: NR. Frequency: N/A (single experimental exposure unless otherwise stated); assay duration: NR. | In vitro: Human-plasma, fibrinogen, and cross-linked fibrin assays. | Crude enzyme fraction, buffer controls, and protease-inhibitor conditions. | CAP-II shortened human-plasma clotting time, hydrolyzed the Aα, Bβ, and γ fibrinogen chains, and degraded the α-polymer and γ–γ dimer of cross-linked fibrin. | Purified-protein characterization and PMSF-sensitive serine-protease activity provide direct enzyme-level evidence. | Effects attributable to purified CAP-II. | Strong biochemical attribution, but simultaneous procoagulant and fibrin(ogen)olytic activity was tested only in vitro; safety and bleeding control remain unproven. |
| (Shivalingu et al., 2015) | *Curcuma longa* L. [Zingiberaceae] | Dialyzed crude aqueous rhizome enzyme fraction. | Dose/concentration: NR. Frequency: N/A (single experimental exposure unless otherwise stated); assay duration: NR. | In vitro: Human-plasma clotting and fibrinogenolysis assays. | Untreated plasma, reference proteases, protease inhibitors, and parallel Curcuma fractions. | The fraction reduced human-plasma clotting time from 172 s in the control to 84 s and completely hydrolyzed the Aα, Bβ, and γ fibrinogen chains. | PMSF-sensitive activity supports serine-protease involvement; no individual active enzyme or coagulation target was identified. | Effect attributable to the enzyme fraction, not to curcumin or the whole rhizome. | In vitro evidence only; fibrinogen degradation complicates interpretation as a uniformly prohemostatic preparation. |
| (Manjuprasanna et al., 2021) | *Ficus drupacea* Thunb. [Moraceae] | Purified drupin, a latex cysteine protease. | Dose/concentration: NR. Frequency: NR; treatment/follow-up duration: NR. | Animal in vivo + In vitro: Platelet assays and mouse tail-bleeding models. | Vehicle/thrombin controls, protease inhibitors, PAR1/PAR4 antagonists, and signaling-pathway inhibitors. | Drupin promoted coagulation and platelet aggregation and reduced tail-bleeding time in mice. | PAR1/PAR4-dependent activation with MAPK, NF-κB, PI3K/Akt, cPLA2, and thromboxane A2 signaling was directly investigated. | Hemostatic and signaling effects attributable to purified drupin. | Provides enzyme- and pathway-level preclinical evidence; systemic safety and translational efficacy of the purified protease remain unresolved. |
| (Deters et al., 2001) | *Hamamelis virginiana* L. [Hamamelidaceae] | Bark-derived polymeric proanthocyanidin and polysaccharide fractions. | Dose/concentration: NR. Frequency: NR; treatment/follow-up duration: NR. | In vitro: Keratinocytes. | Untreated keratinocyte controls and corresponding control conditions in the SLS-irritation study. | Polymeric proanthocyanidins increased keratinocyte proliferation, affecting differentiation, reduced transepidermal water loss, erythema, and clinical irritation scores. | Fraction-level cellular and barrier-function evidence; no molecular target or causal signaling pathway was tested. | Keratinocyte effects attributable to proanthocyanidin fraction; human irritation outcomes attributable to the tested proanthocyanidin preparation. | Supports epidermal proliferation and recovery of irritated skin, but not open-wound closure. |
| (Wolff and Kieser, 2007) | *Hamamelis virginiana* L. [Hamamelidaceae] | Topical Hamamelis ointment in 231 children with minor skin injuries, diaper dermatitis, or localized skin inflammation within a 309-patient observational study. | Dose/concentration: NR. Frequency: NR; treatment/follow-up duration: NR. | Human clinical/observational: Children with minor skin injuries, diaper dermatitis, or localized skin inflammation within a 309-patient observational study. | Dexpanthenol ointment group (n = 78) and within-group baseline assessments. | Total signs-and-symptoms scores decreased significantly and clinically meaningfully in all diagnostic groups. Overall outcomes were similar to dexpanthenol, and the Hamamelis ointment was well tolerated. | Clinical effectiveness and tolerability assessments only; no cellular, histological, biochemical, or molecular mechanism was evaluated. | Clinical effects attributable to the complete Hamamelis ointment; no plant part, constituent, or molecular mediator can be isolated from this study. | Provides direct observational human evidence for minor skin conditions, not controlled evidence of deep-wound repair. |
| (Urs et al., 2019) | *Jatropha curcas* L. [Euphorbiaceae] | Protease-enriched latex fraction. | Dose/concentration: NR. Frequency: NR; treatment/follow-up duration: NR. | Animal in vivo + In vitro: Fibrinogen/plasma assays, factor VIII-deficient plasma, and mouse tail bleeding. | Saline and aspirin-treated mice. | Formed fibrin, accelerated deficient-plasma clotting, and reduced bleeding in normal and aspirin-treated mice. | Fraction activity was associated mainly with thrombin-like cysteine proteases. | Effect attributable to the complete latex protease fraction. | Provides direct functional hemostatic evidence, but no individual J. curcas enzyme, substrate-cleavage site, or specific coagulation target was established. |
| (Tinpun et al., 2020) | *Jatropha curcas* L. [Euphorbiaceae] | Latex solvent extract formulated as a topical spray. | Dose/concentration: NR. Frequency: N/A (single experimental exposure unless otherwise stated); assay duration: NR. | In vitro: Keratinocyte and human fibroblast assays. | Untreated cells, formulation vehicle, positive-control proteoglycan, and isolated curcacycline A where applicable. | The formulation was cytocompatible, antibacterial and antioxidant, promoted cell-gap closure, and increased collagen production. | Scratch, collagen, cytocompatibility, and chemical-characterization evidence; isolated curcacycline A did not reproduce all formulation effects. | Repair effects attributable to the complete latex-extract spray, not to curcacycline A alone. | Promising formulation-level evidence, but it remains exclusively in vitro and lacks an animal wound model. |
| (Ebrahimi et al., 2020) | *Myrtus communis* L. [Myrtaceae] | Aqueous leaf extract and topical gels. | Dose/concentration: NR. Frequency: NR; treatment/follow-up duration: NR. | Animal in vivo + In vitro: Coagulation assays and rat bleeding models. | Untreated/blank formulations and assay-specific controls. | Reduced topical bleeding and blood loss; platelet aggregation and protein precipitation were observed, alongside mixed plasma-coagulation effects. | Platelet aggregation and protein precipitation were directly assessed; no molecular target identified. | Hemostatic activity attributable to the leaf extract or gel tested. | Supports topical hemostasis, although prolonged plasma-clotting measures indicate a complex, potentially bidirectional profile. |
| (Khodaie et al., 2021) | *Myrtus communis* L. [Myrtaceae] | A 6% aqueous berry-extract gel. | 6% aqueous berry-extract gel. Frequency and treatment duration: NR. | Animal in vivo: Streptozotocin-induced diabetic and non-diabetic rats. | Untreated or vehicle-treated diabetic and non-diabetic wounds. | The gel accelerated early epidermal and dermal maturation, particularly on days 3 and 7; later differences diminished as wounds approached closure. | Histological maturation evidence without cellular or molecular target validation. | Effect attributable to the complete berry-extract gel. | Animal evidence suggests an early, rather than sustained, repair advantage; no hemostatic endpoint was assessed. |
| (Jafari et al., 2024) | *Myrtus communis* L. [Myrtaceae] | Methanolic leaf-extract ointment, alone or combined with silver nanoparticles. | Dose/concentration: NR. Frequency: NR; treatment/follow-up duration: NR. | Animal in vivo: MRSA-infected rat burn wounds. | Infected untreated control, extract alone, nanoparticle formulations, mupirocin, and silver sulfadiazine groups. | Reduced bacterial burden, oxidative/inflammatory markers, and wound area; improved histological repair. | Biochemical and histological markers were measured; no specific molecular target established. | Plant-only effects attributable to the extract ointment; combination effects to the complete nanoparticle formulation. | Demonstrates infected-burn repair in vivo, but silver-containing groups cannot be interpreted as evidence for the plant alone. |
| (Urs et al., 2019) | *Oxystelma esculentum* (L.f.) Sm. [Apocynaceae] | Protein-rich, protease-enriched latex fraction. | Dose/concentration: NR. Frequency: NR; treatment/follow-up duration: NR. | Animal in vivo + In vitro: Fibrinogen-clotting, normal-plasma PT/aPTT, factor VIII-deficient plasma, and mouse tail-bleeding assays in normal and aspirin-treated animals. | Untreated or saline-treated plasma and animals, normal versus factor VIII-deficient plasma, and saline- versus aspirin-treated mice. | The fraction promoted fibrin formation, shortened aPTT without reducing PT, accelerated clotting of factor VIII-deficient plasma, and reduced tail-bleeding time, blood loss, and hemoglobin loss in normal and aspirin-treated mice. | Direct fibrinogen conversion and shortened aPTT support thrombin-like procoagulant activity predominantly associated with latex cysteine proteases. | Hemostatic effects attributable to the complete O. esculentum latex protease fraction, not to an individual purified enzyme. | Provides in vitro and in vivo hemostatic evidence, but remains fraction-specific without identified enzyme, target, or clinical wound-repair validation. |
| (Gao et al., 2014) | *Panax notoginseng* (Burkill) F.H.Chen [Araliaceae] | Isolated notoginsenoside Ft1. | Dose/concentration: NR. Frequency: N/A (single experimental exposure unless otherwise stated); assay duration: NR. | In vitro: Platelet assays and bleeding models. | Vehicle and receptor/pathway-related experimental conditions. | Platelet aggregation and bleeding-related endpoints. | P2Y₁₂-dependent platelet signaling. | Hemostatic effect attributable to isolated Ft1. | Constituent- and target-level hemostatic evidence. |
| (Zhang et al., 2016) | *Panax notoginseng* (Burkill) F.H.Chen [Araliaceae] | Isolated Ft1. | Dose/concentration: NR. Frequency: NR; treatment/follow-up duration: NR. | Animal in vivo + In vitro: Human dermal fibroblasts under high-glucose conditions and diabetic mouse wounds. | Cellular control and PBS-treated wounds. | Fibroblast proliferation, collagen production, wound closure, re-epithelialization, granulation and angiogenesis. | PI3K/Akt/mTOR-related signaling and repair-associated molecular markers. | Wound-healing effect attributable to isolated Ft1. | Together with Gao et al., establishes direct dual evidence for the same isolated constituent. |
| (Men et al., 2020) | *Panax notoginseng* (Burkill) F.H.Chen [Araliaceae] | Total PNS in 3T3 fibroblast assays and at 0.1 mM in C57 mouse dorsal cutaneous wounds. | Total PNS dose series in fibroblasts; 0.1 mM in mouse wounds. Frequency and duration: NR. | Animal in vivo + In vitro: 3T3 fibroblast assays and at 0.1 mM in C57 mouse dorsal cutaneous wounds. | PNS-free control cultures and matched untreated wound controls. | Dose-dependent inhibition of fibroblast proliferation; at 0.1 mM increased NO, accelerated closure, and reduced fibrosis. | Functional, histological, NO-related, and α-SMA evidence; no signaling pathway was causally tested. | Effects attributable to the total-saponin preparation, not to Ft1 or another individual saponin. | Promotes healing with reduced fibrosis; active components and pathways remain unclear. |
| (Lei et al., 2022) | *Panax notoginseng* (Burkill) F.H.Chen [Araliaceae] | Total PNS at 200 μg/mL in high-glucose-exposed HUVECs and a PNS-loaded gelatin/Bletilla striata gelatin formulation. | PNS 200 μg/mL in HUVECs; dose in the PNS-loaded wound formulation: NR. Frequency and duration: NR. | Animal in vivo + In vitro: HUVECs and a PNS-loaded gelatin/Bletilla striata gelatin formulation in streptozotocin-diabetic rat wounds. | Normal-glucose and high-glucose cultures, PNS-treated cells, and β-catenin inhibition with ICG-001; petrolatum, gelatin/Bletilla striata gelatin, and the corresponding PNS-loaded formulation in vivo. | PNS restored endothelial proliferation, invasion, migration, angiogenesis, and NO release; reduced apoptosis and oxidative injury; and improved wound closure and matrix remodeling. | ICG-001 abolished the cellular protection and GSK-3β/β-catenin/VEGF activation, supporting pathway-level involvement. | Cellular effects attributable to the total-saponin preparation; animal wound effects attributable to the complete PNS-loaded formulation. | Supports pathway-linked repair in vitro and in vivo, but active components and formulation-specific effects remain unresolved. |
| (Zubair et al., 2016) | *Plantago major* L. [Plantaginaceae] | Water- and ethanol-based leaf extracts in an ex vivo porcine skin-wound model. | Dose/concentration: NR. Frequency: NR; treatment/follow-up duration: NR. | Ex vivo: Ex vivo porcine skin-wound model. | Untreated wound explants and extract-concentration series. | Both extracts stimulated wound repair, with the ethanolic extract showing the greater response. | No isolated constituent, signaling pathway, or molecular target evaluated. | Effect attributable to the respective leaf extracts. | Provides direct species-specific ex vivo evidence, but the model lacks systemic inflammation, perfusion, and immune recruitment. |
| (Ghanadian et al., 2024) | *Plantago major* L. [Plantaginaceae] | A 10% hydroalcoholic leaf-extract gel. | 10% hydroalcoholic leaf-extract gel. Frequency and treatment/follow-up duration: NR. | Human clinical/observational: Patients with diabetic foot or pressure ulcers. | Advanced dressing plus routine wound care without the plant gel. | Greater wound-size reduction and a higher frequency of complete healing. | No direct mechanistic measurements. | Clinical effect attributable to the extract gel as an adjunct to routine wound care. | Direct human evidence, although the open-label design, heterogeneous ulcer types, and absence of an identical vehicle control limit extract-specific attribution. |
| (Kaltalioglu et al., 2020) | *Potentilla erecta* (L.) Raeusch. [Rosaceae] | Methanolic root extract applied topically in full-thickness excisional wounds in streptozotocin-induced diabetic rats. | Dose/concentration: NR. Frequency: NR; treatment/follow-up duration: NR. | Animal in vivo + In vitro: Full-thickness excisional wounds in streptozotocin-induced diabetic rats; complementary antioxidant and antibacterial assays. | Untreated diabetic wounds and untreated non-diabetic wounds. | Increased wound contraction and collagen content; restored NOx and GSH levels and reduced lipid-peroxidation markers. Weak-to-moderate antibacterial activity was also reported. | Gallic acid, rutin, and protocatechuic acid were identified; collagen and redox-related biomarkers were quantified. No constituent or molecular target was causally validated. | Wound-repair effects attributable to the methanolic root extract. | Provides direct extract-level evidence in diabetic wounds. The short follow-up, absence of a saline-vehicle group, and lack of compound-specific intervention limit mechanistic attribution. |
| (Marcinczyk et al., 2021) | *Potentilla erecta* (L.) Raeusch. [Rosaceae] | Orally administered 80% methanolic rhizome extract. | Dose/concentration: NR. Frequency: NR; treatment/follow-up duration: NR. | Animal in vivo + In vitro: Streptozotocin-diabetic rats and mice, with platelet, thrombosis, coagulation, fibrinolysis, and vascular assays. | Non-diabetic and diabetic vehicle controls and multiple extract doses. | Effects were dose- and model-dependent: platelet inhibition and prolonged bleeding occurred in some conditions, whereas increased fibrin formation and reduced fibrinolysis occurred in others. | Direct platelet, thrombus, fibrin, fibrinolysis, and vascular measurements; no individual tannin or phenolic was causally validated. | Effects attributable to the complete methanolic rhizome extract. | A bidirectional profile precludes classification as uniformly hemostatic or antithrombotic; interpretation depends on dose and experimental model. |
| (Ediriweera et al., 2011) | *Scoparia dulcis* L. [Plantaginaceae] | Whole-plant aqueous decoction administered orally to rats. | Dose/concentration: NR. Frequency: NR; treatment/follow-up duration: NR. | Animal in vivo + In vitro: Rats; calcium-induced clotting assay in citrated goat blood. | Distilled water and vitamin K in vivo; saline in vitro. | Repeated dosing shortened rat clotting time. In vitro, the low concentration shortened clotting, whereas the high concentration prolonged it; PT, aPTT, TT, and platelet count were unchanged. | Functional clotting evidence only; no active constituent, coagulation factor, or molecular target was identified. | Effects attributable to the complete aqueous decoction. | Supports in vivo procoagulant activity but shows non-monotonic, bidirectional effects in vitro; no bleeding-control model was used. |
| (Thirumurthy et al., 2024) | *Scoparia dulcis* L. [Plantaginaceae] | Aqueous leaf-extract ointment. | Dose/concentration: NR. Frequency: N/A (single experimental exposure unless otherwise stated); assay duration: NR. | In vitro: L929 scratch and antibacterial assays. | Untreated/vehicle cultures and assay-specific controls. | Promoted concentration- and time-related fibroblast gap closure and inhibited S. aureus and Klebsiella spp. | Scratch-assay and phytochemical/GC–MS support; no constituent or repair pathway was causally tested. | Effects attributable to the complete leaf-extract ointment. | Preliminary in vitro repair evidence without animal-wound, histological, or clinical validation. |
| (Araújo et al., 2012) | *Symphytum officinale* L. [Boraginaceae] | Prepared and commercial leaf extracts in carbomer gel, glycerol–alcohol solution, and oil-in-water emulsions. | Dose/concentration: NR. Frequency: NR; treatment/follow-up duration: NR. | Animal in vivo: Rat open wounds. | Untreated wounds, matched vehicles, and 5% allantoin formulations. | Extract-containing emulsions increased collagen deposition and reduced inflammatory infiltration. | Quantitative histology and extract characterization; no constituent or molecular target was validated. | Effects attributable to the complete leaf-extract formulations. | The oil-in-water vehicle strongly influenced efficacy; results are formulation-specific and cannot be assigned to allantoin or another constituent. |
| (Mârza et al., 2024) | *Symphytum officinale* L. [Boraginaceae] | A 65% ethanolic root extract incorporated at 10% or 20% into oil-in-water creams. | 10% or 20% extract-containing creams. Frequency and treatment duration: NR. | Animal in vivo: Full-thickness rat skin wounds. | Untreated wounds, matched extract-free cream, and 10% versus 20% extract-containing creams. | Both creams accelerated closure and re-epithelialization; the 20% cream produced the fastest and most complete macroscopic and histological repair. | Histological, antioxidant, antimicrobial, and extract-characterization evidence; no constituent or repair pathway was causally validated. | Effects attributable to the complete extract-containing creams; individual contributions of allantoin and phenolic acids remain unproven. | The matched base supports a contribution from the added extract, but the findings remain formulation-specific. |
| (Singh et al., 2015) | *Tabernaemontana divaricata* (L.) R.Br. ex Roem. & Schult. [Apocynaceae] | Dialyzed crude latex enzyme extract. | Dose/concentration: NR. Frequency: N/A (single experimental exposure unless otherwise stated); assay duration: NR. | In vitro: Plasma-clotting, fibrinogen-polymerization, fibrinogen-agarose, and clot-lysis assays. | Artocarpus altilis latex extract, standard proteases, buffer controls, and protease inhibitors. | Shortened plasma-clotting time, promoted fibrin formation, and hydrolyzed blood clots. | Inhibitor studies identified cysteine proteases as major mediators. | Effects attributable to the crude latex protease preparation. | Demonstrates simultaneous procoagulant and thrombolytic properties. |
| (Urs et al., 2019) | *Tabernaemontana divaricata* (L.) R.Br. ex Roem. & Schult. [Apocynaceae] | Latex protease fraction. | Dose/concentration: NR. Frequency: NR; treatment/follow-up duration: NR. | Animal in vivo + In vitro: Fibrinogen/plasma assays, factor VIII-deficient plasma, and mouse tail bleeding. | Saline and aspirin treated mice. | Promoted fibrin formation, accelerated deficient plasma clotting, and reduced bleeding in normal mice. | Fraction-level thrombin-like activity associated mainly with cysteine proteases. | Hemostatic effect attributable to the latex protease fraction. | Unlike the other selected latex fractions, it did not significantly reduce bleeding in aspirin treated mice, suggesting dependence on an intact platelet response. |
| (Singh et al., 2021) | *Tabernaemontana divaricata* (L.) R.Br. ex Roem. & Schult. [Apocynaceae] | Purified latex cysteine protease evaluated against fibrinogen, fibrin, plasma clots, and related protein substrates. | Dose/concentration: NR. Frequency: N/A (single experimental exposure unless otherwise stated); assay duration: NR. | In vitro: Fibrinogen, fibrin, plasma clots, and related protein substrates. | Crude preparation and assay specific controls. | Fibrinogenolysis, fibrinolysis, clot formation, and clot degradation. | Direct purified enzyme biochemical evidence. | Purified cysteine protease. | Demonstrates fibrino(geno)lytic activity, not therapeutic bleeding control in vivo. |
| (Alshargabi et al., 2023) | *Tridax procumbens* L. [Asteraceae] | Ethanolic leaf extract at 50–800 μg in Lee–White whole-blood clotting assays using samples from 24 male and female volunteers with A+, A−, O+, or O− blood groups. | 50–800 μg extract per Lee–White assay; single ex vivo exposure; assay duration: until clot formation. | Ex vivo + In vitro: Whole-blood clotting assays using samples from 24 male and female volunteers with A+, A−, O+, or O− blood groups. | Untreated donor blood and comparisons across extract concentrations, blood groups, and sex. | The extract shortened clotting time in a concentration-dependent manner, with the strongest effects generally at 200–400 μg and variation among blood groups and sexes. | Functional whole-blood clotting evidence only; no platelet, coagulation-factor, active-constituent, or molecular-target analysis. | Procoagulant effect attributable to the complete ethanolic leaf extract. | Preliminary in vitro evidence limited by the small heterogeneous sample and absence of a bleeding model. |
| (Gubbiveeranna et al., 2024) | *Tridax procumbens* L. [Asteraceae] | Purified procumbenase from an aqueous plant extract. | Dose/concentration: NR. Frequency: NR; treatment/follow-up duration: NR. | Animal in vivo + In vitro: NIH 3T3 scratch assay and rat incision and full-thickness excision-wound models. | Untreated cultures; saline and povidone-iodine-treated wounds. | Increased fibroblast migration, wound tensile strength, contraction, and hydroxyproline content and shortened epithelialization time. | Procumbenase was purified and characterized as an approximately 109.73-kDa serine protease; no downstream repair pathway was causally validated. | Experimental wound-repair effects attributable to purified procumbenase, not to the crude plant extract as a whole. | The findings support enhanced repair, but do not establish that procumbenase accounts for all plant effects or confirm scar-free remodeling. |
| (Zouari Bouassida et al., 2017) | *Urtica dioica* L. [Urticaceae] | Hydroethanolic leaf extract in coagulation/bleeding tests and rat excision-wound models. | Dose/concentration: NR. Frequency: NR; treatment/follow-up duration: NR. | Animal in vivo: Rat excision-wound models. | Untreated or vehicle-treated animals and reference conditions. | Shortened bleeding time and accelerated wound closure, re-epithelialization, collagen accumulation, and tissue organization. | Extract composition and repair biomarkers were evaluated; no isolated causal constituent or target. | Hemostatic and wound-healing effects attributable to the tested leaf extract. | Provides direct dual-axis evidence in the same study, but remains at crude-extract level. |
| (Kasouni et al., 2021) | *Urtica dioica* L. [Urticaceae] | Processed aqueous aerial-part extract in HEK-293 and HaCaT cultures and as 0.20-0.40% ointments in rat full-thickness wounds. | 0.20–0.40% extract-containing ointments; in vitro concentration series: NR. Frequency and treatment duration: NR. | Animal in vivo + In vitro: HEK-293 and HaCaT cultures and as 0.20-0.40% ointments in rat full-thickness wounds. | Untreated cells/wounds and vehicle ointment. | The extract increased epithelial-cell proliferation and migration and the ointments accelerated wound closure and reduced inflammation in rats. | Cell-proliferation, migration, antioxidant/anti-inflammatory, and histological evidence; no molecular target was identified. | Effects attributable to the tested aqueous extract or corresponding ointment. | Supports preclinical cutaneous repair, but no hemostatic outcome or active constituent was demonstrated. |
| (Somavarapu et al., 2018) | *Vallaris solanacea* (Roth ex Roem. & Schult.) Kuntze [Apocynaceae] | Purified solanain; biochemical characterization. | Dose/concentration: NR. Frequency: NR; treatment/follow-up duration: NR. | In vitro: Biochemical characterization. | Crude latex protein and protease-assay controls. | Molecular mass, proteolytic activity, stability, and inhibitor sensitivity. | Direct characterization as a cysteine protease. | Purified solanain only. | No coagulation or bleeding endpoint was evaluated; hemostatic activity cannot be assigned to solanain. |
| (Urs et al., 2019) | *Vallaris solanacea* (Roth ex Roem. & Schult.) Kuntze [Apocynaceae] | Latex protease fraction. | Dose/concentration: NR. Frequency: NR; treatment/follow-up duration: NR. | Animal in vivo + In vitro: Fibrinogen, and mouse-tail bleeding models. | Saline-treated samples and animals. | Faster clotting and reduced bleeding in normal and aspirin-treated mice. | Fraction-level thrombin-like activity. | Hemostatic effects attributable to the V. solanacea latex protease fraction. | Activity cannot be attributed specifically to solanain, which was studied separately without hemostatic assays. |
| (Abdallah et al., 2022) | *Verbascum sinuatum* L. [Scrophulariaceae] | Lyophilized aqueous leaf extract at 50 mg/mL in platelet-poor human-plasma PT and aPTT assays. | 50 mg/mL; single in vitro PT/aPTT assay; assay duration: NR. | In vitro: Platelet-poor human-plasma PT and aPTT assays. | Untreated plasma and assay controls. | The extract significantly shortened aPTT without altering PT. | Coagulation-screening evidence only; no individual factor, enzyme, or molecular target was identified. | Effect attributable to the complete aqueous leaf extract. | Single-concentration in vitro evidence suggests intrinsic/common-pathway involvement but does not establish bleeding control or a specific target. |
| (Taleb and Saeedi, 2021) | *Verbascum thapsus* L. [Scrophulariaceae] | V. thapsus cream. | Topical cream applied twice daily for 10 days. | Human clinical/observational: Episiotomy wounds in 93 primiparous women. | Identical placebo cream in a randomized, double-blind controlled trial. | REEDA wound-healing scores were improved at day 10, with no significant between-group difference on days 1 or 3. | Clinical wound-score evidence only; no histological, biochemical, or molecular measurements. | Effect attributable to the tested topical cream. | Human evidence indicates a delayed, modest benefit; single-center design and short follow-up limit generalizability. |
| (Uzuner et al., 2025) | *Verbascum thapsus* L. [Scrophulariaceae] | Chitosan/gelatin cryogels containing 1-20% leaf extract in whole-blood clotting, antibacterial, hemolysis, and cytocompatibility assays. | Cryogels containing 1–20% leaf extract; 5% provided the best overall balance. Single in vitro assays; exposure duration: NR. | In vitro: Whole-blood clotting, antibacterial, hemolysis, and cytocompatibility assays. | Extract-free cryogel and extract-loading series. | Extract loading improved clotting, antibacterial activity, and material performance; the 5% cryogel provided the best overall balance with low hemolysis and cell viability above 80%. | Material, blood-clotting, and biocompatibility evidence; no plant-specific coagulation target was assessed. | Effect attributable to the complete extract-loaded chitosan/gelatin cryogel. | In vitro biomaterial evidence only; the intrinsic hemostatic contribution of chitosan/gelatin prevents attribution to V. thapsus alone. |
| (Zhou et al., 2022) | Not applicable (tannic acid; botanical source species NR) | Annealed curdlan–tannic-acid hybrid hydrogel; optimal curdlan:tannic-acid mixing weight ratio 1:1. | 1:1 formulation; hemolysis assessed at 12 h. Antibacterial/hemostatic dose, application frequency, and animal treatment duration: NR. | In vitro physicochemical, release, fibroblast-cytocompatibility, hemolysis, antioxidant, antibacterial, and hemostatic tests; animal in vivo full-thickness skin-defect model. | Curdlan-only/alternative-ratio hydrogels and assay-specific controls; exact animal comparator schedule: NR. | The 1:1 hydrogel showed favorable physicochemical properties, 3% hemolysis at 12 h, antibacterial and antioxidant activity, rapid hemostasis with approximately 0.35 g less blood loss, and accelerated full-thickness wound repair. | Temperature-dependent tannic-acid release was demonstrated; multifunctional effects remain material-level rather than species-level evidence. | Effects attributable to the complete curdlan–tannic-acid hydrogel. | Retain only as biomaterial evidence. Because the botanical source of tannic acid was not specified, the study cannot support attribution to any plant species or traditional botanical preparation. |

Notes: NR, not reported or not sufficiently specified in the primary publication or accessible bibliographic record to permit unambiguous extraction; N/A, not applicable. A single in vitro or ex vivo assay is listed as N/A for dosing frequency unless repeated exposure was explicitly reported.

Evidence level and mechanistic attribution are separate dimensions. Effects are attributed to the complete tested extract, fraction, purified material, formulation, or biomaterial unless component-specific experiments justify a narrower claim. For nanoparticles, hydrogels, patches, sponges, cryogels, and other composite systems, activity should not be assigned independently to the botanical component, metallic phase, carrier, or incorporated drug without appropriate controls.

(Zhou et al., 2022) is retained only as biomaterial evidence. Because the botanical source species of tannic acid was not reported, it does not constitute species-level botanical evidence.

*Abbreviations:* 3T3, murine fibroblast cell line; AC-AgNPs, Ageratum conyzoides-mediated silver nanoparticles; ADP, adenosine diphosphate; AgNPs, silver nanoparticles; aPTT, activated partial thromboplastin time; AuNPs, gold nanoparticles; CAP-II, Curcuma aromatica protease II; cPLA2, cytosolic phospholipase A2; Ft1, notoginsenoside Ft1; GC-MS, gas chromatography-mass spectrometry; GSH, reduced glutathione; GSK-3β/β-catenin, glycogen synthase kinase 3 beta/beta-catenin pathway; HaCaT, immortalized human keratinocytes; HEK-293, human embryonic kidney 293 cells; HUVECs, human umbilical-vein endothelial cells; ICG-001, β-catenin/CREB-binding-protein interaction inhibitor; IL, interleukin; L929, murine fibroblasts; MBC, minimum bactericidal concentration; MIC, minimum inhibitory concentration; MRSA, methicillin-resistant Staphylococcus aureus; NF-κB, nuclear factor kappa B; NO, nitric oxide; NOx, nitric oxide metabolites; P2Y12, platelet P2Y12 receptor; PAF, platelet-activating factor; PAR1/PAR4, protease-activated receptors 1 and 4; PBS, phosphate-buffered saline; PI3K/Akt/mTOR, phosphoinositide 3-kinase/protein kinase B/mechanistic target of rapamycin; PMSF, phenylmethylsulfonyl fluoride; PNS, Panax notoginseng saponins; POWO, Plants of the World Online ([**http://www.plantsoftheworldonline.org**](http://www.plantsoftheworldonline.org/)); PT, prothrombin time; PVA, poly(vinyl alcohol); REEDA, redness, edema, ecchymosis, discharge, and approximation; TNF-α, tumor necrosis factor alpha; TT, thrombin time; VEGF, vascular endothelial growth factor; α-SMA, alpha-smooth muscle actin.

**References**

Abdallah, L., Surakji, I., Qawasme, T., Ayyash, D., Shhadeh, R., Omar, G. and Barakat, A. (2022). In vitro activity of some medicinal plants on blood coagulation. *Turk J Pharm Sci,* 19**,** 330–335. <https://doi.org/10.4274/tjps.galenos.2021.14603>.

Abdul Latif, M., Mustafa, A., Keong, L. C. and Hamid, A. (2024). Chromolaena odorata layered-nitrile rubber polymer transdermal patch enhanced wound healing in vivo. *PLoS One,* 19**,** e0295381. <https://doi.org/10.1371/journal.pone.0295381>.

Al Dhamary, N., Al Adhal, A., Kadi, H. and Al Kamarany, M. A. (2024). Investigation effect of aloe vera in fresh gel form on rabbit’s model wound. *Journal of Complementary and Alternative Medical Research,* 25**,** 17–26. <https://doi.org/10.9734/jocamr/2024/v25i3522>.

Alshargabi, A., Mansour, W. and Al-Madhagi, W. M. (2023). Effect of tridax procumbens extracts on coagulation time according to gender and blood groups a and o: In vitro study. *Journal of Herbal Medicine,* 42**,** 100806. <https://doi.org/10.1016/j.hermed.2023.100806>.

Araújo, L. U., Reis, P. G., Barbosa, L. C., Saúde-Guimarães, D. A., Grabe-Guimarães, A., Mosqueira, V. C., Carneiro, C. M. and Silva-Barcellos, N. M. (2012). In vivo wound healing effects of symphytum officinale l. Leaves extract in different topical formulations. *Pharmazie,* 67**,** 355–60.

Asadi, S. Y., Parsaei, P., Karimi, M., Ezzati, S., Zamiri, A., Mohammadizadeh, F. and Rafieian-Kopaei, M. (2013). Effect of green tea (camellia sinensis) extract on healing process of surgical wounds in rat. *International Journal of Surgery,* 11**,** 332–337. <https://doi.org/10.1016/j.ijsu.2013.02.014>.

Ayyanahalli Matta, B. K., Kumar, S., Mehta, C. H., Nayak, U. Y. and Rodriguez, P. G. (2022). Comparative evaluation of the effectiveness of a combination of absorbable gelatin sponge and calendula officinalis with absorbable gelatin sponge used alone as a hemostatic agent-an in-vitro study. *Dent J (Basel),* 10**,** 76. <https://doi.org/10.3390/dj10050076>.

Bindhu, O. S. and Singh, M. K. (2014). Hemostatic, milk clotting and blood stain removal potential of cysteine proteases from calotropis gigantea (l.) r. Br. Latex. *Pharmacogn Mag,* 10**,** S350–6. <https://doi.org/10.4103/0973-1296.133294>.

Boudjelal, A., Smeriglio, A., Ginestra, G., Denaro, M. and Trombetta, D. (2020). Phytochemical profile, safety assessment and wound healing activity of artemisia absinthium l. *Plants (Basel),* 9**,** 1744. <https://doi.org/10.3390/plants9121744>.

Buzzi, M., De Freitas, F. and De Barros Winter, M. (2016). Therapeutic effectiveness of a calendula officinalis extract in venous leg ulcer healing. *J Wound Care,* 25**,** 732–739. <https://doi.org/10.12968/jowc.2016.25.12.732>.

Cao, P., Xie, P., Wang, X., Wang, J., Wei, J. and Kang, W. Y. (2017). Chemical constituents and coagulation activity of agastache rugosa. *BMC Complement Altern Med,* 17**,** 93. <https://doi.org/10.1186/s12906-017-1592-8>.

Deters, A., Dauer, A., Schnetz, E., Fartasch, M. and Hensel, A. (2001). High molecular compounds (polysaccharides and proanthocyanidins) from hamamelis virginiana bark: Influence on human skin keratinocyte proliferation and differentiation and influence on irritated skin. *Phytochemistry,* 58**,** 949–958. <https://doi.org/10.1016/S0031-9422(01)00361-2>.

Duran, V., Matic, M., Jovanovć, M., Mimica, N., Gajinov, Z., Poljacki, M. and Boza, P. (2005). Results of the clinical examination of an ointment with marigold (calendula officinalis) extract in the treatment of venous leg ulcers. *Int J Tissue React,* 27**,** 101–6.

Ebrahimi, F., Mahmoudi, J., Torbati, M., Karimi, P. and Valizadeh, H. (2020). Hemostatic activity of aqueous extract of myrtus communis l. Leaf in topical formulation: In vivo and in vitro evaluations. *J Ethnopharmacol,* 249**,** 112398. <https://doi.org/10.1016/j.jep.2019.112398>.

Ediriweera, E. R., Jayakody, J. R. and Ratnasooriya, W. D. (2011). Pro blood clotting activity of scoparia dulcis in rats. *Ayu,* 32**,** 271–4. <https://doi.org/10.4103/0974-8520.92547>.

Gao, B., Huang, L., Liu, H., Wu, H., Zhang, E., Yang, L., Wu, X. and Wang, Z. (2014). Platelet p2y₁₂ receptors are involved in the haemostatic effect of notoginsenoside ft1, a saponin isolated from panax notoginseng. *Br J Pharmacol,* 171**,** 214–23. <https://doi.org/10.1111/bph.12435>.

Ghalandari, S., Kariman, N., Sheikhan, Z., Mojab, F., Mirzaei, M. and Shahrahmani, H. (2017). Effect of hydroalcoholic extract of capsella bursa pastoris on early postpartum hemorrhage: A clinical trial study. *J Altern Complement Med,* 23**,** 794–799. <https://doi.org/10.1089/acm.2017.0095>.

Ghanadian, M., Soltani, R., Homayouni, A., Khorvash, F., Jouabadi, S. M. and Abdollahzadeh, M. (2024). The effect of plantago major hydroalcoholic extract on the healing of diabetic foot and pressure ulcers: A randomized open-label controlled clinical trial. *Int J Low Extrem Wounds,* 23**,** 475–481. <https://doi.org/10.1177/15347346211070723>.

Gharari, Z., Hanachi, P., Sadeghinia, H. and Walker, T. R. (2022). Cichorium intybus bio-callus synthesized silver nanoparticles: A promising antioxidant, antibacterial and anticancer compound. *International Journal of Pharmaceutics,* 625**,** 122062. <https://doi.org/10.1016/j.ijpharm.2022.122062>.

Ghasemi, M. R., Ranjbar, A., Tamri, P., Pourmoslemi, S., Nourian, A. and Dastan, D. (2023). In vitro antibacterial activity and wound healing effects of achillea millefolium essential oil in rat. *J Pharmacopuncture,* 26**,** 167–174. <https://doi.org/10.3831/kpi.2023.26.2.167>.

Gubbiveeranna, V., Megha, G. T., Kusuma, C. G., Ravikumar, H., Thoyajakshi, R. S., Vijayakumar, S., Mathad, S. N., Nagaraju, S., Wazzan, H., Khan, A., Alzahrani, K. A. and Malash, A. M. (2024). Effect of 'procumbenase' a serine protease from tridax procumbens aqueous extract on wound healing: A scar free healing of full thickness wounds. *Int J Biol Macromol,* 273**,** 133147. <https://doi.org/10.1016/j.ijbiomac.2024.133147>.

Hadavi-Siahboomi, M., Yegdaneh, A., Talebi, A. and Minaiyan, M. (2022). Ulcer-healing effect of hydroalcoholic extract and essential oil of achillea millefolium l. On murine model of colitis. *Int J Prev Med,* 13**,** 155. <https://doi.org/10.4103/ijpvm.ijpvm_50_22>.

Hashemipour, M. A., Lotfi, S., Torabi, M., Sharifi, F., Ansari, M., Ghassemi, A. and Sheikhshoaie, S. (2017). Evaluation of the effects of three plant species (myrtus communis l., camellia sinensis l., zataria multiflora boiss.) on the healing process of intraoral ulcers in rats. *J Dent (Shiraz),* 18**,** 127–135.

Jafari, Z., Bardania, H., Barmak, M. J., Eslami, S., Mahmoudi-Mourderaz, Y., Roustaei, N., Talebianpoor, M. S., Kokhdan, E. P. and Khoramrooz, S. S. (2024). Antimicrobial, anti-inflammatory, and wound healing properties of myrtus communis leaf methanolic extract ointment on burn wound infection induced by methicillin-resistant staphylococcus aureus in rats. *BioMed Research International,* 2024**,** 6758817. <https://doi.org/10.1155/2024/6758817>.

Jayasinghe, C. D., Ratnasooriya, W. D., Premakumara, S. and Udagama, P. V. (2022). Platelet augmentation activity of mature leaf juice of sri lankan wild type cultivar of carica papaya l: Insights into potential cellular mechanisms. *J Ethnopharmacol,* 296**,** 115511. <https://doi.org/10.1016/j.jep.2022.115511>.

Jettanacheawchankit, S., Sasithanasate, S., Sangvanich, P., Banlunara, W. and Thunyakitpisal, P. (2009). Acemannan stimulates gingival fibroblast proliferation; expressions of keratinocyte growth factor-1, vascular endothelial growth factor, and type i collagen; and wound healing. *Journal of pharmacological sciences,* 109**,** 525–531. <https://doi.org/10.1254/jphs.08204fp>.

Julien, G. K., Marcel, A. G., Antoinette, Y.-C. C. C., Hermann, A. M., Benoit, Y. D. C., Bernard, A. K., Karamoko, O., Mireille, D. and Joseph, D. A. (2023). An in-vitro study of the effect of hydroethanol extract of annona senegalensis leaves on bitis rhinoceros (viperidae) venom induced coagulopathy. *European Journal of Medicinal Plants,* 34**,** 33–41. <https://doi.org/10.9734/ejmp/2023/v34i11118>.

Kalalinia, F., Amiri, N., Mehrvarzian, N., Fazly Bazzaz, B. S., Iranshahi, M., Shahroodi, A., Arabzadeh, S., Abbaspour, M., Badiee Aaval, S. and Movaffagh, J. (2020). Topical green tea formulation with anti-hemorrhagic and antibacterial effects. *Iran J Basic Med Sci,* 23**,** 1085–1090. <https://doi.org/10.22038/ijbms.2020.41397.9782>.

Kaltalioglu, K., Balabanli, B. and Coskun-Cevher, S. (2020). Phenolic, antioxidant, antimicrobial, and in-vivo wound healing properties of potentilla erecta l. Root extract in diabetic rats. *Iran J Pharm Res,* 19**,** 264–274. <https://doi.org/10.22037/ijpr.2019.112411.13742>.

Kantesaria, R., Bagra, Y., Varma, P. and Panda, H. S. (2026). Plasticization of an ageratum conyzoides natural extract in crosslinked hydrogel: A promising material for combating wound infection. *Appl Biochem Biotechnol,* 198**,** 2750–2766. <https://doi.org/10.1007/s12010-026-05594-0>.

Kasouni, A. I., Chatzimitakos, T. G., Stalikas, C. D., Trangas, T., Papoudou-Bai, A. and Troganis, A. N. (2021). The unexplored wound healing activity of urtica dioica l. Extract: An in vitro and in vivo study. *Molecules,* 26**,** 6248. <https://doi.org/10.3390/molecules26206248>.

Khalid, A., Khurshid, S., Uroos, M., Khan, J., Sarwar, A., Ul Haq, E., Aziz, T., Alshehri, F., Al-Asmari, F., Baothman, B. K., Al-Joufi, F. A. and Alwethaynani, M. S. (2025). Green synthesis of silver and gold nanoparticles using seeds extract of cichorium intybus and their comparative analysis with commercially available ointment for wound healing activity. *J Mol Histol,* 56**,** 145. <https://doi.org/10.1007/s10735-025-10426-2>.

Khodaie, S. A., Emadi, F., Naseri, M., Kamalinejad, M., Riahi, S. M., Alijaniha, F. and Roghani, M. (2021). The effect of myrtus communis aqueous extract-containing gel on wound healing in streptozotocin-induced diabetic rats. *Curr Drug Discov Technol,* 18**,** 542–547. <https://doi.org/10.2174/1570163817666200712163956>.

Lei, T., Gao, Y., Duan, Y., Cui, C., Zhang, L. and Si, M. (2022). Panax notoginseng saponins improves healing of high glucose-induced wound through the gsk-3β/β-catenin pathway. *Environ Toxicol,* 37**,** 1867–1877. <https://doi.org/10.1002/tox.23533>.

Li, H.-F., Feng, H., Wang, Y., Pan, Z.-C., Yin, L., Qiu, H.-L., Qiao, H., Zhao, J.-Q., Xia, X.-Y., Hou, J.-C. and Wang, R.-X. (2024). Evaluation of hemostatic, anti-inflammatory, wound healing, skin irritation and allergy, and antimicrobial properties of active fraction from the ethanol extract of chromolaena odorata (l.) r.M. King & h. Rob. *Journal of Ethnopharmacology,* 331**,** 118330. <https://doi.org/10.1016/j.jep.2024.118330>.

Li, Y., Tan, Y., Zhao, H., Chen, S., Nilghaz, A., Cao, R. and Zhou, S. (2025). Green biosynthetic silver nanoparticles from ageratum conyzoides as multifunctional hemostatic agents: Combining hemostasis, antibacterial, and anti-inflammatory properties for effective wound healing. *Mater Today Bio,* 31**,** 101468. <https://doi.org/10.1016/j.mtbio.2025.101468>.

Manjuprasanna, V. N., Urs, A. P., Rudresha, G. V., Milan Gowda, M. D., Jayachandra, K., Hiremath, V., Rajaiah, R. and Vishwanath, B. S. (2021). Drupin, a thrombin-like protease prompts platelet activation and aggregation through protease-activated receptors. *J Cell Biochem,* 122**,** 870–881. <https://doi.org/10.1002/jcb.29917>.

Marcinczyk, N., Gołaszewska, A., Gromotowicz-Poplawska, A., Misztal, T., Strawa, J., Tomczyk, M., Kasacka, I. and Chabielska, E. (2021). Multidirectional effects of tormentil extract on hemostasis in experimental diabetes. *Front Pharmacol,* 12**,** 682987. <https://doi.org/10.3389/fphar.2021.682987>.

Mârza, S. M., Dăescu, A. M., Purdoiu, R. C., Dragomir, M., Tătaru, M., Melega, I., Nagy, A. L., Gal, A., Tăbăran, F., Bogdan, S., Moldovan, M., Pall, E., Munteanu, C., Magyari, K. and Papuc, I. (2024). Healing of skin wounds in rats using creams based on symphytum officinale extract. *Int J Mol Sci,* 25**,** 3099. <https://doi.org/10.3390/ijms25063099>.

Melenshia, D. S., Amirtham, S. M., Rebekah, G., Vinod, E. and Kachroo, U. (2024). Effect of reconstituted, lyophilized cold aqueous extract of aloe vera on human whole blood clotting time - a pilot study. *J Ayurveda Integr Med,* 15**,** 100887. <https://doi.org/10.1016/j.jaim.2024.100887>.

Men, S. Y., Huo, Q. L., Shi, L., Yan, Y., Yang, C. C., Yu, W. and Liu, B. Q. (2020). Panax notoginseng saponins promotes cutaneous wound healing and suppresses scar formation in mice. *J Cosmet Dermatol,* 19**,** 529–534. <https://doi.org/10.1111/jocd.13042>.

Naafe, M., Kariman, N., Keshavarz, Z., Khademi, N., Mojab, F. and Mohammadbeigi, A. (2018). Effect of hydroalcoholic extracts of capsella bursa-pastoris on heavy menstrual bleeding: A randomized clinical trial. *Journal of alternative and complementary medicine (New York, N.Y.),* 24**,** 694–700. <https://doi.org/10.1089/acm.2017.0267>.

Namjoyan, F., Kiashi, F., Moosavi, Z. B., Saffari, F. and Makhmalzadeh, B. S. (2016). Efficacy of dragon's blood cream on wound healing: A randomized, double-blind, placebo-controlled clinical trial. *J Tradit Complement Med,* 6**,** 37–40. <https://doi.org/10.1016/j.jtcme.2014.11.029>.

Öztürk, O. U., Ugur, M., Güzel, Y., Öztürk, M. A., Gürsoy, D., Doğan, S. and Temiz, M. (2022). Hemostatic effects of traditional inula viscosa and capsella bursa-pastoris plant mixture extract on rat liver parenchymal bleeding model. *Ulus Travma Acil Cerrahi Derg,* 28**,** 1059–1065. <https://doi.org/10.14744/tjtes.2022.90838>.

Pandith, H., Thongpraditchote, S., Wongkrajang, Y. and Gritsanapan, W. (2012). In vivo and in vitro hemostatic activity of chromolaena odorata leaf extract. *Pharm Biol,* 50**,** 1073–7. <https://doi.org/10.3109/13880209.2012.656849>.

Parente, L. M., Lino Júnior Rde, S., Tresvenzol, L. M., Vinaud, M. C., De Paula, J. R. and Paulo, N. M. (2012). Wound healing and anti-inflammatory effect in animal models of calendula officinalis l. Growing in brazil. *Evid Based Complement Alternat Med,* 2012**,** 375671. <https://doi.org/10.1155/2012/375671>.

Rajesh, R., Raghavendra Gowda, C. D., Nataraju, A., Dhananjaya, B. L., Kemparaju, K. and Vishwanath, B. S. (2005). Procoagulant activity of calotropis gigantea latex associated with fibrin(ogen)olytic activity. *Toxicon,* 46**,** 84–92. <https://doi.org/10.1016/j.toxicon.2005.03.012>.

Rosa, M. C. D. B., Campos, M. E. D. S. T., Albuquerque, A. S., Braz, M. S., Mapele, R. D. O., De Oliveira, M. C. G., Toma, H. S., Ferraz, P. F. P., Pereira, R. N. and Raymundo, D. L. (2026). Effect of croton lechleri sap-based topical treatment, with or without nitroglycerin, on healing of equine metatarsal skin wounds. *Veterinary and Animal Science,* 32**,** 100598. <https://doi.org/10.1016/j.vas.2026.100598>.

Sangnim, T., Meeboon, P., Phongsewalak, P., Prasongdee, P., Sriamornsak, P., Singh, I., Manmuan, S. and Huanbutta, K. (2022). Development and evaluation of liquid plaster loaded with chromolaena odorata leaf extract endowed with several beneficial properties to wound healing. *Gels (Basel, Switzerland)* 8**,** 72. <https://doi.org/10.3390/gels8020072>.

Sayahi, M., Jahangirimehr, A., Hatami Manesh, Z., Mojab, F. and Nikbina, M. (2024). The effect of camellia sinensis ointment on perineal pain and episiotomy wound healing in primiparous women: A triple-blind randomized clinical trial. *PLoS One,* 19**,** e0305048. <https://doi.org/10.1371/journal.pone.0305048>.

Shin, B., Hillyer, T. and Shin, W. S. (2024). Rational design and testing of antibacterial aloe vera hemostatic hydrogel. *Gels,* 10**,** 409. <https://doi.org/10.3390/gels10060409>.

Shivalingu, B. R., Vivek, H. K., Nafeesa, Z., Priya, B. S. and Swamy, S. N. (2015). Comparative analysis of procoagulant and fibrinogenolytic activity of crude protease fractions of turmeric species. *Journal of Ethnopharmacology,* 172**,** 261–264. <https://doi.org/10.1016/j.jep.2015.06.018>.

Shivalingu, B. R., Vivek, H. K., Priya, B. S., Soujanya, K. N. and Swamy, S. N. (2016). Purification and characterization of novel fibrin(ogen)olytic protease from curcuma aromatica salisb.: Role in hemostasis. *Phytomedicine,* 23**,** 1691–1698. <https://doi.org/10.1016/j.phymed.2016.09.007>.

Shrivastava, R., Cucuat, N. and John, G. W. (2007). Effects of alchemilla vulgaris and glycerine on epithelial and myofibroblast cell growth and cutaneous lesion healing in rats. *Phytother Res,* 21**,** 369–73. <https://doi.org/10.1002/ptr.2060>.

Singh, M. K., Rajagopalan, A., Tanimu, H. and Sukumaran, B. O. (2021). Purification, characterization and fibrino(geno)lytic activity of cysteine protease from tabernaemontana divaricata latex. *3 Biotech,* 11**,** 106. <https://doi.org/10.1007/s13205-021-02643-9>.

Singh, M. K., Usha, R., Hithayshree, K. R. and Bindhu, O. S. (2015). Hemostatic potential of latex proteases from tabernaemontana divaricata (l.) r. Br. Ex. Roem. And schult. And artocarpus altilis (parkinson ex. F.A. Zorn) forsberg. *J Thromb Thrombolysis,* 39**,** 43–9. <https://doi.org/10.1007/s11239-013-1012-y>.

Soib, H. H., Ismail, H. F., Husin, F., Abu Bakar, M. H., Yaakob, H. and Sarmidi, M. R. (2020). Bioassay-guided different extraction techniques of carica papaya (linn.) leaves on in vitro wound-healing activities. *Molecules (Basel, Switzerland),* 25**,** 517. <https://doi.org/10.3390/molecules25030517>.

Somavarapu, S., Vemula, S. and Reddy, I. B. (2018). Extraction, purification and characterization of a novel cysteine protease from the latex of plant vallaris solanacea. *Journal of Plant Biochemistry and Biotechnology,* 27**,** 186–198. <https://doi.org/10.1007/s13562-017-0429-3>.

Sultan, M. H., Zuwaiel, A. A., Moni, S. S., Alshahrani, S., Alqahtani, S. S., Madkhali, O. and Elmobark, M. E. (2020). Bioactive principles and potentiality of hot methanolic extract of the leaves from artemisia absinthium l "in vitro cytotoxicity against human mcf-7 breast cancer cells, antibacterial study and wound healing activity". *Curr Pharm Biotechnol,* 21**,** 1711–1721. <https://doi.org/10.2174/1389201021666200928150519>.

Süntar, I., Küpeli Akkol, E., Keles, H., Yesilada, E., Sarker, S. D. and Baykal, T. (2012). Comparative evaluation of traditional prescriptions from cichorium intybus l. For wound healing: Stepwise isolation of an active component by in vivo bioassay and its mode of activity. *J Ethnopharmacol,* 143**,** 299–309. <https://doi.org/10.1016/j.jep.2012.06.036>.

Taleb, S. and Saeedi, M. (2021). The effect of the verbascum thapsus on episiotomy wound healing in nulliparous women: A randomized controlled trial. *BMC Complement Med Ther,* 21**,** 166. <https://doi.org/10.1186/s12906-021-03339-6>.

Tasić-Kostov, M., Arsić, I., Pavlović, D., Stojanović, S., Najman, S., Naumović, S. and Tadić, V. (2019). Towards a modern approach to traditional use: In vitro and in vivo evaluation of alchemilla vulgaris l. Gel wound healing potential. *J Ethnopharmacol,* 238**,** 111789. <https://doi.org/10.1016/j.jep.2019.03.016>.

Teplicki, E., Ma, Q., Castillo, D. E., Zarei, M., Hustad, A. P., Chen, J. and Li, J. (2018). The effects of aloe vera on wound healing in cell proliferation, migration, and viability. *Wounds : a compendium of clinical research and practice,* 30**,** 263–268.

Thirumurthy, P., Natarajan, S., Tharumasivam, S. V., Dhailappan, A., Jayaraj, V. E. C., Doss, A. M. D. C. and Kumar, P. R. (2024). Phytochemical and biological evaluation of scoparia dulcis targeting wound healing efficacy. *African Journal of Biomedical Research,* 27**,** 184–192. <https://doi.org/10.53555/AJBR.v28i3S.7522>.

Tinpun, K., Nakpheng, T., Padmavathi, A. R. and Srichana, T. (2020). In vitro studies of jatropha curcas l. Latex spray formulation for wound healing applications. *Turk J Pharm Sci,* 17**,** 271–279. <https://doi.org/10.4274/tjps.galenos.2019.69875>.

Urs, A. P., Rudresha, G. V., Manjuprasanna, V. N., Suvilesh, K. N., Gowda, M. D. M., Yariswamy, M., Hiremath, V., Ramakrishnan, C., Savitha, M. N., Jayachandra, K., Sharanappa, P. and Vishwanath, B. S. (2019). Plant latex thrombin-like cysteine proteases alleviates bleeding by bypassing factor viii in murine model. *J Cell Biochem,* 120**,** 12843–12858. <https://doi.org/10.1002/jcb.28555>.

Uzuner, H., Yürük, A. and İșoğlu, İ. A. (2025). A potential hemostatic chitosan/gelatin cryogel impregnated with verbascum thapsus leaf extract for noncompressible hemorrhage management. *Biomedical Materials,* 20**,** 065026. <https://doi.org/10.1088/1748-605X/ae19b7>.

Vaisberg, A. J., Milla, M., Planas, M. C., Cordova, J. L., De Agusti, E. R., Ferreyra, R., Mustiga, M. C., Carlin, L. and Hammond, G. B. (1989). Taspine is the cicatrizant principle in sangre de grado extracted from croton lechleri. *Planta Med,* 55**,** 140–3. <https://doi.org/10.1055/s-2006-961907>.

Vijayakumar, G., Sundaram, G. A., Kumar, S. P., Lakshmanan, S., Krishnan, M. and Krishna, V. K. (2024). Comparison of efficacy of topical carica papaya leaf extract and hemocoagulase in postoperative wound healing after therapeutic orthodontic premolar extractions: A split mouth study. *Cureus,* 16**,** e61946. <https://doi.org/10.7759/cureus.61946>.

Wolff, H. H. and Kieser, M. (2007). Hamamelis in children with skin disorders and skin injuries: Results of an observational study. *European Journal of Pediatrics,* 166**,** 943–948. <https://doi.org/10.1007/s00431-006-0363-1>.

Zhang, E., Gao, B., Yang, L., Wu, X. and Wang, Z. (2016). Notoginsenoside ft1 promotes fibroblast proliferation via pi3k/akt/mtor signaling pathway and benefits wound healing in genetically diabetic mice. *J Pharmacol Exp Ther,* 356**,** 324–32. <https://doi.org/10.1124/jpet.115.229369>.

Zhou, Z., Xiao, J., Guan, S., Geng, Z., Zhao, R. and Gao, B. (2022). A hydrogen-bonded antibacterial curdlan-tannic acid hydrogel with an antioxidant and hemostatic function for wound healing. *Carbohydr Polym,* 285**,** 119235. <https://doi.org/10.1016/j.carbpol.2022.119235>.

Zouari Bouassida, K., Bardaa, S., Khimiri, M., Rebaii, T., Tounsi, S., Jlaiel, L. and Trigui, M. (2017). Exploring the urtica dioica leaves hemostatic and wound-healing potential. *Biomed Res Int,* 2017**,** 1047523. <https://doi.org/10.1155/2017/1047523>.

Zubair, M., Nybom, H., Lindholm, C., Brandner, J. M. and Rumpunen, K. (2016). Promotion of wound healing by plantago major l. Leaf extracts – ex-vivo experiments confirm experiences from traditional medicine. *Natural Product Research,* 30**,** 622–624. <https://doi.org/10.1080/14786419.2015.1034714>.
