# Supplementary material for: Plant-derived modulators of the hemostasis-wound healing axis: mechanisms, evidence, and translational potential
Source: Front Pharmacol. 2026 Sep 29;17:1902225. doi: 10.3389/fphar.2026.1902225 (PMC13659225; doi:10.3389/fphar.2026.1902225)
Supplement: Supplementary file 3 [file DataSheet3.docx]

**Supplementary Table S3.** Taxonomic and pharmacopoeial verification of plant species and botanical materials included in the narrative synthesis, including botanical components of multicomponent formulations.

| **Plant name as reported in the original study** | **Accepted botanical name and authority**  **(Validated via POWO)** | **Taxonomic status or nomenclatural note** | **Family** | **WFO ID** | **Botanical/ pharmacopoeial drug name** | **Plant part or botanical material reported** | **References** |
| --- | --- | --- | --- | --- | --- | --- | --- |
| *Achillea millefolium* | *Achillea millefolium* L. [Asteraceae] | Accepted name | Asteraceae | [wfo-0000042097](https://list.worldfloraonline.org/wfo-0000042097) | Millefolii herba (EMA/HMPC/376416/2019) | Essential oil and oil-based topical preparation; aerial-part 80% hydroalcoholic extract and hydrodistilled essential oil. | (Hadavi-Siahboomi et al., 2022, Ghasemi et al., 2023) |
| *Agastache rugosa* | *Agastache rugosa* (Fisch. & C.A.Mey.) Kuntze [Lamiaceae] | Accepted name | Lamiaceae | [wfo-0000906745](https://www.worldfloraonline.org/taxon/wfo-0000906745) | N/A | Aerial-part 70% ethanolic extract; petroleum-ether, ethyl-acetate, and n-butanol fractions; isolated acacetin and tilianin. | (Cao et al., 2017) |
| *Ageratum conyzoides* | *Ageratum conyzoides* L. [Asteraceae] | Accepted name | Asteraceae | [wfo-0000014938](https://www.worldfloraonline.org/taxon/wfo-0000014938) | N/A | Aqueous whole-herb extract used to mediate silver-nanoparticle synthesis; leaf extract incorporated into a citric-acid-crosslinked poly(vinyl alcohol) hydrogel (ACE-CA/PVA) | (Li et al., 2025, Kantesaria et al., 2026) |
| *Alchemilla vulgaris* | *Alchemilla vulgaris* L. [Rosaceae] | Accepted name | Rosaceae | [wfo-0001007689](https://www.worldfloraonline.org/taxon/wfo-0001007689) | N/A | Aerial-part aqueous, ethanolic, and propylene-glycol extracts and extract-loaded hydrogels; glycerine-based aerial-part preparation. | (Shrivastava et al., 2007, Tasić-Kostov et al., 2019) |
| *Aloe vera* | *Aloe vera* (L.) Burm.f. [Asphodelaceae] | Accepted name. The material evaluated by Melenshia et al. (2024) was reported as “Aloe vera chinensis”; Study-specific botanical authentication and voucher documentation were NR. | Asphodelaceae | [wfo-0000758976](https://www.worldfloraonline.org/taxon/wfo-0000758976) | N/A | Fresh inner-leaf gel; commercial gel preparation; acemannan isolated from inner-leaf gel and incorporated into Carbopol gels; reconstituted lyophilized cold aqueous inner-leaf-gel extract; and fresh mature inner-leaf gel incorporated into composite hydrogels containing Carbomer, lotus-root powder, and cuttlefish-bone powder, with antibiotic-loaded variants. | (Jettanacheawchankit et al., 2009, Teplicki et al., 2018, Al Dhamary et al., 2024, Melenshia et al., 2024, Shin et al., 2024) |
| *Annona senegalensis* | *Annona senegalensis* Pers. [Annonaceae] | Accepted name | Annonaceae | [wfo-0000537928](https://www.worldfloraonline.org/taxon/wfo-0000537928) | N/A | Hydroethanolic leaf extract. | (Julien et al., 2023) |
| *Artemisia absinthium* | *Artemisia absinthium* L. [Asteraceae] | Accepted name | Asteraceae | [wfo-0000134589](https://www.worldfloraonline.org/taxon/wfo-0000134589) | Absinthii herba (EMA/HMPC/751490/2016 Corr. 1) | Methanolic extract of flowering aerial parts formulated as ointments; hot methanolic leaf-extract reaction mixture. | (Boudjelal et al., 2020, Sultan et al., 2020) |
| *Bletilla striata* | *Bletilla striata* (Thunb.) Rchb.f. [Orchidaceae] | Accepted name | Orchidaceae | [wfo-0000346630](https://list.worldfloraonline.org/wfo-0000346630) | *Bletillae Rhizoma* [recognized herbal drug; applicability to the experimental BSGT material could not be verified because the source plant organ was not reported] | BSGT in a gelatin/BSGT formulation; source organ not specified in the primary study. | (Lei et al., 2022) |
| *Calendula officinalis* | *Calendula officinalis* L. [Asteraceae] | Accepted name | Asteraceae | [wfo-0000007455](https://www.worldfloraonline.org/taxon/wfo-0000007455) | Calendulae flos (EMA/HMPC/437450/2017) | Standardized topical Calendula extract product; flower extract and solvent fractions; marigold-extract ointment; and commercial *Calendula officinalis* oil incorporated at 7% into an absorbable gelatin sponge. The botanical source organ of the commercial oil was NR. | (Duran et al., 2005, Parente et al., 2012, Buzzi et al., 2016, Ayyanahalli Matta et al., 2022) |
| *Calotropis gigantea* | *Calotropis gigantea* (L.) W.T.Aiton [Apocynaceae] | Accepted name | Apocynaceae | [wfo-0001257181](https://www.worldfloraonline.org/taxon/wfo-0001257181) | N/A | Crude latex protein extract and partially purified/protease-enriched latex enzyme fractions. | (Rajesh et al., 2005, Bindhu and Singh, 2014, Urs et al., 2019) |
| *Camellia sinensis* | *Camellia sinensis* (L.) Kuntze [Theaceae] | Accepted name | Theaceae | [wfo-0000582676](https://www.worldfloraonline.org/taxon/wfo-0000582676) | Camelliae sinensis non fermentatum folium (EMA/HMPC/283630/2012) | Green-tea leaf material extracted with 70% ethanol and formulated as a 0.6% (w/w) Vaseline ointment; hydroalcoholic leaf extract incorporated into 2–4% PVA formulations; 10% topical ointment; and a green-tea mucosal patch. | (Asadi et al., 2013, Hashemipour et al., 2017, Kalalinia et al., 2020, Sayahi et al., 2024) |
| *Capsella bursa-pastoris* | *Capsella bursa-pastoris* (L.) Medik. [Brassicaceae] | Accepted name | Brassicaceae | [wfo-0000585589](https://www.worldfloraonline.org/taxon/wfo-0000585589) | Bursae pastoris herba (EMA/HMPC/262766/2010) | Dried aerial parts in a 1:1 (w/w) ethanolic mixture with Inula viscosa; oral capsules; hydroalcoholic extract in sublingual drops. | (Ghalandari et al., 2017, Naafe et al., 2018, Öztürk et al., 2022) |
| *Carica papaya* | *Carica papaya* L. [Caricaceae] | Accepted name | Caricaceae | [wfo-0000588009](https://www.worldfloraonline.org/taxon/wfo-0000588009) | N/A | Commercial topical leaf-extract solution; mature leaf juice; methanolic leaf extracts; latex protease fraction and purified papain. | (Urs et al., 2019, Soib et al., 2020, Jayasinghe et al., 2022, Vijayakumar et al., 2024) |
| *Chromolaena odorata* | *Chromolaena odorata* (L.) R.M.King & H.Rob. [Asteraceae] | Accepted name | Asteraceae | [wfo-0000067484](https://www.worldfloraonline.org/taxon/wfo-0000067484) | N/A | Fresh-leaf 95% ethanolic extract and an optimized extract-loaded liquid-plaster formulation; leaf extract incorporated into a nitrile-rubber patch; leaf-and-stem ethanol-eluted fractions; and aqueous and ethanolic extracts of fresh or dried leaves. | (Pandith et al., 2012, Sangnim et al., 2022, Li et al., 2024, Abdul Latif et al., 2024) |
| *Cichorium intybus* | *Cichorium intybus* L. [Asteraceae] | Accepted name | Asteraceae | [wfo-0000080671](https://www.worldfloraonline.org/taxon/wfo-0000080671) | Cichorii intybi radix (EMA/HMPC/121816/2010; root material only) | Leaf-derived callus extract used to biosynthesize silver nanoparticles (Ci-AgNPs); aqueous seed extract and seed-extract-mediated silver and gold nanoparticles; methanolic aerial-part, leaf, and root extracts; root fractions; and isolated β-sitosterol. | (Süntar et al., 2012, Gharari et al., 2022, Khalid et al., 2025) |
| *Croton lechleri* | *Croton lechleri* Müll.Arg. [Euphorbiaceae] | Accepted name | Euphorbiaceae | [wfo-0000931464](https://www.worldfloraonline.org/taxon/wfo-0000931464) | N/A | Trunk sap/latex (“dragon’s blood”) formulated as topical creams; crude resin/latex and isolated taspine hydrochloride. | (Vaisberg et al., 1989, Namjoyan et al., 2016, Rosa et al., 2026) |
| *Curcuma aromatica* | *Curcuma aromatica* Salisb. [Zingiberaceae] | Accepted name | Zingiberaceae | [wfo-0000365436](https://www.worldfloraonline.org/taxon/wfo-0000365436) | N/A | Rhizome-derived dialyzed crude aqueous enzyme fraction and purified CAP-II serine protease. | (Shivalingu et al., 2015, Shivalingu et al., 2016) |
| *Curcuma longa* | *Curcuma longa* L. [Zingiberaceae] | Accepted name | Zingiberaceae | [wfo-0000365771](https://www.worldfloraonline.org/taxon/wfo-0000365771) | *Curcumae longae* rhizoma EMA/HMPC/329755/2017 (Publication: 24/1/2019)  Turmeric (EMA/697301/20188) | Rhizome-derived dialyzed crude aqueous enzyme fraction. | (Shivalingu et al., 2015) |
| *Ficus drupacea* | *Ficus drupacea* Thunb. [Moraceae] | Accepted name | Moraceae | [wfo-0000688167](https://www.worldfloraonline.org/taxon/wfo-0000688167) | N/A | Purified drupin, a latex-derived cysteine protease. | (Manjuprasanna et al., 2021) |
| *Hamamelis virginiana* | *Hamamelis virginiana* L. [Hamamelidaceae] | Accepted name | Hamamelidaceae | [wfo-0001065240](https://www.worldfloraonline.org/taxon/wfo-0001065240) | Hamamelidis cortex (EMA/HMPC/114583/2008 Corr. 1) | Bark-derived proanthocyanidin and polysaccharide fractions. | (Deters et al., 2001, Wolff and Kieser, 2007) |
| *Inula viscosa* (L.) Aiton | *Dittrichia viscosa* (L.) Greuter [Asteraceae] | *Inula viscosa* is the name used in the original study; currently treated as a synonym of the accepted name | Asteraceae | [wfo-0000059214](https://list.worldfloraonline.org/wfo-0000059214) | N/A | Dried aerial parts combined 1:1 (w/w) with *Capsella bursa-pastoris* before ethanolic extraction. | (Öztürk et al., 2022) |
| *Jatropha curcas* | *Jatropha curcas* L. [Euphorbiaceae] | Accepted name | Euphorbiaceae | [wfo-0000219580](https://www.worldfloraonline.org/taxon/wfo-0000219580) | N/A | Latex solvent extract formulated as a topical spray; protease-enriched latex fraction. | (Urs et al., 2019, Tinpun et al., 2020) |
| *Myrtus communis* | *Myrtus communis* L. [Myrtaceae] | Accepted name | Myrtaceae | [wfo-0000248712](https://www.worldfloraonline.org/taxon/wfo-0000248712) | N/A. | Methanolic leaf-extract ointment; 6% aqueous berry-extract gel; aqueous leaf extract and topical gels. | (Ebrahimi et al., 2020, Khodaie et al., 2021, Jafari et al., 2024) |
| *Oxystelma esculentum* | *Oxystelma esculentum* (L.f.) Sm. [Apocynaceae] | Accepted name | Apocynaceae | [wfo-0001104832](https://www.worldfloraonline.org/taxon/wfo-0001104832) | N/A | Protein-rich, protease-enriched latex fraction. | (Urs et al., 2019) |
| *Panax notoginseng* | *Panax notoginseng* (Burkill) F.H.Chen [Araliaceae] | Accepted name | Araliaceae | [wfo-0000263666](https://www.worldfloraonline.org/taxon/wfo-0000263666) | Notoginseng Radix et Rhizoma (ChP 2020, Vol. I) | Root-derived isolated notoginsenoside Ft1; total P. notoginseng saponins (PNS); PNS-loaded gelatin/Bletilla striata gelatin formulation. | (Gao et al., 2014, Zhang et al., 2016, Men et al., 2020, Lei et al., 2022) |
| *Plantago major* | *Plantago major* L. [Plantaginaceae] | Accepted name | Plantaginaceae | [wfo-0000486544](https://www.worldfloraonline.org/taxon/wfo-0000486544) | N/A | Water- and ethanol-based leaf extracts; 10% hydroalcoholic leaf-extract topical gel. | (Zubair et al., 2016, Ghanadian et al., 2024) |
| *Potentilla erecta* | *Potentilla erecta* (L.) Raeusch. [Rosaceae] | Accepted name | Rosaceae | [wfo-0000984938](https://www.worldfloraonline.org/taxon/wfo-0000984938) | *Tormentillae* rhizoma (EMA/HMPC/5513/2010; rhizome material only) | Orally administered 80% methanolic rhizome extract; topically applied methanolic root extract. | (Kaltalioglu et al., 2020, Marcinczyk et al., 2021) |
| *Scoparia dulcis* | *Scoparia dulcis* L. [Plantaginaceae] | Accepted name | Plantaginaceae | [wfo-0000495138](https://www.worldfloraonline.org/taxon/wfo-0000495138) | N/A | Whole-plant aqueous decoction; aqueous leaf-extract ointment. | (Ediriweera et al., 2011, Thirumurthy et al., 2024) |
| *Symphytum officinale* | *Symphytum officinale* L. [Boraginaceae] | Accepted name | Boraginaceae | [wfo-0000432219](https://www.worldfloraonline.org/taxon/wfo-0000432219) | Symphyti radix (EMA/HMPC/572846/2009 Corr. 1; root material only) | 65% ethanolic root extract incorporated into 10% or 20% creams; prepared and commercial leaf extracts in gels, solutions, and emulsions. | (Araújo et al., 2012, Mârza et al., 2024) |
| *Tabernaemontana divaricata* | *Tabernaemontana divaricata* (L.) R.Br. ex Roem. & Schult. [Apocynaceae] | Accepted name | Apocynaceae | [wfo-0000319835](https://www.worldfloraonline.org/taxon/wfo-0000319835) | N/A | Purified latex cysteine protease; protease-enriched latex fraction; dialyzed crude latex enzyme extract. | (Singh et al., 2015, Urs et al., 2019, Singh et al., 2021) |
| *Tridax procumbens* | *Tridax procumbens* L. [Asteraceae] | Accepted name | Asteraceae | [wfo-0000086034](https://www.worldfloraonline.org/taxon/wfo-0000086034) | N/A | Ethanolic leaf extract; purified procumbenase obtained from an aqueous plant extract. | (Alshargabi et al., 2023, Gubbiveeranna et al., 2024) |
| *Urtica dioica* | *Urtica dioica* L. [Urticaceae] | Accepted name | Urticaceae | [wfo-0000416616](https://www.worldfloraonline.org/taxon/wfo-0000416616) | Urticae folium (EMA/HMPC/508015/2007); Urticae herba (EMA/HMPC/261302/2022) | Processed aqueous aerial-part extract and extract-containing ointments; hydroethanolic leaf extract. | (Zouari Bouassida et al., 2017, Kasouni et al., 2021) |
| *Vallaris solanacea* | *Vallaris solanacea* (Roth ex Roem. & Schult.) Kuntze [Apocynaceae] | Accepted name | Apocynaceae | [wfo-0000330951](https://www.worldfloraonline.org/taxon/wfo-0000330951) | N/A | Protease-enriched latex fraction and purified solanain. | (Somavarapu et al., 2018, Urs et al., 2019) |
| *Verbascum sinuatum* | *Verbascum sinuatum* L. [Scrophulariaceae] | Accepted name | Scrophulariaceae | [wfo-0000418899](https://www.worldfloraonline.org/taxon/wfo-0000418899) | N/A | Lyophilized aqueous leaf extract. | (Abdallah et al., 2022) |
| *Verbascum thapsus* | *Verbascum thapsus* L. [Scrophulariaceae] | Accepted name | Scrophulariaceae | [wfo-0000419130](https://www.worldfloraonline.org/taxon/wfo-0000419130) | N/A | Leaf-extract-loaded chitosan/gelatin cryogels; complete topical cream used in an episiotomy trial (plant part not specified). | (Taleb and Saeedi, 2021, Uzuner et al., 2025) |

Plant names in the first column reproduce the nomenclature used in the primary publications or in the retained review records; the second column gives the currently accepted name and complete authority. Accepted names, authorities, families, and WFO identifiers were verified against the World Flora Online Plant List, release 2026-06 (accessed July 25, 2026). When a primary publication used a synonym, the accepted name and a nomenclatural explanation are provided. Botanical/pharmacopoeial drug names were included only when a recognized designation could be linked to the species and plant part. EMA reference numbers correspond to the current final European Union herbal monographs adopted by the Committee on Herbal Medicinal Products (HMPC), rather than to the associated public summaries, assessment reports, opinions, addenda, or calls for scientific data. A pharmacopoeial designation identifies the recognized herbal substance and does not imply that every extract, fraction, essential oil, formulation, or study material in the same row complied with that monograph. Where a monograph is organ-specific, the designation applies only to the matching material (notably *Cichorii intybi* radix, *Tormentillae rhizoma*, and *Symphyti radix*). N/A indicates that no applicable recognized drug name was verified or that the tested material did not correspond to the recognized pharmacopoeial drug (EMA/HMPC). Each botanical component of a multicomponent preparation is listed separately; therefore, *Bletilla striata* and the species reported as *Inula viscosa* are included. For the *Inula viscosa–Capsella bursa-pastoris* mixture, the two dried aerial parts were combined 1:1 (w/w) before ethanolic extraction. Plant parts and botanical materials were transcribed from the primary studies and were not inferred when unreported. Scientific names are italicized; author citations, family names, and pharmacopoeial drug names are not italicized.

*Abbreviations:* BSGT, *Bletilla striata* gelatin; CAP-II, *Curcuma aromatica* protease II; ChP, Pharmacopoeia of the People’s Republic of China; EMA, European Medicines Agency; HMPC, Committee on Herbal Medicinal Products; NA, not applicable or no applicable recognized designation verified; PNS, *Panax notoginseng* saponins; POWO, Plants of the World Online ([**http://www.plantsoftheworldonline.org**](http://www.plantsoftheworldonline.org/)); PVA, poly(vinyl alcohol); WFO, World Flora Online; w/w, weight/weight.

**References**

Abdallah, L., Surakji, I., Qawasme, T., Ayyash, D., Shhadeh, R., Omar, G. and Barakat, A. (2022). In vitro activity of some medicinal plants on blood coagulation. *Turk J Pharm Sci,* 19**,** 330–335. <https://doi.org/10.4274/tjps.galenos.2021.14603>.

Abdul Latif, M., Mustafa, A., Keong, L. C. and Hamid, A. (2024). Chromolaena odorata layered-nitrile rubber polymer transdermal patch enhanced wound healing in vivo. *PLoS One,* 19**,** e0295381. <https://doi.org/10.1371/journal.pone.0295381>.

Al Dhamary, N., Al Adhal, A., Kadi, H. and Al Kamarany, M. A. (2024). Investigation effect of aloe vera in fresh gel form on rabbit’s model wound. *Journal of Complementary and Alternative Medical Research,* 25**,** 17–26. <https://doi.org/10.9734/jocamr/2024/v25i3522>.

Alshargabi, A., Mansour, W. and Al-Madhagi, W. M. (2023). Effect of tridax procumbens extracts on coagulation time according to gender and blood groups a and o: In vitro study. *Journal of Herbal Medicine,* 42**,** 100806. <https://doi.org/10.1016/j.hermed.2023.100806>.

Araújo, L. U., Reis, P. G., Barbosa, L. C., Saúde-Guimarães, D. A., Grabe-Guimarães, A., Mosqueira, V. C., Carneiro, C. M. and Silva-Barcellos, N. M. (2012). In vivo wound healing effects of symphytum officinale l. Leaves extract in different topical formulations. *Pharmazie,* 67**,** 355–60.

Asadi, S. Y., Parsaei, P., Karimi, M., Ezzati, S., Zamiri, A., Mohammadizadeh, F. and Rafieian-Kopaei, M. (2013). Effect of green tea (camellia sinensis) extract on healing process of surgical wounds in rat. *International Journal of Surgery,* 11**,** 332–337. <https://doi.org/10.1016/j.ijsu.2013.02.014>.

Ayyanahalli Matta, B. K., Kumar, S., Mehta, C. H., Nayak, U. Y. and Rodriguez, P. G. (2022). Comparative evaluation of the effectiveness of a combination of absorbable gelatin sponge and calendula officinalis with absorbable gelatin sponge used alone as a hemostatic agent-an in-vitro study. *Dent J (Basel),* 10**,** 76. <https://doi.org/10.3390/dj10050076>.

Bindhu, O. S. and Singh, M. K. (2014). Hemostatic, milk clotting and blood stain removal potential of cysteine proteases from calotropis gigantea (l.) r. Br. Latex. *Pharmacogn Mag,* 10**,** S350–6. <https://doi.org/10.4103/0973-1296.133294>.

Boudjelal, A., Smeriglio, A., Ginestra, G., Denaro, M. and Trombetta, D. (2020). Phytochemical profile, safety assessment and wound healing activity of artemisia absinthium l. *Plants (Basel),* 9**,** 1744. <https://doi.org/10.3390/plants9121744>.

Buzzi, M., De Freitas, F. and De Barros Winter, M. (2016). Therapeutic effectiveness of a calendula officinalis extract in venous leg ulcer healing. *J Wound Care,* 25**,** 732–739. <https://doi.org/10.12968/jowc.2016.25.12.732>.

Cao, P., Xie, P., Wang, X., Wang, J., Wei, J. and Kang, W. Y. (2017). Chemical constituents and coagulation activity of agastache rugosa. *BMC Complement Altern Med,* 17**,** 93. <https://doi.org/10.1186/s12906-017-1592-8>.

Deters, A., Dauer, A., Schnetz, E., Fartasch, M. and Hensel, A. (2001). High molecular compounds (polysaccharides and proanthocyanidins) from hamamelis virginiana bark: Influence on human skin keratinocyte proliferation and differentiation and influence on irritated skin. *Phytochemistry,* 58**,** 949–958. <https://doi.org/10.1016/S0031-9422(01)00361-2>.

Duran, V., Matic, M., Jovanovć, M., Mimica, N., Gajinov, Z., Poljacki, M. and Boza, P. (2005). Results of the clinical examination of an ointment with marigold (calendula officinalis) extract in the treatment of venous leg ulcers. *Int J Tissue React,* 27**,** 101–6.

Ebrahimi, F., Mahmoudi, J., Torbati, M., Karimi, P. and Valizadeh, H. (2020). Hemostatic activity of aqueous extract of myrtus communis l. Leaf in topical formulation: In vivo and in vitro evaluations. *J Ethnopharmacol,* 249**,** 112398. <https://doi.org/10.1016/j.jep.2019.112398>.

Ediriweera, E. R., Jayakody, J. R. and Ratnasooriya, W. D. (2011). Pro blood clotting activity of scoparia dulcis in rats. *Ayu,* 32**,** 271–4. <https://doi.org/10.4103/0974-8520.92547>.

Gao, B., Huang, L., Liu, H., Wu, H., Zhang, E., Yang, L., Wu, X. and Wang, Z. (2014). Platelet p2y₁₂ receptors are involved in the haemostatic effect of notoginsenoside ft1, a saponin isolated from panax notoginseng. *Br J Pharmacol,* 171**,** 214–23. <https://doi.org/10.1111/bph.12435>.

Ghalandari, S., Kariman, N., Sheikhan, Z., Mojab, F., Mirzaei, M. and Shahrahmani, H. (2017). Effect of hydroalcoholic extract of capsella bursa pastoris on early postpartum hemorrhage: A clinical trial study. *J Altern Complement Med,* 23**,** 794–799. <https://doi.org/10.1089/acm.2017.0095>.

Ghanadian, M., Soltani, R., Homayouni, A., Khorvash, F., Jouabadi, S. M. and Abdollahzadeh, M. (2024). The effect of plantago major hydroalcoholic extract on the healing of diabetic foot and pressure ulcers: A randomized open-label controlled clinical trial. *Int J Low Extrem Wounds,* 23**,** 475–481. <https://doi.org/10.1177/15347346211070723>.

Gharari, Z., Hanachi, P., Sadeghinia, H. and Walker, T. R. (2022). Cichorium intybus bio-callus synthesized silver nanoparticles: A promising antioxidant, antibacterial and anticancer compound. *International Journal of Pharmaceutics,* 625**,** 122062. <https://doi.org/10.1016/j.ijpharm.2022.122062>.

Ghasemi, M. R., Ranjbar, A., Tamri, P., Pourmoslemi, S., Nourian, A. and Dastan, D. (2023). In vitro antibacterial activity and wound healing effects of achillea millefolium essential oil in rat. *J Pharmacopuncture,* 26**,** 167–174. <https://doi.org/10.3831/kpi.2023.26.2.167>.

Gubbiveeranna, V., Megha, G. T., Kusuma, C. G., Ravikumar, H., Thoyajakshi, R. S., Vijayakumar, S., Mathad, S. N., Nagaraju, S., Wazzan, H., Khan, A., Alzahrani, K. A. and Malash, A. M. (2024). Effect of 'procumbenase' a serine protease from tridax procumbens aqueous extract on wound healing: A scar free healing of full thickness wounds. *Int J Biol Macromol,* 273**,** 133147. <https://doi.org/10.1016/j.ijbiomac.2024.133147>.

Hadavi-Siahboomi, M., Yegdaneh, A., Talebi, A. and Minaiyan, M. (2022). Ulcer-healing effect of hydroalcoholic extract and essential oil of achillea millefolium l. On murine model of colitis. *Int J Prev Med,* 13**,** 155. <https://doi.org/10.4103/ijpvm.ijpvm_50_22>.

Hashemipour, M. A., Lotfi, S., Torabi, M., Sharifi, F., Ansari, M., Ghassemi, A. and Sheikhshoaie, S. (2017). Evaluation of the effects of three plant species (myrtus communis l., camellia sinensis l., zataria multiflora boiss.) on the healing process of intraoral ulcers in rats. *J Dent (Shiraz),* 18**,** 127–135.

Jafari, Z., Bardania, H., Barmak, M. J., Eslami, S., Mahmoudi-Mourderaz, Y., Roustaei, N., Talebianpoor, M. S., Kokhdan, E. P. and Khoramrooz, S. S. (2024). Antimicrobial, anti-inflammatory, and wound healing properties of myrtus communis leaf methanolic extract ointment on burn wound infection induced by methicillin-resistant staphylococcus aureus in rats. *BioMed Research International,* 2024**,** 6758817. <https://doi.org/10.1155/2024/6758817>.

Jayasinghe, C. D., Ratnasooriya, W. D., Premakumara, S. and Udagama, P. V. (2022). Platelet augmentation activity of mature leaf juice of sri lankan wild type cultivar of carica papaya l: Insights into potential cellular mechanisms. *J Ethnopharmacol,* 296**,** 115511. <https://doi.org/10.1016/j.jep.2022.115511>.

Jettanacheawchankit, S., Sasithanasate, S., Sangvanich, P., Banlunara, W. and Thunyakitpisal, P. (2009). Acemannan stimulates gingival fibroblast proliferation; expressions of keratinocyte growth factor-1, vascular endothelial growth factor, and type i collagen; and wound healing. *Journal of pharmacological sciences,* 109**,** 525–531. <https://doi.org/10.1254/jphs.08204fp>.

Julien, G. K., Marcel, A. G., Antoinette, Y.-C. C. C., Hermann, A. M., Benoit, Y. D. C., Bernard, A. K., Karamoko, O., Mireille, D. and Joseph, D. A. (2023). An in-vitro study of the effect of hydroethanol extract of annona senegalensis leaves on bitis rhinoceros (viperidae) venom induced coagulopathy. *European Journal of Medicinal Plants,* 34**,** 33–41. <https://doi.org/10.9734/ejmp/2023/v34i11118>.

Kalalinia, F., Amiri, N., Mehrvarzian, N., Fazly Bazzaz, B. S., Iranshahi, M., Shahroodi, A., Arabzadeh, S., Abbaspour, M., Badiee Aaval, S. and Movaffagh, J. (2020). Topical green tea formulation with anti-hemorrhagic and antibacterial effects. *Iran J Basic Med Sci,* 23**,** 1085–1090. <https://doi.org/10.22038/ijbms.2020.41397.9782>.

Kaltalioglu, K., Balabanli, B. and Coskun-Cevher, S. (2020). Phenolic, antioxidant, antimicrobial, and in-vivo wound healing properties of potentilla erecta l. Root extract in diabetic rats. *Iran J Pharm Res,* 19**,** 264–274. <https://doi.org/10.22037/ijpr.2019.112411.13742>.

Kantesaria, R., Bagra, Y., Varma, P. and Panda, H. S. (2026). Plasticization of an ageratum conyzoides natural extract in crosslinked hydrogel: A promising material for combating wound infection. *Appl Biochem Biotechnol,* 198**,** 2750–2766. <https://doi.org/10.1007/s12010-026-05594-0>.

Kasouni, A. I., Chatzimitakos, T. G., Stalikas, C. D., Trangas, T., Papoudou-Bai, A. and Troganis, A. N. (2021). The unexplored wound healing activity of urtica dioica l. Extract: An in vitro and in vivo study. *Molecules,* 26**,** 6248. <https://doi.org/10.3390/molecules26206248>.

Khalid, A., Khurshid, S., Uroos, M., Khan, J., Sarwar, A., Ul Haq, E., Aziz, T., Alshehri, F., Al-Asmari, F., Baothman, B. K., Al-Joufi, F. A. and Alwethaynani, M. S. (2025). Green synthesis of silver and gold nanoparticles using seeds extract of cichorium intybus and their comparative analysis with commercially available ointment for wound healing activity. *J Mol Histol,* 56**,** 145. <https://doi.org/10.1007/s10735-025-10426-2>.

Khodaie, S. A., Emadi, F., Naseri, M., Kamalinejad, M., Riahi, S. M., Alijaniha, F. and Roghani, M. (2021). The effect of myrtus communis aqueous extract-containing gel on wound healing in streptozotocin-induced diabetic rats. *Curr Drug Discov Technol,* 18**,** 542–547. <https://doi.org/10.2174/1570163817666200712163956>.

Lei, T., Gao, Y., Duan, Y., Cui, C., Zhang, L. and Si, M. (2022). Panax notoginseng saponins improves healing of high glucose-induced wound through the gsk-3β/β-catenin pathway. *Environ Toxicol,* 37**,** 1867–1877. <https://doi.org/10.1002/tox.23533>.

Li, H.-F., Feng, H., Wang, Y., Pan, Z.-C., Yin, L., Qiu, H.-L., Qiao, H., Zhao, J.-Q., Xia, X.-Y., Hou, J.-C. and Wang, R.-X. (2024). Evaluation of hemostatic, anti-inflammatory, wound healing, skin irritation and allergy, and antimicrobial properties of active fraction from the ethanol extract of chromolaena odorata (l.) r.M. King & h. Rob. *Journal of Ethnopharmacology,* 331**,** 118330. <https://doi.org/10.1016/j.jep.2024.118330>.

Li, Y., Tan, Y., Zhao, H., Chen, S., Nilghaz, A., Cao, R. and Zhou, S. (2025). Green biosynthetic silver nanoparticles from ageratum conyzoides as multifunctional hemostatic agents: Combining hemostasis, antibacterial, and anti-inflammatory properties for effective wound healing. *Mater Today Bio,* 31**,** 101468. <https://doi.org/10.1016/j.mtbio.2025.101468>.

Manjuprasanna, V. N., Urs, A. P., Rudresha, G. V., Milan Gowda, M. D., Jayachandra, K., Hiremath, V., Rajaiah, R. and Vishwanath, B. S. (2021). Drupin, a thrombin-like protease prompts platelet activation and aggregation through protease-activated receptors. *J Cell Biochem,* 122**,** 870–881. <https://doi.org/10.1002/jcb.29917>.

Marcinczyk, N., Gołaszewska, A., Gromotowicz-Poplawska, A., Misztal, T., Strawa, J., Tomczyk, M., Kasacka, I. and Chabielska, E. (2021). Multidirectional effects of tormentil extract on hemostasis in experimental diabetes. *Front Pharmacol,* 12**,** 682987. <https://doi.org/10.3389/fphar.2021.682987>.

Mârza, S. M., Dăescu, A. M., Purdoiu, R. C., Dragomir, M., Tătaru, M., Melega, I., Nagy, A. L., Gal, A., Tăbăran, F., Bogdan, S., Moldovan, M., Pall, E., Munteanu, C., Magyari, K. and Papuc, I. (2024). Healing of skin wounds in rats using creams based on symphytum officinale extract. *Int J Mol Sci,* 25**,** 3099. <https://doi.org/10.3390/ijms25063099>.

Melenshia, D. S., Amirtham, S. M., Rebekah, G., Vinod, E. and Kachroo, U. (2024). Effect of reconstituted, lyophilized cold aqueous extract of aloe vera on human whole blood clotting time - a pilot study. *J Ayurveda Integr Med,* 15**,** 100887. <https://doi.org/10.1016/j.jaim.2024.100887>.

Men, S. Y., Huo, Q. L., Shi, L., Yan, Y., Yang, C. C., Yu, W. and Liu, B. Q. (2020). Panax notoginseng saponins promotes cutaneous wound healing and suppresses scar formation in mice. *J Cosmet Dermatol,* 19**,** 529–534. <https://doi.org/10.1111/jocd.13042>.

Naafe, M., Kariman, N., Keshavarz, Z., Khademi, N., Mojab, F. and Mohammadbeigi, A. (2018). Effect of hydroalcoholic extracts of capsella bursa-pastoris on heavy menstrual bleeding: A randomized clinical trial. *J Altern Complement Med,* 24**,** 694–700. <https://doi.org/10.1089/acm.2017.0267>.

Namjoyan, F., Kiashi, F., Moosavi, Z. B., Saffari, F. and Makhmalzadeh, B. S. (2016). Efficacy of dragon's blood cream on wound healing: A randomized, double-blind, placebo-controlled clinical trial. *J Tradit Complement Med,* 6**,** 37–40. <https://doi.org/10.1016/j.jtcme.2014.11.029>.

Öztürk, O. U., Ugur, M., Güzel, Y., Öztürk, M. A., Gürsoy, D., Doğan, S. and Temiz, M. (2022). Hemostatic effects of traditional inula viscosa and capsella bursa-pastoris plant mixture extract on rat liver parenchymal bleeding model. *Ulus Travma Acil Cerrahi Derg,* 28**,** 1059–1065. <https://doi.org/10.14744/tjtes.2022.90838>.

Pandith, H., Thongpraditchote, S., Wongkrajang, Y. and Gritsanapan, W. (2012). In vivo and in vitro hemostatic activity of chromolaena odorata leaf extract. *Pharm Biol,* 50**,** 1073–7. <https://doi.org/10.3109/13880209.2012.656849>.

Parente, L. M., Lino Júnior Rde, S., Tresvenzol, L. M., Vinaud, M. C., De Paula, J. R. and Paulo, N. M. (2012). Wound healing and anti-inflammatory effect in animal models of calendula officinalis l. Growing in brazil. *Evid Based Complement Alternat Med,* 2012**,** 375671. <https://doi.org/10.1155/2012/375671>.

Rajesh, R., Raghavendra Gowda, C. D., Nataraju, A., Dhananjaya, B. L., Kemparaju, K. and Vishwanath, B. S. (2005). Procoagulant activity of calotropis gigantea latex associated with fibrin(ogen)olytic activity. *Toxicon,* 46**,** 84–92. <https://doi.org/10.1016/j.toxicon.2005.03.012>.

Rosa, M. C. D. B., Campos, M. E. D. S. T., Albuquerque, A. S., Braz, M. S., Mapele, R. D. O., De Oliveira, M. C. G., Toma, H. S., Ferraz, P. F. P., Pereira, R. N. and Raymundo, D. L. (2026). Effect of croton lechleri sap-based topical treatment, with or without nitroglycerin, on healing of equine metatarsal skin wounds. *Veterinary and Animal Science,* 32**,** 100598. <https://doi.org/10.1016/j.vas.2026.100598>.

Sangnim, T., Meeboon, P., Phongsewalak, P., Prasongdee, P., Sriamornsak, P., Singh, I., Manmuan, S. and Huanbutta, K. (2022). Development and evaluation of liquid plaster loaded with chromolaena odorata leaf extract endowed with several beneficial properties to wound healing. *Gels (Basel, Switzerland)* 8**,** 72. <https://doi.org/10.3390/gels8020072>.

Sayahi, M., Jahangirimehr, A., Hatami Manesh, Z., Mojab, F. and Nikbina, M. (2024). The effect of camellia sinensis ointment on perineal pain and episiotomy wound healing in primiparous women: A triple-blind randomized clinical trial. *PLoS One,* 19**,** e0305048. <https://doi.org/10.1371/journal.pone.0305048>.

Shin, B., Hillyer, T. and Shin, W. S. (2024). Rational design and testing of antibacterial aloe vera hemostatic hydrogel. *Gels,* 10**,** 409. <https://doi.org/10.3390/gels10060409>.

Shivalingu, B. R., Vivek, H. K., Nafeesa, Z., Priya, B. S. and Swamy, S. N. (2015). Comparative analysis of procoagulant and fibrinogenolytic activity of crude protease fractions of turmeric species. *Journal of Ethnopharmacology,* 172**,** 261–264. <https://doi.org/10.1016/j.jep.2015.06.018>.

Shivalingu, B. R., Vivek, H. K., Priya, B. S., Soujanya, K. N. and Swamy, S. N. (2016). Purification and characterization of novel fibrin(ogen)olytic protease from curcuma aromatica salisb.: Role in hemostasis. *Phytomedicine,* 23**,** 1691–1698. <https://doi.org/10.1016/j.phymed.2016.09.007>.

Shrivastava, R., Cucuat, N. and John, G. W. (2007). Effects of alchemilla vulgaris and glycerine on epithelial and myofibroblast cell growth and cutaneous lesion healing in rats. *Phytother Res,* 21**,** 369–73. <https://doi.org/10.1002/ptr.2060>.

Singh, M. K., Rajagopalan, A., Tanimu, H. and Sukumaran, B. O. (2021). Purification, characterization and fibrino(geno)lytic activity of cysteine protease from tabernaemontana divaricata latex. *3 Biotech,* 11**,** 106. <https://doi.org/10.1007/s13205-021-02643-9>.

Singh, M. K., Usha, R., Hithayshree, K. R. and Bindhu, O. S. (2015). Hemostatic potential of latex proteases from tabernaemontana divaricata (l.) r. Br. Ex. Roem. And schult. And artocarpus altilis (parkinson ex. F.A. Zorn) forsberg. *J Thromb Thrombolysis,* 39**,** 43–9. <https://doi.org/10.1007/s11239-013-1012-y>.

Soib, H. H., Ismail, H. F., Husin, F., Abu Bakar, M. H., Yaakob, H. and Sarmidi, M. R. (2020). Bioassay-guided different extraction techniques of carica papaya (linn.) leaves on in vitro wound-healing activities. *Molecules (Basel, Switzerland),* 25**,** 517. <https://doi.org/10.3390/molecules25030517>.

Somavarapu, S., Vemula, S. and Reddy, I. B. (2018). Extraction, purification and characterization of a novel cysteine protease from the latex of plant vallaris solanacea. *Journal of Plant Biochemistry and Biotechnology,* 27**,** 186–198. <https://doi.org/10.1007/s13562-017-0429-3>.

Sultan, M. H., Zuwaiel, A. A., Moni, S. S., Alshahrani, S., Alqahtani, S. S., Madkhali, O. and Elmobark, M. E. (2020). Bioactive principles and potentiality of hot methanolic extract of the leaves from artemisia absinthium l "in vitro cytotoxicity against human mcf-7 breast cancer cells, antibacterial study and wound healing activity". *Curr Pharm Biotechnol,* 21**,** 1711–1721. <https://doi.org/10.2174/1389201021666200928150519>.

Süntar, I., Küpeli Akkol, E., Keles, H., Yesilada, E., Sarker, S. D. and Baykal, T. (2012). Comparative evaluation of traditional prescriptions from cichorium intybus l. For wound healing: Stepwise isolation of an active component by in vivo bioassay and its mode of activity. *J Ethnopharmacol,* 143**,** 299–309. <https://doi.org/10.1016/j.jep.2012.06.036>.

Taleb, S. and Saeedi, M. (2021). The effect of the verbascum thapsus on episiotomy wound healing in nulliparous women: A randomized controlled trial. *BMC Complement Med Ther,* 21**,** 166. <https://doi.org/10.1186/s12906-021-03339-6>.

Tasić-Kostov, M., Arsić, I., Pavlović, D., Stojanović, S., Najman, S., Naumović, S. and Tadić, V. (2019). Towards a modern approach to traditional use: In vitro and in vivo evaluation of alchemilla vulgaris l. Gel wound healing potential. *J Ethnopharmacol,* 238**,** 111789. <https://doi.org/10.1016/j.jep.2019.03.016>.

Teplicki, E., Ma, Q., Castillo, D. E., Zarei, M., Hustad, A. P., Chen, J. and Li, J. (2018). The effects of aloe vera on wound healing in cell proliferation, migration, and viability. *Wounds : a compendium of clinical research and practice,* 30**,** 263–268.

Thirumurthy, P., Natarajan, S., Tharumasivam, S. V., Dhailappan, A., Jayaraj, V. E. C., Doss, A. M. D. C. and Kumar, P. R. (2024). Phytochemical and biological evaluation of scoparia dulcis targeting wound healing efficacy. *African Journal of Biomedical Research,* 27**,** 184–192. <https://doi.org/10.53555/AJBR.v28i3S.7522>.

Tinpun, K., Nakpheng, T., Padmavathi, A. R. and Srichana, T. (2020). In vitro studies of jatropha curcas l. Latex spray formulation for wound healing applications. *Turk J Pharm Sci,* 17**,** 271–279. <https://doi.org/10.4274/tjps.galenos.2019.69875>.

Urs, A. P., Rudresha, G. V., Manjuprasanna, V. N., Suvilesh, K. N., Gowda, M. D. M., Yariswamy, M., Hiremath, V., Ramakrishnan, C., Savitha, M. N., Jayachandra, K., Sharanappa, P. and Vishwanath, B. S. (2019). Plant latex thrombin-like cysteine proteases alleviates bleeding by bypassing factor viii in murine model. *J Cell Biochem,* 120**,** 12843–12858. <https://doi.org/10.1002/jcb.28555>.

Uzuner, H., Yürük, A. and İșoğlu, İ. A. (2025). A potential hemostatic chitosan/gelatin cryogel impregnated with verbascum thapsus leaf extract for noncompressible hemorrhage management. *Biomedical Materials,* 20**,** 065026. <https://doi.org/10.1088/1748-605X/ae19b7>.

Vaisberg, A. J., Milla, M., Planas, M. C., Cordova, J. L., De Agusti, E. R., Ferreyra, R., Mustiga, M. C., Carlin, L. and Hammond, G. B. (1989). Taspine is the cicatrizant principle in sangre de grado extracted from croton lechleri. *Planta Med,* 55**,** 140–3. <https://doi.org/10.1055/s-2006-961907>.

Vijayakumar, G., Sundaram, G. A., Kumar, S. P., Lakshmanan, S., Krishnan, M. and Krishna, V. K. (2024). Comparison of efficacy of topical carica papaya leaf extract and hemocoagulase in postoperative wound healing after therapeutic orthodontic premolar extractions: A split mouth study. *Cureus,* 16**,** e61946. <https://doi.org/10.7759/cureus.61946>.

Wolff, H. H. and Kieser, M. (2007). Hamamelis in children with skin disorders and skin injuries: Results of an observational study. *European Journal of Pediatrics,* 166**,** 943–948. <https://doi.org/10.1007/s00431-006-0363-1>.

Zhang, E., Gao, B., Yang, L., Wu, X. and Wang, Z. (2016). Notoginsenoside ft1 promotes fibroblast proliferation via pi3k/akt/mtor signaling pathway and benefits wound healing in genetically diabetic mice. *J Pharmacol Exp Ther,* 356**,** 324–32. <https://doi.org/10.1124/jpet.115.229369>.

Zouari Bouassida, K., Bardaa, S., Khimiri, M., Rebaii, T., Tounsi, S., Jlaiel, L. and Trigui, M. (2017). Exploring the urtica dioica leaves hemostatic and wound-healing potential. *Biomed Res Int,* 2017**,** 1047523. <https://doi.org/10.1155/2017/1047523>.

Zubair, M., Nybom, H., Lindholm, C., Brandner, J. M. and Rumpunen, K. (2016). Promotion of wound healing by plantago major l. Leaf extracts – ex-vivo experiments confirm experiences from traditional medicine. *Natural Product Research,* 30**,** 622–624. <https://doi.org/10.1080/14786419.2015.1034714>.
