# Supplementary material for: Plant-derived modulators of the hemostasis-wound healing axis: mechanisms, evidence, and translational potential
Source: Front Pharmacol. 2026 Sep 29;17:1902225. doi: 10.3389/fphar.2026.1902225 (PMC13659225; doi:10.3389/fphar.2026.1902225)
Supplement: Supplementary file 4 [file DataSheet4.docx]

**Supplementary Table S4.** Official herbal drug designations, experimental botanical preparations, formulation status, and article-level traceability of the evaluated materials.

| **Accepted botanical name and authority (Validated via POWO)** | **Latin name** | **English common name** | **Reference Number (EMA/HMPC)** | **Experimental preparation or extract** | **Formulation status and composition** | **Voucher specimen and taxonomic authentication** | **Material source and batch/lot information** | **References** |
| --- | --- | --- | --- | --- | --- | --- | --- | --- |
| *Achillea millefolium* L. [Asteraceae] | Millefolii herba | Yarrow | EMA/HMPC/376416/2019 | Essential oil; 80% hydroalcoholic extract of aerial parts; hydrodistilled essential oil. | Formulated: 1%, 2%, and 3% (w/w) essential-oil ointments in Eucerin. Unformulated: hydroalcoholic extract and essential oil evaluated as study materials. | Authentication, voucher/repository: NR.  Authentication: Checked by Afasaneh Yegdaneh, pharmacognosist, Isfahan University of Medical Sciences; voucher/repository: Herbarium sample No. 1398, Isfahan School of Pharmacy | Source: commercial oil; botanical collection source, N/A; supplier/batch: Shafa Kurdistan Inc.; batch NR  Source: Tashal, Oshtorankooh, Lorestan Province, Iran; ~2400 m; supplier/batch: Batch N/A | (Ghasemi et al., 2023, Hadavi-Siahboomi et al., 2022) |
| *Agastache rugosa* (Fisch. & C.A.Mey.) Kuntze [Lamiaceae] | N/A | N/A | N/A | 70% ethanolic aerial-part extract; petroleum-ether, ethyl-acetate, and n-butanol fractions; isolated acacetin and tilianin. | Unformulated extract, solvent fractions, and isolated constituents; no finished dosage form reported. | Authentication: Identified by Prof. Changqin Li; voucher/repository: 201310231; Institute of Traditional Chinese Medicine, Henan University (Kaifeng, Henan, China). | Source: Suzhou region, Jiangsu Province, China; October 2013; supplier/batch: Batch N/A | (Cao et al., 2017) |
| *Ageratum conyzoides* L. [Asteraceae] | N/A | N/A | N/A | Aqueous extract of aerial flowers and leaves used for green synthesis of AgNPs. | Biogenic nanomaterial: aqueous plant extract reacted with AgNO3; isolated AC-AgNPs were washed and freeze-dried. No conventional excipient-based dosage form reported. | Authentication, voucher/repository: NR | Source: Along Meise River, Haikou City, China; summer 2022; supplier/batch: Batch N/A | (Li et al., 2025, Kantesaria et al., 2026) |
| *Alchemilla vulgaris* L. [Rosaceae] | N/A | N/A | N/A | Aerial-part aqueous, ethanolic, propylene-glycol, and standardized hydroglycerinated fluid extracts. | Unformulated extracts and extract-loaded hydrogels; GMP-manufactured hydroglycerinated fluid extract evaluated at 0.1–1% in vitro and as a 3% glycerine-based topical preparation. The standardized extract contained 2.67% tannins, including ellagic acid, quercetin (0.67%), and luteolin (0.0069%) by HPLC. | Authentication: Identified by Prof. Branislava Lakušić; voucher/repository: 02290117; Department of Botany, Faculty of Pharmacy, University of Belgrade.  Authentication, voucher/repository: NR | Source: Commercial sample; supplier/batch: Institute for Medicinal Plants Research “Dr Josif Pančić”; sample identifier 302041  Source: hydroglycerinated fluid extract of A. vulgaris was Manufactured by Laboratoires Biosphère-99, Les Martres-de-Veyre, France, under GMP requirements; batch no. B 3765 | (Tasić-Kostov et al., 2019, Shrivastava et al., 2007) |
| *Aloe vera* (L.) Burm.f. [Asphodelaceae] | N/A | N/A | N/A | Fresh inner-leaf gel; commercial liquid containing 90% inner-leaf gel; acemannan isolated from inner-leaf gel. | Fresh gel; commercial liquid gel preparation; 0.5–2% acemannan incorporated into Carbopol gels.  The optimized ratio of Aloe gel:Carbomer:lotus-root powder:cuttlefish-bone powder was 88:2:8:2. Selected antimicrobial variants contained amoxicillin or meropenem. | Authentication, voucher/repository: NR.  Authentication, voucher/repository: NR  Authentication: Identified by Assoc. Prof. Dr Chaiyo Chaichantipyuth, Department of Pharmacognosy, Faculty of Pharmaceutical Sciences, Chulalongkorn University.; voucher/repository: AC-05-2007; Oral Biology Research Center, Faculty of Dentistry, Chulalongkorn University, Bangkok, Thailand. | Source: Mountain area around Sana’a City, Yemen; collection date NR.; supplier/batch: Fresh Aloe vera leaves collected by the investigators; inner gel fillet used; batch/lot N/A.  Source: N/A-commercial preparation; supplier/batch: Manufacturer/batch NR  Source: Obtained from a local herbal supplier at Chatuchak (Jatujak) Market, Thailand; collection date N/A.; supplier/batch: Commercial botanical material from local supplier; batch/lot NR. | (Al Dhamary et al., 2024, Teplicki et al., 2018, Jettanacheawchankit et al., 2009, Melenshia et al., 2024, Shin et al., 2024) |
| *Annona senegalensis* Pers. [Annonaceae] | N/A | N/A | N/A | Hydroethanolic leaf extract. | Unformulated crude extract; no finished dosage form reported. | Authentication: Authenticated at Centre National de Floristique; voucher/repository: UCI001073; Centre National de Floristique, Université Félix Houphouët-Boigny | Source: Leaves harvested April 2019, morning; supplier/batch: Batch N/A | (Julien et al., 2023) |
| *Artemisia absinthium* L. [Asteraceae] | Absinthii herba | Wormwood | EMA/HMPC/751490/2016 Corr. 1 | Methanolic extract of flowering aerial parts; hot methanolic leaf-extract reaction mixture. | Formulated: extract-containing ointments. Unformulated: hot methanolic leaf-extract reaction mixture. | Authentication: Authenticated at University of M’sila; voucher/repository: AB-13; Department of Natural and Life Sciences, University of M’sila.  Authentication: Identified by a taxonomist at Jazan University; voucher/repository: JAZUH 1620; Herbarium, Jazan University | Source: M’sila Province, Algeria; May 2018; supplier/batch: Batch N/A.  Source: Locally harvested bundle purchased at a market in Jazan, Saudi Arabia; supplier/batch: Market lot NR | (Boudjelal et al., 2020, Sultan et al., 2020) |
| *Bletilla striata* (Thunb.) Rchb.f. [Orchidaceae] | N/A | N/A | N/A | Material reported only as Bletilla striata gelatin (BSGT). | Composite porous material containing gelatin, BSGT, and PNS. | Study-linked taxonomic authentication and voucher/repository for the material termed Bletilla striata gelatin: NR. | Source plant organ, botanical source, collection locality/date, BSGT supplier, and batch/lot NR. | (Lei et al., 2022) |
| *Calendula officinalis* L. [Asteraceae] | Calendulae flos | Calendula Flower | EMA/HMPC/437450/2017 | Standardized Calendula extract product; flower extract and solvent fractions; marigold extract. | Formulated: standardized topical product and extract ointment. Unformulated: flower extract and solvent fractions.  7% *Calendula officinalis* oil loaded into an absorbable gelatin sponge. Two loading procedures were described: direct homogeneous application of the oil to a 2 × 2 cm sponge followed by drying at 45 °C, and ethanol-assisted loading in which the oil was dispersed in 100 µL of ethanol, applied homogeneously to the pre-weighed sponge, and dried at room temperature (30–37 °C). Reported drug content: 56.28 ± 13.84%. | Authentication, voucher/repository: NR.  Authentication, voucher/repository: NR  Authentication: NR; voucher/repository: NR. | Source: N/A-commercial standardised extract; supplier/batch: Plenusdermax®/Phytoplenus, batch NR.  Source: Paraná State, Brazil, February 2006; supplier/batch: Empresa Clorofila, Goiânia, GO, batch NR.  Source: a Soxhlet-prepared marigold extract in a neutral ointment base was verified; botanical source, supplier, and batch/lot remain NR. | (Buzzi et al., 2016, Parente et al., 2012, Duran et al., 2005, Ayyanahalli Matta et al., 2022) |
| *Calotropis gigantea* (L.) W.T.Aiton [Apocynaceae] | N/A | N/A | N/A | Crude latex protein extract; partially purified and protease-enriched latex enzyme fractions. | Unformulated protein/enzyme preparations; no finished dosage form reported. | Authentication: Identified by P. Sharanappa.; voucher/repository: PS/214/2015; Herbarium, Department of Studies in Botany, Hemagangothri, University of Mysore, India.  Authentication: Study-linked botanical identification reported; voucher/repository: RRCBI/MCW/06; National Institute of Ayurveda and Dietetics, Jayanagar, Bangalore.  Authentication and voucher/repository: NR. | Source: Latex-yielding material collected in Hassan, Mandya, and Mysuru districts, Karnataka, India; collection date NR.; supplier/batch: Latex obtained from plant petioles/leaves/stems; batch/lot N/A.  Source: Tender plant parts collected in and around Bangalore, India; supplier/batch: Batch N/A.  Source: NR; supplier/batch: NR. | (Urs et al., 2019, Bindhu and Singh, 2014, Rajesh et al., 2005) |
| *Camellia sinensis* (L.) Kuntze [Theaceae] | *Camelliae sinensis* non fermentatum folium | Green tea | EMA/HMPC/283630/2012 | Green-tea extract; hydroalcoholic leaf extract. | Formulated: extract ointment, extract-loaded poly(vinyl alcohol) formulations, and a green-tea mucosal patch.  100 g of green-tea material were macerated in 1 L of 70% ethanol at room temperature for 48 h, filtered, concentrated by rotary evaporation, dried, and incorporated into pure Vaseline to obtain a 0.6% (w/w) ointment. | Authentication: Confirmed by herbarium, School of Pharmacy, Shahid Beheshti University of Medical Sciences; voucher/repository: Herbarium No. 8253  Authentication, voucher/repository: NR.  Authentication: the voucher samples were preserved for reference in the Herbarium of Pharmacognosy Department, School of Pharmacy, Kerman University of Medical Sciences, Kerman, Iran; voucher/repository: NR. | Source: Tehran pharmaceutical market; November 2019; supplier/batch: Market lot NR.  Source: Commercial dried leaves; supplier/batch: Golestan Co., Iran; batch NR.  Source: *Camellia sinensis* leaves collected during spring and summer in Baft, Kerman Province, Iran.; supplier/batch: Study-prepared plant extract/patch; batch/lot N/A. | (Sayahi et al., 2024, Kalalinia et al., 2020, Asadi et al., 2013, Hashemipour et al., 2017) |
| *Capsella bursa-pastoris* (L.) Medik. [Brassicaceae] | Bursae pastoris herba | Shepherds Purse | EMA/HMPC/262766/2010 | Hydroalcoholic extract obtained of powdered commercial herb in 96% ethanol; extract of dried aerial parts combined 1:1 (w/w) with dried aerial parts of *Inula viscosa (*Dittrichia viscosa*)* and extracted in absolute ethanol. | Combination extract: dried Capsella and Dittrichia viscosa aerial materials mixed 1:1 (w/w) before ethanolic extraction. Other formulations: oral capsules and hydroalcoholic extract in sublingual drops. | Authentication: Herbarium identification by study botanist Yelda Güzel; voucher/repository: Y. Güzel-3111; Herbarium, Department of Biology, HMKU.  Authentication: identity confirmed at the Laboratory of Botany, Faculty of Pharmacy, Shahid Beheshti University of Medical Sciences voucher/repository: NR.  Authentication: identity confirmed at the Botanical Laboratory, School of Pharmacy, Shahid Beheshti University of Medical Sciences; identifier and voucher specimen/repository NR. | Source: Hatay, Türkiye traditional-use material; supplier/batch: Batch N/A  Source: Purchased from an herbalist at Tabriz medicine market; batch/lot NR  Source: commercial plant material purchased from an herbalist at the Tabriz medicine market, Iran; collection locality/date and batch/lot NR. | (Öztürk et al., 2022, Naafe et al., 2018, Ghalandari et al., 2017) |
| *Carica papaya* L. [Caricaceae] | N/A | N/A | N/A | Commercial topical leaf-extract solution; mature leaf juice; methanolic leaf extracts; latex protease fraction; purified papain. | Formulated: commercial topical leaf-extract solution. Unformulated: leaf juice, methanolic extracts, protease-enriched latex fraction, and purified enzyme. | Authentication, voucher/repository: NA.  Authentication, voucher/repository: NR.  Authentication: Authenticated by Dr Shamsul Khamis; voucher/repository: SK 3143/17; Herbarium, Institute of Bioscience, Universiti Putra Malaysia.  Authentication: Identified by P. Sharanappa.; voucher/repository: PS/229/2015; Herbarium, Department of Studies in Botany, Hemagangothri, University of Mysore, India. | Source: Commercial product; supplier/batch: Caripill® 275 mg/5 mL; manufacturer/batch NR; Botroclot comparator.  Source: Papaya leaves representing locally available cultivars and agro-climatic regions in Sri Lanka; batch/lot N/A.  Source: Cultivar Eksotika, MARDI, Serdang, Selangor, Malaysia; supplier/batch: Batch N/A.  Source: Latex-yielding material collected in Hassan, Mandya, and Mysuru districts, Karnataka, India; batch/lot N/A. | (Vijayakumar et al., 2024, Jayasinghe et al., 2022, Soib et al., 2020, Urs et al., 2019) |
| *Chromolaena odorata* (L.) R.M.King & H.Rob. [Asteraceae] | N/A | N/A | N/A | Leaf extract; leaf-and-stem ethanol-eluted fractions; aqueous and ethanolic extracts of fresh or dried leaves. | Formulated: extract-loaded nitrile-rubber patch/liquid-plaster system. Unformulated: crude extracts and ethanol-eluted active fractions. | Authentication: Identified by Mohd Shahidan Mohamad Arshad, FRIM; voucher/repository: NR.  Authentication: Identified by Assoc. Prof. Jian-Ping Tian, School of Pharmacy, Hainan Medical University.; voucher/repository: FHMU7693; Herbarium of Hainan Medical University, Haikou, China.  Authentication: Identified by Dr Wandee Gritsanapan; voucher/repository: CO-003; Department of Pharmacognosy, Faculty of Pharmacy, Mahidol University. | Source: Fresh leaves; locality NR; supplier/batch: Patch supplied by Malaysian Rubber Board unit; batch NR.  Source: Fresh herb collected in October 2013 near Volcano Geological Park, Haikou, China.; supplier/batch: Leaves and stems extracted by the investigators; batch/lot N/A.  Source: Samut Sakhon Province, Thailand; December 2009; supplier/batch: Batch N/A. | (Abdul Latif et al., 2024, Li et al., 2024, Pandith et al., 2012, Sangnim et al., 2022) |
| *Cichorium intybus* L. [Asteraceae] | Cichorii intybi radix | Chicory root | EMA/HMPC/121816/2010 | Aqueous seed extract; seed-extract-mediated silver and gold nanoparticles; methanolic aerial-part, leaf, and root extracts; root fractions; isolated β-sitosterol. | Biogenic nanoparticle preparations and unformulated extracts/fractions/isolated constituent; no conventional finished dosage form was evaluated. | Authentication and voucher/repository: NR.  Authentication: Authenticated by Prof. Dr Hayri Duman; voucher/repository: GUE 2973; Herbarium, Faculty of Pharmacy, Gazi University. | Source: NR; supplier/batch: NR  Source: Borukkuyu Village, Altınekin, Konya, Türkiye; June 2009; supplier/batch: Batch N/A. | (Khalid et al., 2025, Süntar et al., 2012, Gharari et al., 2022) |
| *Croton lechleri* Müll.Arg. [Euphorbiaceae] | N/A | N/A | N/A | Trunk sap/latex (“dragon’s blood”); crude resin/latex; isolated taspine hydrochloride. | Formulated: topical creams containing sap/latex. Unformulated: crude latex/resin and isolated taspine hydrochloride. | Authentication, voucher/repository: NA.  Authentication. voucher/repository: NR.  Authentication, voucher/repository: NR. | Source: commercial Dragon’s Blood® *C. lechleri* oil; formulations compounded by Pharmácia de Manipulação e Homeopatia Melissa, Brazil, in nonionic cream; oil diluted 1:100 in sunflower oil; batch/lot NR.  Source: Commercial powder; supplier/batch: Maya Ethnobotanicals, Haarlem, the Netherlands; batch NR.  Source: Collected from *C. lechleri* trees in the Peruvian jungle; supplier/batch: Batch N/A. | (Rosa et al., 2026, Namjoyan et al., 2016, Vaisberg et al., 1989) |
| *Curcuma aromatica* Salisb. [Zingiberaceae] | N/A | N/A | N/A | Rhizome-derived dialyzed crude aqueous enzyme fraction; purified CAP-II serine protease. | Unformulated enzyme preparations; no finished dosage form reported. | Authentication: Authenticated by Dr D. Prasath; voucher/repository: 14SJCE051; Department of Biotechnology, Sri Jayachamarajendra College of Engineering.  Authentication: Plant material supplied by Dr D. Prasath, Senior Scientist, Department of Crop Improvement and Biotechnology, Indian Institute of Spices Research; voucher/repository: NR | Source: ICAR-Indian Institute of Spices Research Centre, Kozhikode, Kerala, India; supplier/batch: Batch NA  Source: Rhizomes supplied by the Indian Institute of Spices Research, Kozhikode, Kerala, India; collection date NR.; supplier/batch: Institutional plant supply; batch/lot NR. | (Shivalingu et al., 2016, Shivalingu et al., 2015) |
| *Curcuma longa* L. [Zingiberaceae] | Curcumae longae rhizoma | Turmeric | EMA/HMPC/329755/2017 (Publicación: 24/1/2019)  Turmeric (EMA/697301/20188) | Rhizome-derived dialyzed crude aqueous enzyme fraction. | Unformulated enzyme-rich extract; no finished dosage form reported. | Authentication: Plant material supplied by Dr D. Prasath, Senior Scientist, Department of Crop Improvement and Biotechnology, Indian Institute of Spices Research; voucher/repository: NR | Source: Rhizomes supplied by the Indian Institute of Spices Research, Kozhikode, Kerala, India; collection date NR.; supplier/batch: Institutional plant supply; batch/lot NR. | (Shivalingu et al., 2015) |
| *Dittrichia viscosa* (L.) Greuter [Asteraceae] | N/A | N/A | N/A | Dried aerial material extracted with ethanol after combination with *Capsella bursa-pastoris*. | Multicomponent preparation: dried aerial materials of *Dittrichia viscosa* (reported as *Inula viscosa*) and *Capsella bursa-pastoris* combined 1:1 (w/w) before ethanolic extraction. | Authentication: Herbarium identification by study botanist Yelda Güzel; voucher/repository: Y. Güzel-3110; Herbarium, Department of Biology, HMKU | Source: Hatay, Türkiye traditional-use material; exact collection details in full text; supplier/batch: Batch NA | (Öztürk et al., 2022) |
| *Ficus drupacea* Thunb. [Moraceae] | N/A | N/A | N/A | Fresh latex-derived plant latex protein-rich fraction (PL-PRF) and purified drupin. | Unformulated experimental protein preparations: crude PL-PRF used during latex screening and purified drupin used in the mechanistic assays. | Authentication: Plant material was identified and taxonomically authenticated by Dr. P. Sharanappa, Department of Studies in Botany, University of Mysore, Mysuru, India. voucher/repository: NR. | Source: Plant latex samples were collected from the surroundings of Mysuru and the Western Ghats region of Karnataka, India. Fresh latex was collected aseptically into sterile microcentrifuge tubes after excision of petioles, leaves, or stems, as described for the screened plant collection; supplier/batch: NR. | (Manjuprasanna et al., 2021) |
| *Hamamelis virginiana* L. [Hamamelidaceae] | Hamamelidis cortex | Hamamelis Bark | EMA/HMPC/114583/2008 Corr. 1 | Bark-derived proanthocyanidin and polysaccharide fractions. | Unformulated enriched fractions; no finished dosage form reported. | Authentication, voucher/repository: NR  Authentication, voucher/repository: NR. | Source: Commercial bark batch obtained in 1998; supplier/batch: Abtswinder Naturheilmittel, Abtswind, Germany.  Source: N/A - finished product; raw botanical collection source NR.; supplier/batch: Product manufacturer and batch/lot NR in the accessible article record. | (Wolff and Kieser, 2007, Deters et al., 2001) |
| *Jatropha curcas* L. [Euphorbiaceae] | N/A | N/A | N/A | Latex solvent extract; protease-enriched latex fraction. | Formulated: topical spray containing latex-derived extract. Unformulated: protease-enriched latex fraction. | Authentication: Study-linked voucher reported; voucher/repository: SKP 071100301.  Authentication: Identified by P. Sharanappa; voucher/repository: PS/241/2015; Herbarium, Department of Studies in Botany, Hemagangothri, University of Mysore, India. | Source: Botanical garden, Prince of Songkla University, Hat Yai, Thailand; latex from cut leaf stalks; supplier/batch: Stored at 4 °C; batch N/A  Source: Latex-yielding material collected in Hassan, Mandya, and Mysuru districts, Karnataka, India; collection date NR; supplier/batch: Latex obtained from plant petioles/leaves/stems; batch/lot N/A. | (Tinpun et al., 2020, Urs et al., 2019) |
| *Myrtus communis* L. [Myrtaceae] | N/A | N/A | N/A | Methanolic leaf extract; 6% aqueous berry extract; aqueous leaf extract. | Formulated: leaf-extract ointment, 6% berry-extract gel, and leaf-extract topical gels. | Authentication, voucher/repository: NR.  Authentication and voucher/repository: NR.  Authentication and voucher/repository: NR. | Source: Leaves collected during summer; precise locality and collection date NR.; supplier/batch: Study-prepared methanolic leaf extract/ointment; batch/lot N/A.  Source, supplier, and batch/lot NR.  Source: Leaves collected from shrubs in August 2017; precise location not accessible; supplier/batch: Study-prepared aqueous extract and gels; batch/lot N/A. | (Jafari et al., 2024, Khodaie et al., 2021, Ebrahimi et al., 2020) |
| *Oxystelma esculentum* (L.f.) Sm. [Apocynaceae] | N/A | N/A | N/A | Protein-rich, protease-enriched latex fraction. | Unformulated enriched latex fraction; no dosage form reported. | Authentication: Identified by P. Sharanappa.; voucher/repository: PS/223/2015; Herbarium, Department of Studies in Botany, Hemagangothri, University of Mysore, India. | Source: Latex-yielding material collected in Hassan, Mandya, and Mysuru districts, Karnataka, India; collection date NR.; supplier/batch: Latex obtained from plant petioles/leaves/stems; batch/lot N/A. | (Urs et al., 2019) |
| *Panax notoginseng* (Burkill) F.H.Chen [Araliaceae] | Notoginseng Radix et Rhizoma | Notoginseng root and rhizome | N/A | Root-derived isolated notoginsenoside Ft1; total PNS; PNS incorporated into a gelatin/*Bletilla striata* gelatin porous material. | Unformulated isolated compound and total-saponin preparation; formulated composite porous material containing gelatin, BSGT, and PNS. | Authentication/voucher for PNS and BSGT: NR; no Bletilla pharmacopoeial drug was inferred.  Authentication/voucher: N/A.  Authentication/voucher: N/A.  Authentication/voucher: N/A. | BSGT source plant organ, supplier, and batch/lot, and PNS supplier/batch: NR.  Source: PNS supplier, catalogue, and batch/lot NR.  Source: notoginsenoside Ft1 from Shanghai R&D Center for Standardization of Chinese Medicines; catalogue/batch NR. | (Lei et al., 2022, Men et al., 2020, Zhang et al., 2016, Gao et al., 2014) |
| *Plantago major* L. [Plantaginaceae] | N/A | N/A | N/A | Water- and ethanol-based leaf extracts; 10% hydroalcoholic leaf extract. | Formulated: 10% hydroalcoholic leaf-extract topical gel. Unformulated: aqueous and ethanolic leaf extracts. | Authentication, voucher/repository: NR  Authentication, voucher/repository: NR. | Source: Source of the Plantago major material used to prepare the clinical gel NR.; supplier/batch: Study-prepared 10% hydroalcoholic extract gel; raw-material supplier and batch/lot NR.  Source: Greenhouse-grown material, SLU Balsgård, Sweden; supplier/batch: Batch N/A. | (Ghanadian et al., 2024, Zubair et al., 2016) |
| *Potentilla erecta* (L.) Raeusch. [Rosaceae] | Tormentillae rhizoma | Tormentil | EMA/HMPC/5513/2010 | 80% methanolic rhizome extract; methanolic material reported as root extract. | Unformulated extracts administered orally or applied topically; no finished dosage form reported. | Authentication, voucher/repository: NR  Authentication: Identified by Dr Mustafa Karaköse; voucher/repository: ESPH 016; Herbarium, Vocational School of Espiye, Giresun University. | Source: Commercial powdered material; supplier/batch: Kawon, Gostyń, Poland; batch 268.2020420.2020  Source: Bulancak, Giresun Province, Türkiye; 40°42′18.5″ N, 38°03′15.2″ E; supplier/batch: Batch N/A. | (Marcinczyk et al., 2021, Kaltalioglu et al., 2020) |
| *Scoparia dulcis* L. [Plantaginaceae] | N/A | N/A | N/A | Whole-plant aqueous decoction; aqueous leaf extract. | Unformulated decoction; formulated leaf-extract ointment. | Authentication: Taxonomically identified by the Department of Botany, Bharathiar University, Tamil Nadu, India.; voucher/repository: NR  Authentication: Authenticated by the Department of Botany, University of Colombo, Sri Lanka.; voucher/repository: SN-32; museum of the Department of Zoology, University of Colombo, Sri Lanka. | Source: Leaves collected from local areas of Pollachi and Coimbatore, Tamil Nadu, India; collection date NR.; supplier/batch: Study-prepared aqueous leaf extract/ointment; batch/lot N/A.  Source: Fresh whole plants collected from paddy fields in Kalutara, Sri Lanka; collection date NR.; supplier/batch: Study-prepared aqueous decoction; batch/lot N/A. | (Thirumurthy et al., 2024, Ediriweera et al., 2011) |
| *Symphytum officinale* L. [Boraginaceae] | Symphyti radix | Comfrey Root | EMA/HMPC/572846/2009 Corr. 1 | 65% ethanolic root extract; prepared and commercial leaf extracts. | Root: 10% and 20% extract creams. Leaf: gels, solutions, and emulsions, including prepared and commercial products. | Authentication, voucher/repository: NR.  Authentication: Botanical identification reported; voucher/repository: OUPR 21806 | Source: Vâlcea area along the Olt River, Romania; February 2022; supplier/batch: Batch N/A.  Source: Material donated by Salão do Encontro, Betim, Minas Gerais, Brazil; supplier/batch: Batch NR | (Mârza et al., 2024, Araújo et al., 2012) |
| *Tabernaemontana divaricata* (L.) R.Br. ex Roem. & Schult. [Apocynaceae] | N/A | N/A | N/A | Purified latex cysteine protease; protease-enriched latex fraction; dialyzed crude latex enzyme extract. | Unformulated crude, enriched, and purified enzyme preparations; no dosage form reported. | Authentication: Study-linked voucher reported; voucher/repository: RRCBI-Mus/09; National Institute of Ayurveda and Dietetics, Jayanagar, Bangalore.  Authentication: Identified by P. Sharanappa.; voucher/repository: PS/205/2015; Herbarium, Department of Studies in Botany, Hemagangothri, University of Mysore, India.  Authentication: Voucher specimens were deposited for identification/authentication at the National Institute of Ayurveda and Dietetics, Jayanagar, Bangalore; named authenticator and exact code NR.; voucher/repository: Repository reported; exact voucher code NR. | Source: Twigs collected in and around Bangalore, India; supplier/batch: Batch N/A  Source: Latex-yielding material collected in Hassan, Mandya, and Mysuru districts, Karnataka, India; collection date NR.; supplier/batch: Latex obtained from plant petioles/leaves/stems; batch/lot N/A.  Source: Latex collected from plants in and around Bangalore, India; collection date NR.; supplier/batch: Study-prepared latex enzyme extract; batch/lot N/A. | (Singh et al., 2021, Urs et al., 2019, Singh et al., 2015) |
| *Tridax procumbens* L. [Asteraceae] | N/A | N/A | N/A | Ethanolic leaf extract; purified procumbenase obtained from an aqueous plant extract. | Unformulated crude extract and purified enzyme; no finished dosage form reported. | Authentication, voucher/repository: N/A.  Authentication: Identified by Hussin Ibrahim, Sana’a University; voucher/repository: Voucher 15; Department of Pharmacy, Al-Nasser University. | Source, supplier, batch/lot NR.  Collection locality/date not accessible snippet; supplier/batch: Batch N/A | (Gubbiveeranna et al., 2024, Alshargabi et al., 2023) |
| *Urtica dioica* L. [Urticaceae] | Urticae folium; Urticae herba | Nettle Leaf; Nettle Herb | EMA/HMPC/508015/2007; EMA/HMPC/261302/2022 | Processed aqueous aerial-part extract; hydroethanolic leaf extract. | Formulated: extract-containing ointments. Unformulated: processed aqueous aerial extract and hydroethanolic leaf extract. | Authentication: Plant sample identified; reference specimen retained at the University of Ioannina.; voucher/repository: UOI 120915; University of Ioannina, Greece.  Authentication: Identified by Prof. Mohamed Chaieb; voucher/repository: LBPes UD 02.15; Herbarium, Laboratory of Biopesticides, Centre of Biotechnology of Sfax. | Source: Aerial parts purchased from a local store in Ioannina, Epirus, Greece.; supplier/batch: Commercial local-store material; batch/lot NR.  Source: Sfax, Tunisia (35°14′58.36″ N, 11°7′17.75″ E); February 2015; supplier/batch: Batch N/A. | (Kasouni et al., 2021, Zouari Bouassida et al., 2017) |
| *Vallaris solanacea* (Roth ex Roem. & Schult.) Kuntze [Apocynaceae] | N/A | N/A | N/A | Protease-enriched latex fraction; purified solanain. | Unformulated enriched fraction and purified enzyme; no dosage form reported. | Authentication: Identified by P. Sharanappa.; voucher/repository: PS/208/2015; Herbarium, Department of Studies in Botany, Hemagangothri, University of Mysore, India.  Authentication, voucher/repository: NR. | Source: freshly collected V. solanacea latex; collection locality/date NR; study-prepared crude extract and purified solanain, batch N/A.  Source: Latex-yielding material collected in Hassan, Mandya, and Mysuru districts, Karnataka, India; collection date NR.; supplier/batch: Latex obtained from plant petioles/leaves/stems; batch/lot N/A. | (Urs et al., 2019, Somavarapu et al., 2018) |
| *Verbascum sinuatum* L. [Scrophulariaceae] | N/A | N/A | N/A | Aqueous leaf extract; lyophilized powder reconstituted in sterile distilled water at 50 mg/mL. | Unformulated aqueous leaf extract evaluated in vitro by PT and aPTT assays; no finished dosage form. | Authentication: Identified by Ghadeer Omar, An-Najah National University, Palestine; deposited at the An-Najah National University herbarium; voucher/repository: 1970 | Source: Nablus, West Bank, Palestine; collection date NR; supplier/batch: Batch/lot NR | (Abdallah et al., 2022) |
| *Verbascum thapsus* L. [Scrophulariaceae] | N/A | N/A | N/A | Leaf extract; complete topical cream in which the plant part was not specified. | Formulated: leaf-extract-loaded chitosan/gelatin cryogels and a multicomponent topical cream used in an episiotomy trial. | Authentication, voucher/repository: NR.  Authentication, voucher/repository: NR | Source: Leaves collected in Kayseri, Türkiye; collection date NR.; supplier/batch: Study-prepared methanolic leaf extract incorporated into cryogels; plant-material batch/lot NR.  Source, supplier/batch: NR | (Uzuner et al., 2025, Taleb and Saeedi, 2021) |

Botanical names and author citations follow the accepted taxonomic names verified in Supplementary Table S3. The columns “Latin name,” “English common name,” and “Reference number” and “Monograph/reference identifier” were completed only when both the experimental species and the evaluated plant organ corresponded to the herbal substance covered by the cited pharmacopoeial or EMA/HMPC monograph. The reference column reports the applicable EMA/HMPC document number or pharmacopoeial edition. Information on voucher specimens, taxonomic authentication, material source, supplier, and batch or lot number was extracted exclusively from the Methods section or an equivalent article section and was recorded only when explicitly linked to the experimental material or to the same botanical collection. Voucher specimens reported in unrelated studies were not substituted. NR indicates that the information was not reported in the reviewed full-text methodological record; and N/A indicates that the field was not applicable. For commercially obtained isolated compounds or finished products, a botanical voucher may be N/A; however, supplier or manufacturer details and catalogue, batch, or lot numbers were recorded when available. For the material reported as *Bletilla striata* gelatin, the source plant organ and botanical traceability were not reported

*Abbreviations:* AgNPs, silver nanoparticles; AC-AgNPs, *Ageratum conyzoides*-mediated silver nanoparticles; AgNO₃, silver nitrate; aPTT, activated partial thromboplastin time; BSGT, *Bletilla striata* gelatin; CAP-II, *Curcuma aromatica* protease II; EMA, European Medicines Agency; FRIM, Forest Research Institute Malaysia; GMP, Good Manufacturing Practice; HMKU, Hatay Mustafa Kemal University; HMPC, Committee on Herbal Medicinal Products; HPLC, high-performance liquid chromatography; ICAR, Indian Council of Agricultural Research; MARDI, Malaysian Agricultural Research and Development Institute; N/A, not applicable; NR, not reported in the reviewed full-text methodological record; PL-PRF, plant latex protein-rich fraction; PNS, *Panax notoginseng* saponins; POWO, Plants of the World Online ([**http://www.plantsoftheworldonline.org**](http://www.plantsoftheworldonline.org/)); PT, prothrombin time; R&D, research and development; SLU, Swedish University of Agricultural Sciences; w/w, weight by weight.

**References**

Abdallah, L., Surakji, I., Qawasme, T., Ayyash, D., Shhadeh, R., Omar, G. and Barakat, A. (2022). In vitro activity of some medicinal plants on blood coagulation. *Turk J Pharm Sci,* 19**,** 330–335. <https://doi.org/10.4274/tjps.galenos.2021.14603>.

Abdul Latif, M., Mustafa, A., Keong, L. C. and Hamid, A. (2024). Chromolaena odorata layered-nitrile rubber polymer transdermal patch enhanced wound healing in vivo. *PLoS One,* 19**,** e0295381. <https://doi.org/10.1371/journal.pone.0295381>.

Al Dhamary, N., Al Adhal, A., Kadi, H. and Al Kamarany, M. A. (2024). Investigation effect of aloe vera in fresh gel form on rabbit’s model wound. *Journal of Complementary and Alternative Medical Research,* 25**,** 17–26. <https://doi.org/10.9734/jocamr/2024/v25i3522>.

Alshargabi, A., Mansour, W. and Al-Madhagi, W. M. (2023). Effect of tridax procumbens extracts on coagulation time according to gender and blood groups a and o: In vitro study. *Journal of Herbal Medicine,* 42**,** 100806. <https://doi.org/10.1016/j.hermed.2023.100806>.

Araújo, L. U., Reis, P. G., Barbosa, L. C., Saúde-Guimarães, D. A., Grabe-Guimarães, A., Mosqueira, V. C., Carneiro, C. M. and Silva-Barcellos, N. M. (2012). In vivo wound healing effects of symphytum officinale l. Leaves extract in different topical formulations. *Pharmazie,* 67**,** 355–60.

Asadi, S. Y., Parsaei, P., Karimi, M., Ezzati, S., Zamiri, A., Mohammadizadeh, F. and Rafieian-Kopaei, M. (2013). Effect of green tea (camellia sinensis) extract on healing process of surgical wounds in rat. *International Journal of Surgery,* 11**,** 332–337. <https://doi.org/10.1016/j.ijsu.2013.02.014>.

Ayyanahalli Matta, B. K., Kumar, S., Mehta, C. H., Nayak, U. Y. and Rodriguez, P. G. (2022). Comparative evaluation of the effectiveness of a combination of absorbable gelatin sponge and calendula officinalis with absorbable gelatin sponge used alone as a hemostatic agent-an in-vitro study. *Dent J (Basel),* 10**,** 76. <https://doi.org/10.3390/dj10050076>.

Bindhu, O. S. and Singh, M. K. (2014). Hemostatic, milk clotting and blood stain removal potential of cysteine proteases from calotropis gigantea (l.) r. Br. Latex. *Pharmacogn Mag,* 10**,** S350–6. <https://doi.org/10.4103/0973-1296.133294>.

Boudjelal, A., Smeriglio, A., Ginestra, G., Denaro, M. and Trombetta, D. (2020). Phytochemical profile, safety assessment and wound healing activity of artemisia absinthium l. *Plants (Basel),* 9**,** 1744. <https://doi.org/10.3390/plants9121744>.

Buzzi, M., De Freitas, F. and De Barros Winter, M. (2016). Therapeutic effectiveness of a calendula officinalis extract in venous leg ulcer healing. *J Wound Care,* 25**,** 732–739. <https://doi.org/10.12968/jowc.2016.25.12.732>.

Cao, P., Xie, P., Wang, X., Wang, J., Wei, J. and Kang, W. Y. (2017). Chemical constituents and coagulation activity of agastache rugosa. *BMC Complement Altern Med,* 17**,** 93. <https://doi.org/10.1186/s12906-017-1592-8>.

Deters, A., Dauer, A., Schnetz, E., Fartasch, M. and Hensel, A. (2001). High molecular compounds (polysaccharides and proanthocyanidins) from hamamelis virginiana bark: Influence on human skin keratinocyte proliferation and differentiation and influence on irritated skin. *Phytochemistry,* 58**,** 949–958. <https://doi.org/10.1016/S0031-9422(01)00361-2>.

Duran, V., Matic, M., Jovanovć, M., Mimica, N., Gajinov, Z., Poljacki, M. and Boza, P. (2005). Results of the clinical examination of an ointment with marigold (calendula officinalis) extract in the treatment of venous leg ulcers. *Int J Tissue React,* 27**,** 101–6.

Ebrahimi, F., Mahmoudi, J., Torbati, M., Karimi, P. and Valizadeh, H. (2020). Hemostatic activity of aqueous extract of myrtus communis l. Leaf in topical formulation: In vivo and in vitro evaluations. *J Ethnopharmacol,* 249**,** 112398. <https://doi.org/10.1016/j.jep.2019.112398>.

Ediriweera, E. R., Jayakody, J. R. and Ratnasooriya, W. D. (2011). Pro blood clotting activity of scoparia dulcis in rats. *Ayu,* 32**,** 271–4. <https://doi.org/10.4103/0974-8520.92547>.

Gao, B., Huang, L., Liu, H., Wu, H., Zhang, E., Yang, L., Wu, X. and Wang, Z. (2014). Platelet p2y₁₂ receptors are involved in the haemostatic effect of notoginsenoside ft1, a saponin isolated from panax notoginseng. *Br J Pharmacol,* 171**,** 214–23. <https://doi.org/10.1111/bph.12435>.

Ghalandari, S., Kariman, N., Sheikhan, Z., Mojab, F., Mirzaei, M. and Shahrahmani, H. (2017). Effect of hydroalcoholic extract of capsella bursa pastoris on early postpartum hemorrhage: A clinical trial study. *J Altern Complement Med,* 23**,** 794–799. <https://doi.org/10.1089/acm.2017.0095>.

Ghanadian, M., Soltani, R., Homayouni, A., Khorvash, F., Jouabadi, S. M. and Abdollahzadeh, M. (2024). The effect of plantago major hydroalcoholic extract on the healing of diabetic foot and pressure ulcers: A randomized open-label controlled clinical trial. *Int J Low Extrem Wounds,* 23**,** 475–481. <https://doi.org/10.1177/15347346211070723>.

Gharari, Z., Hanachi, P., Sadeghinia, H. and Walker, T. R. (2022). Cichorium intybus bio-callus synthesized silver nanoparticles: A promising antioxidant, antibacterial and anticancer compound. *International Journal of Pharmaceutics,* 625**,** 122062. <https://doi.org/10.1016/j.ijpharm.2022.122062>.

Ghasemi, M. R., Ranjbar, A., Tamri, P., Pourmoslemi, S., Nourian, A. and Dastan, D. (2023). In vitro antibacterial activity and wound healing effects of achillea millefolium essential oil in rat. *J Pharmacopuncture,* 26**,** 167–174. <https://doi.org/10.3831/kpi.2023.26.2.167>.

Gubbiveeranna, V., Megha, G. T., Kusuma, C. G., Ravikumar, H., Thoyajakshi, R. S., Vijayakumar, S., Mathad, S. N., Nagaraju, S., Wazzan, H., Khan, A., Alzahrani, K. A. and Malash, A. M. (2024). Effect of 'procumbenase' a serine protease from tridax procumbens aqueous extract on wound healing: A scar free healing of full thickness wounds. *Int J Biol Macromol,* 273**,** 133147. <https://doi.org/10.1016/j.ijbiomac.2024.133147>.

Hadavi-Siahboomi, M., Yegdaneh, A., Talebi, A. and Minaiyan, M. (2022). Ulcer-healing effect of hydroalcoholic extract and essential oil of achillea millefolium l. On murine model of colitis. *Int J Prev Med,* 13**,** 155. <https://doi.org/10.4103/ijpvm.ijpvm_50_22>.

Hashemipour, M. A., Lotfi, S., Torabi, M., Sharifi, F., Ansari, M., Ghassemi, A. and Sheikhshoaie, S. (2017). Evaluation of the effects of three plant species (myrtus communis l., camellia sinensis l., zataria multiflora boiss.) on the healing process of intraoral ulcers in rats. *J Dent (Shiraz),* 18**,** 127–135.

Jafari, Z., Bardania, H., Barmak, M. J., Eslami, S., Mahmoudi-Mourderaz, Y., Roustaei, N., Talebianpoor, M. S., Kokhdan, E. P. and Khoramrooz, S. S. (2024). Antimicrobial, anti-inflammatory, and wound healing properties of myrtus communis leaf methanolic extract ointment on burn wound infection induced by methicillin-resistant staphylococcus aureus in rats. *BioMed Research International,* 2024**,** 6758817. <https://doi.org/10.1155/2024/6758817>.

Jayasinghe, C. D., Ratnasooriya, W. D., Premakumara, S. and Udagama, P. V. (2022). Platelet augmentation activity of mature leaf juice of sri lankan wild type cultivar of carica papaya l: Insights into potential cellular mechanisms. *J Ethnopharmacol,* 296**,** 115511. <https://doi.org/10.1016/j.jep.2022.115511>.

Jettanacheawchankit, S., Sasithanasate, S., Sangvanich, P., Banlunara, W. and Thunyakitpisal, P. (2009). Acemannan stimulates gingival fibroblast proliferation; expressions of keratinocyte growth factor-1, vascular endothelial growth factor, and type i collagen; and wound healing. *Journal of pharmacological sciences,* 109**,** 525–531. <https://doi.org/10.1254/jphs.08204fp>.

Julien, G. K., Marcel, A. G., Antoinette, Y.-C. C. C., Hermann, A. M., Benoit, Y. D. C., Bernard, A. K., Karamoko, O., Mireille, D. and Joseph, D. A. (2023). An in-vitro study of the effect of hydroethanol extract of annona senegalensis leaves on bitis rhinoceros (viperidae) venom induced coagulopathy. *European Journal of Medicinal Plants,* 34**,** 33–41. <https://doi.org/10.9734/ejmp/2023/v34i11118>.

Kalalinia, F., Amiri, N., Mehrvarzian, N., Fazly Bazzaz, B. S., Iranshahi, M., Shahroodi, A., Arabzadeh, S., Abbaspour, M., Badiee Aaval, S. and Movaffagh, J. (2020). Topical green tea formulation with anti-hemorrhagic and antibacterial effects. *Iran J Basic Med Sci,* 23**,** 1085–1090. <https://doi.org/10.22038/ijbms.2020.41397.9782>.

Kaltalioglu, K., Balabanli, B. and Coskun-Cevher, S. (2020). Phenolic, antioxidant, antimicrobial, and in-vivo wound healing properties of potentilla erecta l. Root extract in diabetic rats. *Iran J Pharm Res,* 19**,** 264–274. <https://doi.org/10.22037/ijpr.2019.112411.13742>.

Kantesaria, R., Bagra, Y., Varma, P. and Panda, H. S. (2026). Plasticization of an ageratum conyzoides natural extract in crosslinked hydrogel: A promising material for combating wound infection. *Appl Biochem Biotechnol,* 198**,** 2750–2766. <https://doi.org/10.1007/s12010-026-05594-0>.

Kasouni, A. I., Chatzimitakos, T. G., Stalikas, C. D., Trangas, T., Papoudou-Bai, A. and Troganis, A. N. (2021). The unexplored wound healing activity of urtica dioica l. Extract: An in vitro and in vivo study. *Molecules,* 26**,** 6248. <https://doi.org/10.3390/molecules26206248>.

Khalid, A., Khurshid, S., Uroos, M., Khan, J., Sarwar, A., Ul Haq, E., Aziz, T., Alshehri, F., Al-Asmari, F., Baothman, B. K., Al-Joufi, F. A. and Alwethaynani, M. S. (2025). Green synthesis of silver and gold nanoparticles using seeds extract of cichorium intybus and their comparative analysis with commercially available ointment for wound healing activity. *J Mol Histol,* 56**,** 145. <https://doi.org/10.1007/s10735-025-10426-2>.

Khodaie, S. A., Emadi, F., Naseri, M., Kamalinejad, M., Riahi, S. M., Alijaniha, F. and Roghani, M. (2021). The effect of myrtus communis aqueous extract-containing gel on wound healing in streptozotocin-induced diabetic rats. *Curr Drug Discov Technol,* 18**,** 542–547. <https://doi.org/10.2174/1570163817666200712163956>.

Lei, T., Gao, Y., Duan, Y., Cui, C., Zhang, L. and Si, M. (2022). Panax notoginseng saponins improves healing of high glucose-induced wound through the gsk-3β/β-catenin pathway. *Environ Toxicol,* 37**,** 1867–1877. <https://doi.org/10.1002/tox.23533>.

Li, H.-F., Feng, H., Wang, Y., Pan, Z.-C., Yin, L., Qiu, H.-L., Qiao, H., Zhao, J.-Q., Xia, X.-Y., Hou, J.-C. and Wang, R.-X. (2024). Evaluation of hemostatic, anti-inflammatory, wound healing, skin irritation and allergy, and antimicrobial properties of active fraction from the ethanol extract of chromolaena odorata (l.) r.M. King & h. Rob. *Journal of Ethnopharmacology,* 331**,** 118330. <https://doi.org/10.1016/j.jep.2024.118330>.

Li, Y., Tan, Y., Zhao, H., Chen, S., Nilghaz, A., Cao, R. and Zhou, S. (2025). Green biosynthetic silver nanoparticles from ageratum conyzoides as multifunctional hemostatic agents: Combining hemostasis, antibacterial, and anti-inflammatory properties for effective wound healing. *Mater Today Bio,* 31**,** 101468. <https://doi.org/10.1016/j.mtbio.2025.101468>.

Manjuprasanna, V. N., Urs, A. P., Rudresha, G. V., Milan Gowda, M. D., Jayachandra, K., Hiremath, V., Rajaiah, R. and Vishwanath, B. S. (2021). Drupin, a thrombin-like protease prompts platelet activation and aggregation through protease-activated receptors. *J Cell Biochem,* 122**,** 870–881. <https://doi.org/10.1002/jcb.29917>.

Marcinczyk, N., Gołaszewska, A., Gromotowicz-Poplawska, A., Misztal, T., Strawa, J., Tomczyk, M., Kasacka, I. and Chabielska, E. (2021). Multidirectional effects of tormentil extract on hemostasis in experimental diabetes. *Front Pharmacol,* 12**,** 682987. <https://doi.org/10.3389/fphar.2021.682987>.

Mârza, S. M., Dăescu, A. M., Purdoiu, R. C., Dragomir, M., Tătaru, M., Melega, I., Nagy, A. L., Gal, A., Tăbăran, F., Bogdan, S., Moldovan, M., Pall, E., Munteanu, C., Magyari, K. and Papuc, I. (2024). Healing of skin wounds in rats using creams based on symphytum officinale extract. *Int J Mol Sci,* 25**,** 3099. <https://doi.org/10.3390/ijms25063099>.

Melenshia, D. S., Amirtham, S. M., Rebekah, G., Vinod, E. and Kachroo, U. (2024). Effect of reconstituted, lyophilized cold aqueous extract of aloe vera on human whole blood clotting time - a pilot study. *J Ayurveda Integr Med,* 15**,** 100887. <https://doi.org/10.1016/j.jaim.2024.100887>.

Men, S. Y., Huo, Q. L., Shi, L., Yan, Y., Yang, C. C., Yu, W. and Liu, B. Q. (2020). Panax notoginseng saponins promotes cutaneous wound healing and suppresses scar formation in mice. *J Cosmet Dermatol,* 19**,** 529–534. <https://doi.org/10.1111/jocd.13042>.

Naafe, M., Kariman, N., Keshavarz, Z., Khademi, N., Mojab, F. and Mohammadbeigi, A. (2018). Effect of hydroalcoholic extracts of capsella bursa-pastoris on heavy menstrual bleeding: A randomized clinical trial. *J Altern Complement Med,* 24**,** 694–700. <https://doi.org/10.1089/acm.2017.0267>.

Namjoyan, F., Kiashi, F., Moosavi, Z. B., Saffari, F. and Makhmalzadeh, B. S. (2016). Efficacy of dragon's blood cream on wound healing: A randomized, double-blind, placebo-controlled clinical trial. *J Tradit Complement Med,* 6**,** 37–40. <https://doi.org/10.1016/j.jtcme.2014.11.029>.

Öztürk, O. U., Ugur, M., Güzel, Y., Öztürk, M. A., Gürsoy, D., Doğan, S. and Temiz, M. (2022). Hemostatic effects of traditional inula viscosa and capsella bursa-pastoris plant mixture extract on rat liver parenchymal bleeding model. *Ulus Travma Acil Cerrahi Derg,* 28**,** 1059–1065. <https://doi.org/10.14744/tjtes.2022.90838>.

Pandith, H., Thongpraditchote, S., Wongkrajang, Y. and Gritsanapan, W. (2012). In vivo and in vitro hemostatic activity of chromolaena odorata leaf extract. *Pharm Biol,* 50**,** 1073–7. <https://doi.org/10.3109/13880209.2012.656849>.

Parente, L. M., Lino Júnior Rde, S., Tresvenzol, L. M., Vinaud, M. C., De Paula, J. R. and Paulo, N. M. (2012). Wound healing and anti-inflammatory effect in animal models of calendula officinalis l. Growing in brazil. *Evid Based Complement Alternat Med,* 2012**,** 375671. <https://doi.org/10.1155/2012/375671>.

Rajesh, R., Raghavendra Gowda, C. D., Nataraju, A., Dhananjaya, B. L., Kemparaju, K. and Vishwanath, B. S. (2005). Procoagulant activity of calotropis gigantea latex associated with fibrin(ogen)olytic activity. *Toxicon,* 46**,** 84–92. <https://doi.org/10.1016/j.toxicon.2005.03.012>.

Rosa, M. C. D. B., Campos, M. E. D. S. T., Albuquerque, A. S., Braz, M. S., Mapele, R. D. O., De Oliveira, M. C. G., Toma, H. S., Ferraz, P. F. P., Pereira, R. N. and Raymundo, D. L. (2026). Effect of croton lechleri sap-based topical treatment, with or without nitroglycerin, on healing of equine metatarsal skin wounds. *Veterinary and Animal Science,* 32**,** 100598. <https://doi.org/10.1016/j.vas.2026.100598>.

Sangnim, T., Meeboon, P., Phongsewalak, P., Prasongdee, P., Sriamornsak, P., Singh, I., Manmuan, S. and Huanbutta, K. (2022). Development and evaluation of liquid plaster loaded with chromolaena odorata leaf extract endowed with several beneficial properties to wound healing. *Gels (Basel, Switzerland)* 8**,** 72. <https://doi.org/10.3390/gels8020072>.

Sayahi, M., Jahangirimehr, A., Hatami Manesh, Z., Mojab, F. and Nikbina, M. (2024). The effect of camellia sinensis ointment on perineal pain and episiotomy wound healing in primiparous women: A triple-blind randomized clinical trial. *PLoS One,* 19**,** e0305048. <https://doi.org/10.1371/journal.pone.0305048>.

Shin, B., Hillyer, T. and Shin, W. S. (2024). Rational design and testing of antibacterial aloe vera hemostatic hydrogel. *Gels,* 10**,** 409. <https://doi.org/10.3390/gels10060409>.

Shivalingu, B. R., Vivek, H. K., Nafeesa, Z., Priya, B. S. and Swamy, S. N. (2015). Comparative analysis of procoagulant and fibrinogenolytic activity of crude protease fractions of turmeric species. *Journal of Ethnopharmacology,* 172**,** 261–264. <https://doi.org/10.1016/j.jep.2015.06.018>.

Shivalingu, B. R., Vivek, H. K., Priya, B. S., Soujanya, K. N. and Swamy, S. N. (2016). Purification and characterization of novel fibrin(ogen)olytic protease from curcuma aromatica salisb.: Role in hemostasis. *Phytomedicine,* 23**,** 1691–1698. <https://doi.org/10.1016/j.phymed.2016.09.007>.

Shrivastava, R., Cucuat, N. and John, G. W. (2007). Effects of alchemilla vulgaris and glycerine on epithelial and myofibroblast cell growth and cutaneous lesion healing in rats. *Phytother Res,* 21**,** 369–73. <https://doi.org/10.1002/ptr.2060>.

Singh, M. K., Rajagopalan, A., Tanimu, H. and Sukumaran, B. O. (2021). Purification, characterization and fibrino(geno)lytic activity of cysteine protease from tabernaemontana divaricata latex. *3 Biotech,* 11**,** 106. <https://doi.org/10.1007/s13205-021-02643-9>.

Singh, M. K., Usha, R., Hithayshree, K. R. and Bindhu, O. S. (2015). Hemostatic potential of latex proteases from tabernaemontana divaricata (l.) r. Br. Ex. Roem. And schult. And artocarpus altilis (parkinson ex. F.A. Zorn) forsberg. *J Thromb Thrombolysis,* 39**,** 43–9. <https://doi.org/10.1007/s11239-013-1012-y>.

Soib, H. H., Ismail, H. F., Husin, F., Abu Bakar, M. H., Yaakob, H. and Sarmidi, M. R. (2020). Bioassay-guided different extraction techniques of carica papaya (linn.) leaves on in vitro wound-healing activities. *Molecules (Basel, Switzerland),* 25**,** 517. <https://doi.org/10.3390/molecules25030517>.

Somavarapu, S., Vemula, S. and Reddy, I. B. (2018). Extraction, purification and characterization of a novel cysteine protease from the latex of plant vallaris solanacea. *Journal of Plant Biochemistry and Biotechnology,* 27**,** 186–198. <https://doi.org/10.1007/s13562-017-0429-3>.

Sultan, M. H., Zuwaiel, A. A., Moni, S. S., Alshahrani, S., Alqahtani, S. S., Madkhali, O. and Elmobark, M. E. (2020). Bioactive principles and potentiality of hot methanolic extract of the leaves from artemisia absinthium l "in vitro cytotoxicity against human mcf-7 breast cancer cells, antibacterial study and wound healing activity". *Curr Pharm Biotechnol,* 21**,** 1711–1721. <https://doi.org/10.2174/1389201021666200928150519>.

Süntar, I., Küpeli Akkol, E., Keles, H., Yesilada, E., Sarker, S. D. and Baykal, T. (2012). Comparative evaluation of traditional prescriptions from cichorium intybus l. For wound healing: Stepwise isolation of an active component by in vivo bioassay and its mode of activity. *J Ethnopharmacol,* 143**,** 299–309. <https://doi.org/10.1016/j.jep.2012.06.036>.

Taleb, S. and Saeedi, M. (2021). The effect of the verbascum thapsus on episiotomy wound healing in nulliparous women: A randomized controlled trial. *BMC Complement Med Ther,* 21**,** 166. <https://doi.org/10.1186/s12906-021-03339-6>.

Tasić-Kostov, M., Arsić, I., Pavlović, D., Stojanović, S., Najman, S., Naumović, S. and Tadić, V. (2019). Towards a modern approach to traditional use: In vitro and in vivo evaluation of alchemilla vulgaris l. Gel wound healing potential. *J Ethnopharmacol,* 238**,** 111789. <https://doi.org/10.1016/j.jep.2019.03.016>.

Teplicki, E., Ma, Q., Castillo, D. E., Zarei, M., Hustad, A. P., Chen, J. and Li, J. (2018). The effects of aloe vera on wound healing in cell proliferation, migration, and viability. *Wounds : a compendium of clinical research and practice,* 30**,** 263–268.

Thirumurthy, P., Natarajan, S., Tharumasivam, S. V., Dhailappan, A., Jayaraj, V. E. C., Doss, A. M. D. C. and Kumar, P. R. (2024). Phytochemical and biological evaluation of scoparia dulcis targeting wound healing efficacy. *African Journal of Biomedical Research,* 27**,** 184–192. <https://doi.org/10.53555/AJBR.v28i3S.7522>.

Tinpun, K., Nakpheng, T., Padmavathi, A. R. and Srichana, T. (2020). In vitro studies of jatropha curcas l. Latex spray formulation for wound healing applications. *Turk J Pharm Sci,* 17**,** 271–279. <https://doi.org/10.4274/tjps.galenos.2019.69875>.

Urs, A. P., Rudresha, G. V., Manjuprasanna, V. N., Suvilesh, K. N., Gowda, M. D. M., Yariswamy, M., Hiremath, V., Ramakrishnan, C., Savitha, M. N., Jayachandra, K., Sharanappa, P. and Vishwanath, B. S. (2019). Plant latex thrombin-like cysteine proteases alleviates bleeding by bypassing factor viii in murine model. *J Cell Biochem,* 120**,** 12843–12858. <https://doi.org/10.1002/jcb.28555>.

Uzuner, H., Yürük, A. and İșoğlu, İ. A. (2025). A potential hemostatic chitosan/gelatin cryogel impregnated with verbascum thapsus leaf extract for noncompressible hemorrhage management. *Biomedical Materials,* 20**,** 065026. <https://doi.org/10.1088/1748-605X/ae19b7>.

Vaisberg, A. J., Milla, M., Planas, M. C., Cordova, J. L., De Agusti, E. R., Ferreyra, R., Mustiga, M. C., Carlin, L. and Hammond, G. B. (1989). Taspine is the cicatrizant principle in sangre de grado extracted from croton lechleri. *Planta Med,* 55**,** 140–3. <https://doi.org/10.1055/s-2006-961907>.

Vijayakumar, G., Sundaram, G. A., Kumar, S. P., Lakshmanan, S., Krishnan, M. and Krishna, V. K. (2024). Comparison of efficacy of topical carica papaya leaf extract and hemocoagulase in postoperative wound healing after therapeutic orthodontic premolar extractions: A split mouth study. *Cureus,* 16**,** e61946. <https://doi.org/10.7759/cureus.61946>.

Wolff, H. H. and Kieser, M. (2007). Hamamelis in children with skin disorders and skin injuries: Results of an observational study. *European Journal of Pediatrics,* 166**,** 943–948. <https://doi.org/10.1007/s00431-006-0363-1>.

Zhang, E., Gao, B., Yang, L., Wu, X. and Wang, Z. (2016). Notoginsenoside ft1 promotes fibroblast proliferation via pi3k/akt/mtor signaling pathway and benefits wound healing in genetically diabetic mice. *J Pharmacol Exp Ther,* 356**,** 324–32. <https://doi.org/10.1124/jpet.115.229369>.

Zouari Bouassida, K., Bardaa, S., Khimiri, M., Rebaii, T., Tounsi, S., Jlaiel, L. and Trigui, M. (2017). Exploring the urtica dioica leaves hemostatic and wound-healing potential. *Biomed Res Int,* 2017**,** 1047523. <https://doi.org/10.1155/2017/1047523>.

Zubair, M., Nybom, H., Lindholm, C., Brandner, J. M. and Rumpunen, K. (2016). Promotion of wound healing by plantago major l. Leaf extracts – ex-vivo experiments confirm experiences from traditional medicine. *Natural Product Research,* 30**,** 622–624. <https://doi.org/10.1080/14786419.2015.1034714>.
