# Supplementary material for: Plant-derived modulators of the hemostasis-wound healing axis: mechanisms, evidence, and translational potential
Source: Front Pharmacol. 2026 Sep 29;17:1902225. doi: 10.3389/fphar.2026.1902225 (PMC13659225; doi:10.3389/fphar.2026.1902225)
Supplement: Supplementary file 5 [file DataSheet5.docx]

**Supplementary Table S5.** Traditional medicinal indications, plant parts used, and preparation or administration methods reported for plants investigated in hemostasis and wound healing.

| **Accepted botanical name and authority (Validated via POWO)** | **Traditional use** | **Plant part used** | **Traditional preparation** | **References** |
| --- | --- | --- | --- | --- |
| *Achillea millefolium* L. [Asteraceae] | Bleeding, ulcers, wounds, gastrointestinal, liver and bile disorders, hepatic diseases, rheumatic pain, pneumonia. | Aerial parts | NR | (Hadavi-Siahboomi et al., 2022, Ghasemi et al., 2023) |
| *Agastache rugosa* (Fisch. & C.A.Mey.) Kuntze [Lamiaceae] | Nausea, vomiting, cholera, miasma and other intestinal disorders described in Traditional Chinese Medicine as ‘dampness’. | Aerial parts | NR | (Cao et al., 2017) |
| *Ageratum conyzoides* L. [Asteraceae] | Bleeding, burns, eczema, headaches, and scalds. | Aerial parts | NR | (Li et al., 2025, Kantesaria et al., 2026) |
| *Alchemilla vulgaris* L. [Rosaceae] | Oral inflammation, epistaxis, furuncles, gynecological and gastrointestinal disorders, wounds, bruises, ulcers, and pruritic vulvar eczema | Aerial parts | Applied alone or incorporated into a topical preparation for wounds, bruises, and ulcers; a water extract incorporated into a wax-, petrolatum-, or lanolin-based ointment is reported for pruritic vulvar eczema. | (Shrivastava et al., 2007, Tasić-Kostov et al., 2019) |
| *Aloe vera* (L.) Burm.f. [Asphodelaceae] | Wound healing, including minor and cutaneous wounds and burns; multipurpose topical treatment of skin conditions. | Fresh *Aloe vera* pulp gel | Fresh leaf gel applied topically to wounds | (Jettanacheawchankit et al., 2009, Teplicki et al., 2018, Al Dhamary et al., 2024, Melenshia et al., 2024, Shin et al., 2024) |
| *Annona senegalensis* Pers. [Annonaceae] | Treatment of snakebite envenomation, particularly Viperidae bites. | Roots and leaves | NR | (Julien et al., 2023) |
| *Artemisia absinthium* L. [Asteraceae] | Antidiabetic, antihypertensive, analgesic, antipyretic, antispasmodic, anti-inflammatory, memory-enhancing, wound-healing, and skin-preserving uses; treatment of cancer in traditional Arabic medicine. | Flowering aerial parts and leaves | Topical ointment. | (Boudjelal et al., 2020, Sultan et al., 2020) |
| *Bletilla striata* (Thunb.) Rchb.f. [Orchidaceae] | Treatment of several health disorders, such as gastrointestinal disorders, peptic ulcer, lung disorders, and traumatic bleeding etc. | Tubers | NR | (Jiang et al., 2021, Lei et al., 2022) |
| *Calendula officinalis* L. [Asteraceae] | Promotion of wound healing and use as an antiseptic and anti-inflammatory agent; hemostasis and bleeding control. | Flowers | Topically applied as balms or creams. | (Duran et al., 2005, Parente et al., 2012, Buzzi et al., 2016, Ayyanahalli Matta et al., 2022) |
| *Calotropis gigantea* (L.) W.T.Aiton [Apocynaceae] | Control of bleeding from fresh cuts and wound healing. | Latex | Topical application of the latex to fresh cuts or wounds. | (Rajesh et al., 2005, Bindhu and Singh, 2014, Urs et al., 2019) |
| *Camellia sinensis* (L.) Kuntze [Theaceae] | General health promotion and treatment of diabetes in ancient China and Southeast Asia | Leaves | Leaf infusion consumed orally | (Asadi et al., 2013, Hashemipour et al., 2017, Kalalinia et al., 2020, Sayahi et al., 2024) |
| *Capsella bursa-pastoris* (L.) Medik. [Brassicaceae] | Chronic heavy menstrual, intermenstrual and postpartum bleeding; hemostasis of fresh wounds, promotion of wound healing, and antiseptic use. | Aerial parts | Mixture with Inula viscosa; further preparation details NR. | (Ghalandari et al., 2017, Naafe et al., 2018, Öztürk et al., 2022) |
| *Carica papaya* L. [Caricaceae] | Bodily ailments, dengue fever, bleeding wounds. | Leaves, latex | NR | (Urs et al., 2019, Soib et al., 2020, Jayasinghe et al., 2022, Vijayakumar et al., 2024) |
| *Chromolaena odorata* (L.) R.M.King & H.Rob. [Asteraceae] | Control of bleeding; treatment of fresh, external and soft-tissue wounds, burn wounds, leech bites, skin infections and dento-alveolitis. | Leaves, stems | Freshly cut leaves; aqueous leaf extracts or decoctions; and topically applied leaf extract. | (Pandith et al., 2012, Sangnim et al., 2022, Li et al., 2024, Abdul Latif et al., 2024) |
| *Cichorium intybus* L. [Asteraceae] | Gastrointestinal disorders, wound healing | Aerial parts, leaves, roots, and leaf or root ashes; leaf-derived callus tissue; seeds. | Root ash mixed with butter to prepare a topical ointment; dried leaves mixed with Anchusa roots, pine resin, and butter. | (Süntar et al., 2012, Gharari et al., 2022, Khalid et al., 2025) |
| *Croton lechleri* Müll.Arg. [Euphorbiaceae] | Fractures, wounds, inflammation, gastrointestinal disorders, rheumatism, circulatory dysfunctions, and cancer | dragon’s blood, described as sap/resin | NR | (Vaisberg et al., 1989, Namjoyan et al., 2016, Rosa et al., 2026) |
| *Curcuma aromatica* Salisb. [Zingiberaceae] | Bleeding from fresh cuts and wound healing. | Rhizome | Applied topically; rhizome powder mixed with clarified butter. | (Shivalingu et al., 2016, Shivalingu et al., 2015) |
| *Curcuma longa* L. [Zingiberaceae] | Bleeding from fresh cuts and wound healing. | Rhizome | Applied topically; rhizome powder mixed with clarified butter. | (Shivalingu et al., 2015) |
| *Ficus drupacea* Thunb. [Moraceae] | Control bleeding from minor injuries and to enhance wound healing activity | Latex | NR | (Manjuprasanna et al., 2021) |
| *Hamamelis virginiana* L. [Hamamelidaceae] | NR | Bark | NR | (Deters et al., 2001, Wolff and Kieser, 2007) |
| *Dittrichia viscosa* (L.) Greuter [Asteraceae] | Antiseptic and hemostatic use. | NR | NR | (Öztürk et al., 2022) |
| *Jatropha curcas* L. [Euphorbiaceae] | Wound healing, burns, dermatitis, inflammation, rash, scabies, sores, syphilis, and rheumatism. | Latex | NR | (Urs et al., 2019, Tinpun et al., 2020) |
| *Myrtus communis* L. [Myrtaceae] | Control of external, internal, vaginal, and gingival bleeding; wound healing. | Leaves and fruits. | Berries administered orally or leaves prepared as a decoction for internal and vaginal bleeding; fresh pounded leaves used as a poultice or leaf powder applied topically for external bleeding, including gingival bleeding. | (Ebrahimi et al., 2020, Khodaie et al., 2021, Jafari et al., 2024) |
| *Oxystelma esculentum* (L.f.) Sm. [Apocynaceae] | Bleeding wounds | Latex | NR | (Urs et al., 2019) |
| *Panax notoginseng* (Burkill) F.H.Chen [Araliaceae] | Promoting blood circulation. | NR | NR | (Gao et al., 2014, Zhang et al., 2016, Men et al., 2020, Lei et al., 2022) |
| *Plantago major* L. [Plantaginaceae] | Wound healing. | Leaves | NR | (Zubair et al., 2016, Ghanadian et al., 2024) |
| *Potentilla erecta* (L.) Raeusch. [Rosaceae] | Wound and ulcer healing; treatment of diarrhea and mild inflammation of the oral mucosa. | Roots; rhizome/underground parts. | Extract of the underground parts for diarrhea and mild oral-mucosal inflammation | (Kaltalioglu et al., 2020, Marcinczyk et al., 2021) |
| *Scoparia dulcis* L. [Plantaginaceae] | Arrest of bleeding from cuts and bruises; wound healing and skin disorders; diabetes mellitus; fever; insect bites; dysentery; and unspecified urinary disorders. | Whole plant, leaves | Crushed plant applied topically to cuts and bruises | (Ediriweera et al., 2011, Thirumurthy et al., 2024) |
| *Symphytum officinale* L. [Boraginaceae] | Wound healing and external skin disorders; fractures and bone injuries, sprains, rheumatism, gout, hematomas, thrombophlebitis, hepatic disorders, gastritis, and ulcers. | Leaves, roots | Root extract for skin disorders; leaf- or root-containing creams for superficial wounds; tea for hepatic disorders and for gastritis and ulcers. | (Araújo et al., 2012, Mârza et al., 2024) |
| *Tabernaemontana divaricata* (L.) R.Br. ex Roem. & Schult. [Apocynaceae] | Wound healing and fresh cuts; fever, pain, dysentery, bleeding wounds and minor injuries. | Latex | Topical application for wounds. | (Singh et al., 2015, Urs et al., 2019, Singh et al., 2021) |
| *Tridax procumbens* L. [Asteraceae] | Treatment of open wounds, arrest of bleeding, and promotion of wound healing. | Leaves | Leaf extract applied topically to open wounds. | (Alshargabi et al., 2023, Gubbiveeranna et al., 2024) |
| *Urtica dioica* L. [Urticaceae] | Epistaxis and excessive menstrual bleeding; external treatment of unspecified skin problems. | Leaves, aerial parts | Powdered leaf preparation or extract. | (Zouari Bouassida et al., 2017, Kasouni et al., 2021) |
| *Vallaris solanacea* (Roth ex Roem. & Schult.) Kuntze [Apocynaceae] | NR | Latex | NR | (Somavarapu et al., 2018, Urs et al., 2019) |
| *Verbascum sinuatum* L. [Scrophulariaceae] | NR | Leaves | NR | (Abdallah et al., 2022) |
| *Verbascum thapsus* L. [Scrophulariaceae] | Treatment and healing of wounds. Traditional analgesic, antibacterial, anti-inflammatory and wound-healing effects. | Flowers, leaves | NR | (Taleb and Saeedi, 2021, Uzuner et al., 2025) |

Traditional uses are presented as documented in the source literature and should not be interpreted as evidence of clinical efficacy or safety. NR, not reported; POWO, Plants of the World Online ([**http://www.plantsoftheworldonline.org**](http://www.plantsoftheworldonline.org/))

References

Abdallah, L., Surakji, I., Qawasme, T., Ayyash, D., Shhadeh, R., Omar, G. and Barakat, A. (2022). In vitro activity of some medicinal plants on blood coagulation. *Turk J Pharm Sci,* 19**,** 330–335. <https://doi.org/10.4274/tjps.galenos.2021.14603>.

Abdul Latif, M., Mustafa, A., Keong, L. C. and Hamid, A. (2024). Chromolaena odorata layered-nitrile rubber polymer transdermal patch enhanced wound healing in vivo. *PLoS One,* 19**,** e0295381. <https://doi.org/10.1371/journal.pone.0295381>.

Al Dhamary, N., Al Adhal, A., Kadi, H. and Al Kamarany, M. A. (2024). Investigation effect of aloe vera in fresh gel form on rabbit’s model wound. *Journal of Complementary and Alternative Medical Research,* 25**,** 17–26. <https://doi.org/10.9734/jocamr/2024/v25i3522>.

Alshargabi, A., Mansour, W. and Al-Madhagi, W. M. (2023). Effect of tridax procumbens extracts on coagulation time according to gender and blood groups a and o: In vitro study. *Journal of Herbal Medicine,* 42**,** 100806. <https://doi.org/10.1016/j.hermed.2023.100806>.

Araújo, L. U., Reis, P. G., Barbosa, L. C., Saúde-Guimarães, D. A., Grabe-Guimarães, A., Mosqueira, V. C., Carneiro, C. M. and Silva-Barcellos, N. M. (2012). In vivo wound healing effects of symphytum officinale l. Leaves extract in different topical formulations. *Pharmazie,* 67**,** 355–60.

Asadi, S. Y., Parsaei, P., Karimi, M., Ezzati, S., Zamiri, A., Mohammadizadeh, F. and Rafieian-Kopaei, M. (2013). Effect of green tea (camellia sinensis) extract on healing process of surgical wounds in rat. *International Journal of Surgery,* 11**,** 332–337. <https://doi.org/10.1016/j.ijsu.2013.02.014>.

Ayyanahalli Matta, B. K., Kumar, S., Mehta, C. H., Nayak, U. Y. and Rodriguez, P. G. (2022). Comparative evaluation of the effectiveness of a combination of absorbable gelatin sponge and calendula officinalis with absorbable gelatin sponge used alone as a hemostatic agent-an in-vitro study. *Dent J (Basel),* 10**,** 76. <https://doi.org/10.3390/dj10050076>.

Bindhu, O. S. and Singh, M. K. (2014). Hemostatic, milk clotting and blood stain removal potential of cysteine proteases from calotropis gigantea (l.) r. Br. Latex. *Pharmacogn Mag,* 10**,** S350–6. <https://doi.org/10.4103/0973-1296.133294>.

Boudjelal, A., Smeriglio, A., Ginestra, G., Denaro, M. and Trombetta, D. (2020). Phytochemical profile, safety assessment and wound healing activity of artemisia absinthium l. *Plants (Basel),* 9**,** 1744. <https://doi.org/10.3390/plants9121744>.

Buzzi, M., De Freitas, F. and De Barros Winter, M. (2016). Therapeutic effectiveness of a calendula officinalis extract in venous leg ulcer healing. *J Wound Care,* 25**,** 732–739. <https://doi.org/10.12968/jowc.2016.25.12.732>.

Cao, P., Xie, P., Wang, X., Wang, J., Wei, J. and Kang, W. Y. (2017). Chemical constituents and coagulation activity of agastache rugosa. *BMC Complement Altern Med,* 17**,** 93. <https://doi.org/10.1186/s12906-017-1592-8>.

Deters, A., Dauer, A., Schnetz, E., Fartasch, M. and Hensel, A. (2001). High molecular compounds (polysaccharides and proanthocyanidins) from hamamelis virginiana bark: Influence on human skin keratinocyte proliferation and differentiation and influence on irritated skin. *Phytochemistry,* 58**,** 949–958. <https://doi.org/10.1016/S0031-9422(01)00361-2>.

Duran, V., Matic, M., Jovanovć, M., Mimica, N., Gajinov, Z., Poljacki, M. and Boza, P. (2005). Results of the clinical examination of an ointment with marigold (calendula officinalis) extract in the treatment of venous leg ulcers. *Int J Tissue React,* 27**,** 101–6.

Ebrahimi, F., Mahmoudi, J., Torbati, M., Karimi, P. and Valizadeh, H. (2020). Hemostatic activity of aqueous extract of myrtus communis l. Leaf in topical formulation: In vivo and in vitro evaluations. *J Ethnopharmacol,* 249**,** 112398. <https://doi.org/10.1016/j.jep.2019.112398>.

Ediriweera, E. R., Jayakody, J. R. and Ratnasooriya, W. D. (2011). Pro blood clotting activity of scoparia dulcis in rats. *Ayu,* 32**,** 271–4. <https://doi.org/10.4103/0974-8520.92547>.

Gao, B., Huang, L., Liu, H., Wu, H., Zhang, E., Yang, L., Wu, X. and Wang, Z. (2014). Platelet p2y₁₂ receptors are involved in the haemostatic effect of notoginsenoside ft1, a saponin isolated from panax notoginseng. *Br J Pharmacol,* 171**,** 214–23. <https://doi.org/10.1111/bph.12435>.

Ghalandari, S., Kariman, N., Sheikhan, Z., Mojab, F., Mirzaei, M. and Shahrahmani, H. (2017). Effect of hydroalcoholic extract of capsella bursa pastoris on early postpartum hemorrhage: A clinical trial study. *J Altern Complement Med,* 23**,** 794–799. <https://doi.org/10.1089/acm.2017.0095>.

Ghanadian, M., Soltani, R., Homayouni, A., Khorvash, F., Jouabadi, S. M. and Abdollahzadeh, M. (2024). The effect of plantago major hydroalcoholic extract on the healing of diabetic foot and pressure ulcers: A randomized open-label controlled clinical trial. *Int J Low Extrem Wounds,* 23**,** 475–481. <https://doi.org/10.1177/15347346211070723>.

Gharari, Z., Hanachi, P., Sadeghinia, H. and Walker, T. R. (2022). Cichorium intybus bio-callus synthesized silver nanoparticles: A promising antioxidant, antibacterial and anticancer compound. *International Journal of Pharmaceutics,* 625**,** 122062. <https://doi.org/10.1016/j.ijpharm.2022.122062>.

Ghasemi, M. R., Ranjbar, A., Tamri, P., Pourmoslemi, S., Nourian, A. and Dastan, D. (2023). In vitro antibacterial activity and wound healing effects of achillea millefolium essential oil in rat. *J Pharmacopuncture,* 26**,** 167–174. <https://doi.org/10.3831/kpi.2023.26.2.167>.

Gubbiveeranna, V., Megha, G. T., Kusuma, C. G., Ravikumar, H., Thoyajakshi, R. S., Vijayakumar, S., Mathad, S. N., Nagaraju, S., Wazzan, H., Khan, A., Alzahrani, K. A. and Malash, A. M. (2024). Effect of 'procumbenase' a serine protease from tridax procumbens aqueous extract on wound healing: A scar free healing of full thickness wounds. *Int J Biol Macromol,* 273**,** 133147. <https://doi.org/10.1016/j.ijbiomac.2024.133147>.

Hadavi-Siahboomi, M., Yegdaneh, A., Talebi, A. and Minaiyan, M. (2022). Ulcer-healing effect of hydroalcoholic extract and essential oil of achillea millefolium l. On murine model of colitis. *Int J Prev Med,* 13**,** 155. <https://doi.org/10.4103/ijpvm.ijpvm_50_22>.

Hashemipour, M. A., Lotfi, S., Torabi, M., Sharifi, F., Ansari, M., Ghassemi, A. and Sheikhshoaie, S. (2017). Evaluation of the effects of three plant species (myrtus communis l., camellia sinensis l., zataria multiflora boiss.) on the healing process of intraoral ulcers in rats. *J Dent (Shiraz),* 18**,** 127–135.

Jafari, Z., Bardania, H., Barmak, M. J., Eslami, S., Mahmoudi-Mourderaz, Y., Roustaei, N., Talebianpoor, M. S., Kokhdan, E. P. and Khoramrooz, S. S. (2024). Antimicrobial, anti-inflammatory, and wound healing properties of myrtus communis leaf methanolic extract ointment on burn wound infection induced by methicillin-resistant staphylococcus aureus in rats. *BioMed Research International,* 2024**,** 6758817. <https://doi.org/10.1155/2024/6758817>.

Jayasinghe, C. D., Ratnasooriya, W. D., Premakumara, S. and Udagama, P. V. (2022). Platelet augmentation activity of mature leaf juice of sri lankan wild type cultivar of carica papaya l: Insights into potential cellular mechanisms. *J Ethnopharmacol,* 296**,** 115511. <https://doi.org/10.1016/j.jep.2022.115511>.

Jettanacheawchankit, S., Sasithanasate, S., Sangvanich, P., Banlunara, W. and Thunyakitpisal, P. (2009). Acemannan stimulates gingival fibroblast proliferation; expressions of keratinocyte growth factor-1, vascular endothelial growth factor, and type i collagen; and wound healing. *Journal of pharmacological sciences,* 109**,** 525–531. <https://doi.org/10.1254/jphs.08204fp>.

Jiang, S., Wang, M., Jiang, L., Xie, Q., Yuan, H., Yang, Y., Zafar, S., Liu, Y., Jian, Y., Li, B. and Wang, W. (2021). The medicinal uses of the genus bletilla in traditional chinese medicine: A phytochemical and pharmacological review. *Journal of Ethnopharmacology,* 280**,** 114263. <https://doi.org/https://doi.org/10.1016/j.jep.2021.114263>.

Julien, G. K., Marcel, A. G., Antoinette, Y.-C. C. C., Hermann, A. M., Benoit, Y. D. C., Bernard, A. K., Karamoko, O., Mireille, D. and Joseph, D. A. (2023). An in-vitro study of the effect of hydroethanol extract of annona senegalensis leaves on bitis rhinoceros (viperidae) venom induced coagulopathy. *European Journal of Medicinal Plants,* 34**,** 33–41. <https://doi.org/10.9734/ejmp/2023/v34i11118>.

Kalalinia, F., Amiri, N., Mehrvarzian, N., Fazly Bazzaz, B. S., Iranshahi, M., Shahroodi, A., Arabzadeh, S., Abbaspour, M., Badiee Aaval, S. and Movaffagh, J. (2020). Topical green tea formulation with anti-hemorrhagic and antibacterial effects. *Iran J Basic Med Sci,* 23**,** 1085–1090. <https://doi.org/10.22038/ijbms.2020.41397.9782>.

Kaltalioglu, K., Balabanli, B. and Coskun-Cevher, S. (2020). Phenolic, antioxidant, antimicrobial, and in-vivo wound healing properties of potentilla erecta l. Root extract in diabetic rats. *Iran J Pharm Res,* 19**,** 264–274. <https://doi.org/10.22037/ijpr.2019.112411.13742>.

Kantesaria, R., Bagra, Y., Varma, P. and Panda, H. S. (2026). Plasticization of an ageratum conyzoides natural extract in crosslinked hydrogel: A promising material for combating wound infection. *Appl Biochem Biotechnol,* 198**,** 2750–2766. <https://doi.org/10.1007/s12010-026-05594-0>.

Kasouni, A. I., Chatzimitakos, T. G., Stalikas, C. D., Trangas, T., Papoudou-Bai, A. and Troganis, A. N. (2021). The unexplored wound healing activity of urtica dioica l. Extract: An in vitro and in vivo study. *Molecules,* 26**,** 6248. <https://doi.org/10.3390/molecules26206248>.

Khalid, A., Khurshid, S., Uroos, M., Khan, J., Sarwar, A., Ul Haq, E., Aziz, T., Alshehri, F., Al-Asmari, F., Baothman, B. K., Al-Joufi, F. A. and Alwethaynani, M. S. (2025). Green synthesis of silver and gold nanoparticles using seeds extract of cichorium intybus and their comparative analysis with commercially available ointment for wound healing activity. *J Mol Histol,* 56**,** 145. <https://doi.org/10.1007/s10735-025-10426-2>.

Khodaie, S. A., Emadi, F., Naseri, M., Kamalinejad, M., Riahi, S. M., Alijaniha, F. and Roghani, M. (2021). The effect of myrtus communis aqueous extract-containing gel on wound healing in streptozotocin-induced diabetic rats. *Curr Drug Discov Technol,* 18**,** 542–547. <https://doi.org/10.2174/1570163817666200712163956>.

Lei, T., Gao, Y., Duan, Y., Cui, C., Zhang, L. and Si, M. (2022). Panax notoginseng saponins improves healing of high glucose-induced wound through the gsk-3β/β-catenin pathway. *Environ Toxicol,* 37**,** 1867–1877. <https://doi.org/10.1002/tox.23533>.

Li, H.-F., Feng, H., Wang, Y., Pan, Z.-C., Yin, L., Qiu, H.-L., Qiao, H., Zhao, J.-Q., Xia, X.-Y., Hou, J.-C. and Wang, R.-X. (2024). Evaluation of hemostatic, anti-inflammatory, wound healing, skin irritation and allergy, and antimicrobial properties of active fraction from the ethanol extract of chromolaena odorata (l.) r.M. King & h. Rob. *Journal of Ethnopharmacology,* 331**,** 118330. <https://doi.org/10.1016/j.jep.2024.118330>.

Li, Y., Tan, Y., Zhao, H., Chen, S., Nilghaz, A., Cao, R. and Zhou, S. (2025). Green biosynthetic silver nanoparticles from ageratum conyzoides as multifunctional hemostatic agents: Combining hemostasis, antibacterial, and anti-inflammatory properties for effective wound healing. *Mater Today Bio,* 31**,** 101468. <https://doi.org/10.1016/j.mtbio.2025.101468>.

Manjuprasanna, V. N., Urs, A. P., Rudresha, G. V., Milan Gowda, M. D., Jayachandra, K., Hiremath, V., Rajaiah, R. and Vishwanath, B. S. (2021). Drupin, a thrombin-like protease prompts platelet activation and aggregation through protease-activated receptors. *J Cell Biochem,* 122**,** 870–881. <https://doi.org/10.1002/jcb.29917>.

Marcinczyk, N., Gołaszewska, A., Gromotowicz-Poplawska, A., Misztal, T., Strawa, J., Tomczyk, M., Kasacka, I. and Chabielska, E. (2021). Multidirectional effects of tormentil extract on hemostasis in experimental diabetes. *Front Pharmacol,* 12**,** 682987. <https://doi.org/10.3389/fphar.2021.682987>.

Mârza, S. M., Dăescu, A. M., Purdoiu, R. C., Dragomir, M., Tătaru, M., Melega, I., Nagy, A. L., Gal, A., Tăbăran, F., Bogdan, S., Moldovan, M., Pall, E., Munteanu, C., Magyari, K. and Papuc, I. (2024). Healing of skin wounds in rats using creams based on symphytum officinale extract. *Int J Mol Sci,* 25**,** 3099. <https://doi.org/10.3390/ijms25063099>.

Melenshia, D. S., Amirtham, S. M., Rebekah, G., Vinod, E. and Kachroo, U. (2024). Effect of reconstituted, lyophilized cold aqueous extract of aloe vera on human whole blood clotting time - a pilot study. *J Ayurveda Integr Med,* 15**,** 100887. <https://doi.org/10.1016/j.jaim.2024.100887>.

Men, S. Y., Huo, Q. L., Shi, L., Yan, Y., Yang, C. C., Yu, W. and Liu, B. Q. (2020). Panax notoginseng saponins promotes cutaneous wound healing and suppresses scar formation in mice. *J Cosmet Dermatol,* 19**,** 529–534. <https://doi.org/10.1111/jocd.13042>.

Naafe, M., Kariman, N., Keshavarz, Z., Khademi, N., Mojab, F. and Mohammadbeigi, A. (2018). Effect of hydroalcoholic extracts of capsella bursa-pastoris on heavy menstrual bleeding: A randomized clinical trial. *J Altern Complement Med,* 24**,** 694–700. <https://doi.org/10.1089/acm.2017.0267>.

Namjoyan, F., Kiashi, F., Moosavi, Z. B., Saffari, F. and Makhmalzadeh, B. S. (2016). Efficacy of dragon's blood cream on wound healing: A randomized, double-blind, placebo-controlled clinical trial. *J Tradit Complement Med,* 6**,** 37–40. <https://doi.org/10.1016/j.jtcme.2014.11.029>.

Öztürk, O. U., Ugur, M., Güzel, Y., Öztürk, M. A., Gürsoy, D., Doğan, S. and Temiz, M. (2022). Hemostatic effects of traditional inula viscosa and capsella bursa-pastoris plant mixture extract on rat liver parenchymal bleeding model. *Ulus Travma Acil Cerrahi Derg,* 28**,** 1059–1065. <https://doi.org/10.14744/tjtes.2022.90838>.

Pandith, H., Thongpraditchote, S., Wongkrajang, Y. and Gritsanapan, W. (2012). In vivo and in vitro hemostatic activity of chromolaena odorata leaf extract. *Pharm Biol,* 50**,** 1073–7. <https://doi.org/10.3109/13880209.2012.656849>.

Parente, L. M., Lino Júnior Rde, S., Tresvenzol, L. M., Vinaud, M. C., De Paula, J. R. and Paulo, N. M. (2012). Wound healing and anti-inflammatory effect in animal models of calendula officinalis l. Growing in brazil. *Evid Based Complement Alternat Med,* 2012**,** 375671. <https://doi.org/10.1155/2012/375671>.

Rajesh, R., Raghavendra Gowda, C. D., Nataraju, A., Dhananjaya, B. L., Kemparaju, K. and Vishwanath, B. S. (2005). Procoagulant activity of calotropis gigantea latex associated with fibrin(ogen)olytic activity. *Toxicon,* 46**,** 84–92. <https://doi.org/10.1016/j.toxicon.2005.03.012>.

Rosa, M. C. D. B., Campos, M. E. D. S. T., Albuquerque, A. S., Braz, M. S., Mapele, R. D. O., De Oliveira, M. C. G., Toma, H. S., Ferraz, P. F. P., Pereira, R. N. and Raymundo, D. L. (2026). Effect of croton lechleri sap-based topical treatment, with or without nitroglycerin, on healing of equine metatarsal skin wounds. *Veterinary and Animal Science,* 32**,** 100598. <https://doi.org/10.1016/j.vas.2026.100598>.

Sangnim, T., Meeboon, P., Phongsewalak, P., Prasongdee, P., Sriamornsak, P., Singh, I., Manmuan, S. and Huanbutta, K. (2022). Development and evaluation of liquid plaster loaded with chromolaena odorata leaf extract endowed with several beneficial properties to wound healing. *Gels (Basel, Switzerland)* 8**,** 72. <https://doi.org/10.3390/gels8020072>.

Sayahi, M., Jahangirimehr, A., Hatami Manesh, Z., Mojab, F. and Nikbina, M. (2024). The effect of camellia sinensis ointment on perineal pain and episiotomy wound healing in primiparous women: A triple-blind randomized clinical trial. *PLoS One,* 19**,** e0305048. <https://doi.org/10.1371/journal.pone.0305048>.

Shin, B., Hillyer, T. and Shin, W. S. (2024). Rational design and testing of antibacterial aloe vera hemostatic hydrogel. *Gels,* 10**,** 409. <https://doi.org/10.3390/gels10060409>.

Shivalingu, B. R., Vivek, H. K., Nafeesa, Z., Priya, B. S. and Swamy, S. N. (2015). Comparative analysis of procoagulant and fibrinogenolytic activity of crude protease fractions of turmeric species. *Journal of Ethnopharmacology,* 172**,** 261–264. <https://doi.org/10.1016/j.jep.2015.06.018>.

Shivalingu, B. R., Vivek, H. K., Priya, B. S., Soujanya, K. N. and Swamy, S. N. (2016). Purification and characterization of novel fibrin(ogen)olytic protease from curcuma aromatica salisb.: Role in hemostasis. *Phytomedicine,* 23**,** 1691–1698. <https://doi.org/10.1016/j.phymed.2016.09.007>.

Shrivastava, R., Cucuat, N. and John, G. W. (2007). Effects of alchemilla vulgaris and glycerine on epithelial and myofibroblast cell growth and cutaneous lesion healing in rats. *Phytother Res,* 21**,** 369–73. <https://doi.org/10.1002/ptr.2060>.

Singh, M. K., Rajagopalan, A., Tanimu, H. and Sukumaran, B. O. (2021). Purification, characterization and fibrino(geno)lytic activity of cysteine protease from tabernaemontana divaricata latex. *3 Biotech,* 11**,** 106. <https://doi.org/10.1007/s13205-021-02643-9>.

Singh, M. K., Usha, R., Hithayshree, K. R. and Bindhu, O. S. (2015). Hemostatic potential of latex proteases from tabernaemontana divaricata (l.) r. Br. Ex. Roem. And schult. And artocarpus altilis (parkinson ex. F.A. Zorn) forsberg. *J Thromb Thrombolysis,* 39**,** 43–9. <https://doi.org/10.1007/s11239-013-1012-y>.

Soib, H. H., Ismail, H. F., Husin, F., Abu Bakar, M. H., Yaakob, H. and Sarmidi, M. R. (2020). Bioassay-guided different extraction techniques of carica papaya (linn.) leaves on in vitro wound-healing activities. *Molecules (Basel, Switzerland),* 25**,** 517. <https://doi.org/10.3390/molecules25030517>.

Somavarapu, S., Vemula, S. and Reddy, I. B. (2018). Extraction, purification and characterization of a novel cysteine protease from the latex of plant vallaris solanacea. *Journal of Plant Biochemistry and Biotechnology,* 27**,** 186–198. <https://doi.org/10.1007/s13562-017-0429-3>.

Sultan, M. H., Zuwaiel, A. A., Moni, S. S., Alshahrani, S., Alqahtani, S. S., Madkhali, O. and Elmobark, M. E. (2020). Bioactive principles and potentiality of hot methanolic extract of the leaves from artemisia absinthium l "in vitro cytotoxicity against human mcf-7 breast cancer cells, antibacterial study and wound healing activity". *Curr Pharm Biotechnol,* 21**,** 1711–1721. <https://doi.org/10.2174/1389201021666200928150519>.

Süntar, I., Küpeli Akkol, E., Keles, H., Yesilada, E., Sarker, S. D. and Baykal, T. (2012). Comparative evaluation of traditional prescriptions from cichorium intybus l. For wound healing: Stepwise isolation of an active component by in vivo bioassay and its mode of activity. *J Ethnopharmacol,* 143**,** 299–309. <https://doi.org/10.1016/j.jep.2012.06.036>.

Taleb, S. and Saeedi, M. (2021). The effect of the verbascum thapsus on episiotomy wound healing in nulliparous women: A randomized controlled trial. *BMC Complement Med Ther,* 21**,** 166. <https://doi.org/10.1186/s12906-021-03339-6>.

Tasić-Kostov, M., Arsić, I., Pavlović, D., Stojanović, S., Najman, S., Naumović, S. and Tadić, V. (2019). Towards a modern approach to traditional use: In vitro and in vivo evaluation of alchemilla vulgaris l. Gel wound healing potential. *J Ethnopharmacol,* 238**,** 111789. <https://doi.org/10.1016/j.jep.2019.03.016>.

Teplicki, E., Ma, Q., Castillo, D. E., Zarei, M., Hustad, A. P., Chen, J. and Li, J. (2018). The effects of aloe vera on wound healing in cell proliferation, migration, and viability. *Wounds : a compendium of clinical research and practice,* 30**,** 263–268.

Thirumurthy, P., Natarajan, S., Tharumasivam, S. V., Dhailappan, A., Jayaraj, V. E. C., Doss, A. M. D. C. and Kumar, P. R. (2024). Phytochemical and biological evaluation of scoparia dulcis targeting wound healing efficacy. *African Journal of Biomedical Research,* 27**,** 184–192. <https://doi.org/10.53555/AJBR.v28i3S.7522>.

Tinpun, K., Nakpheng, T., Padmavathi, A. R. and Srichana, T. (2020). In vitro studies of jatropha curcas l. Latex spray formulation for wound healing applications. *Turk J Pharm Sci,* 17**,** 271–279. <https://doi.org/10.4274/tjps.galenos.2019.69875>.

Urs, A. P., Rudresha, G. V., Manjuprasanna, V. N., Suvilesh, K. N., Gowda, M. D. M., Yariswamy, M., Hiremath, V., Ramakrishnan, C., Savitha, M. N., Jayachandra, K., Sharanappa, P. and Vishwanath, B. S. (2019). Plant latex thrombin-like cysteine proteases alleviates bleeding by bypassing factor viii in murine model. *J Cell Biochem,* 120**,** 12843–12858. <https://doi.org/10.1002/jcb.28555>.

Uzuner, H., Yürük, A. and İșoğlu, İ. A. (2025). A potential hemostatic chitosan/gelatin cryogel impregnated with verbascum thapsus leaf extract for noncompressible hemorrhage management. *Biomedical Materials,* 20**,** 065026. <https://doi.org/10.1088/1748-605X/ae19b7>.

Vaisberg, A. J., Milla, M., Planas, M. C., Cordova, J. L., De Agusti, E. R., Ferreyra, R., Mustiga, M. C., Carlin, L. and Hammond, G. B. (1989). Taspine is the cicatrizant principle in sangre de grado extracted from croton lechleri. *Planta Med,* 55**,** 140–3. <https://doi.org/10.1055/s-2006-961907>.

Vijayakumar, G., Sundaram, G. A., Kumar, S. P., Lakshmanan, S., Krishnan, M. and Krishna, V. K. (2024). Comparison of efficacy of topical carica papaya leaf extract and hemocoagulase in postoperative wound healing after therapeutic orthodontic premolar extractions: A split mouth study. *Cureus,* 16**,** e61946. <https://doi.org/10.7759/cureus.61946>.

Wolff, H. H. and Kieser, M. (2007). Hamamelis in children with skin disorders and skin injuries: Results of an observational study. *European Journal of Pediatrics,* 166**,** 943–948. <https://doi.org/10.1007/s00431-006-0363-1>.

Zhang, E., Gao, B., Yang, L., Wu, X. and Wang, Z. (2016). Notoginsenoside ft1 promotes fibroblast proliferation via pi3k/akt/mtor signaling pathway and benefits wound healing in genetically diabetic mice. *J Pharmacol Exp Ther,* 356**,** 324–32. <https://doi.org/10.1124/jpet.115.229369>.

Zouari Bouassida, K., Bardaa, S., Khimiri, M., Rebaii, T., Tounsi, S., Jlaiel, L. and Trigui, M. (2017). Exploring the urtica dioica leaves hemostatic and wound-healing potential. *Biomed Res Int,* 2017**,** 1047523. <https://doi.org/10.1155/2017/1047523>.

Zubair, M., Nybom, H., Lindholm, C., Brandner, J. M. and Rumpunen, K. (2016). Promotion of wound healing by plantago major l. Leaf extracts – ex-vivo experiments confirm experiences from traditional medicine. *Natural Product Research,* 30**,** 622–624. <https://doi.org/10.1080/14786419.2015.1034714>.
